# *Poglut2/3* double knockout in mice results in neonatal lethality with reduced levels of fibrillin in lung tissues

**DOI:** 10.1016/j.jbc.2024.107445

**Published:** 2024-06-04

**Authors:** Sanjiv Neupane, Daniel B. Williamson, Robyn A. Roth, Carmen M. Halabi, Robert S. Haltiwanger, Bernadette C. Holdener

**Affiliations:** 1 Department of Biochemistry and Cell Biology, Stony Brook University, Stony Brook, New York, USA; 2 Complex Carbohydrate Research Center, Department of Biochemistry and Molecular Biology, University of Georgia, Athens, Georgia, USA; 3 Division of Nephrology, Department of Pediatrics, Washington University School of Medicine, St Louis, Missouri, USA

**Keywords:** glycosyltransferases, fibrillins, microfibrils, *O*-linked glucose, lung

## Abstract

Fibrillin microfibrils play a critical role in the formation of elastic fibers, tissue/organ development, and cardiopulmonary function. These microfibrils not only provide structural support and flexibility to tissues, but they also regulate growth factor signaling through a plethora of microfibril-binding proteins in the extracellular space. Mutations in fibrillins are associated with human diseases affecting cardiovascular, pulmonary, skeletal, and ocular systems. Fibrillins consist of up to 47 epidermal growth factor-like repeats, of which more than half are modified by protein *O*-glucosyltransferase 2 (POGLUT2) and/or POGLUT3. Loss of these modifications reduces secretion of N-terminal fibrillin constructs overexpressed *in vitro.* Here, we investigated the role of POGLUT2 and POGLUT3 *in vivo* using a *Poglut2/3* double knockout *(DKO)* mouse model. Blocking *O-*glucosylation caused neonatal death with skeletal, pulmonary, and eye defects reminiscent of fibrillin/elastin mutations. Proteomic analyses of *DKO* dermal fibroblast medium and extracellular matrix provided evidence that fibrillins were more sensitive to loss of *O*-glucose compared to other POGLUT2/3 substrates. This conclusion was supported by immunofluorescent analyses of late gestation *DKO* lungs where FBN levels were reduced and microfibrils appeared fragmented in the pulmonary arteries and veins, bronchioles, and developing saccules. Defects in fibrillin microfibrils likely contributed to impaired elastic fiber formation and histological changes observed in *DKO* lung blood vessels, bronchioles, and saccules. Collectively, these results highlight the importance of POGLUT2/3-mediated *O*-glucosylation *in vivo* and open the possibility that *O-*glucose modifications on fibrillin influence microfibril assembly and or protein interactions in the ECM environment.

Fibrillins 1 and 2 (FBN1 and FBN2) are major components of extracellular matrix (ECM) microfibrils (1) and provide a scaffold for elastic fiber formation (reviewed in (2, 3, 4)). Elastogenesis can be facilitated through FBN microfibril interactions with latent transforming growth factor beta (TGF-β) binding protein-4 (LTBP-4) and fibulins 4 and 5 (FBLN4, -5) (5, 6). In non-elastic tissues in the eye, FBN interacts with LTBP2 to form microfibrils that anchor the lens (7). Apart from providing elasticity and structural support to tissues, FBN microfibrils also modulate growth factor signaling through interactions with LTBP1, -3, -4, bone morphogenetic proteins (BMPs), and growth and differentiation factors (GDF) (2, 3, 4, 8, 9). In humans, mutations in *FBN1* can cause Marfan syndrome (MFS), one of the most extensively studied connective tissue disorders that impact the musculoskeletal, cardiovascular, pulmonary, ocular, and craniofacial organs (reviewed in (10, 11)). A subset of *FBN1* genetic mutations can cause acromelic dysplasias, a group of musculoskeletal disorders that share extremely short stature and stiff joint phenotypes (12). These symptoms contrast with MFS where patients are commonly taller than average and have joint hypermobility (13). The pathology of both MFS and acromelic dysplasias is largely associated with dysregulation of TGF-β superfamily signaling (13), highlighting the importance of FBN function in the ECM for regulating extracellular growth factor signaling.

One prevalent feature of both FBNs and LTBPs is their multiple Epidermal Growth Factor-like repeats (EGFs) (4, 14). Recently, more than half of the EGFs in FBN1, FBN2, and LTBP1 were shown to be modified by Protein *O*-glucosyltransferase-2 and/or −3 (POGLUT2 and POGLUT3) with an oxygen-linked (*O-*linked) glucose (*O-*glucose). These modifications are located on a serine residue within the putative consensus sequence, C^3^-x-N-T-x-G-S-F/Y-x-C^4^, between the third (C^3^) and the fourth (C^4^) cysteine of an EGF (14, 15). These studies revealed that POGLUT2/3 adds glucose to a folded EGF in the endoplasmic reticulum and is needed for efficient secretion of overexpressed FBN1 fragments in HEK293T cells (14, 15). FBN1-2 are unique among the 56 predicted POGLUT2/3 substrates because of their large number of EGFs (47 in total) of which ∼50% are modified by POGLUT2/3. For comparison, POGLUT2/3 modify 11 of LTBP1’s 18 EGFs and only one of NOTCH1’s 36 EGFs (14, 15). Additionally, FBNs are a foundational part of the ECM to which many predicted POGLUT2/3 substrates bind and whose functions are FBN-dependent (8). Despite the abundance of *O-*glucosylated EGFs in the FBNs and the critical role that these EGFs play in modulating FBN inter and intramolecular interactions (4, 16, 17, 18, 19), we do not know whether the *O-*glucose modifications are critical for *in vivo* trafficking and/or extracellular protein function. Similar *O-*glycans added to NOTCH1 EGFs by Protein *O-*Fucosyltransferase 1 (POFUT1) and Protein *O-*Glucosyltransferase 1 (POGLUT1) not only influence receptor folding and secretion but also influence signaling by modulating extracellular receptor-ligand interactions (20, 21). POFUT1 and POGLUT1 each modify ∼50% of NOTCH1 EGFs, which is comparable to the percentage of FBN EGFs modified by POGLUT2/3 (14, 20, 22) and suggests a similar role for POGLUT2/3-mediated *O-*glucosylation.

In this study, we found that POGLUT2/3-mediated *O-*glucosylation of EGFs was essential for the survival of newborn mice. *Poglut2/3* double knockouts (*Poglut2/3 DKOs*) were runted with syndactyly and had eye and lung defects similar to phenotypes described in *Fbn1, Fbn2,* and *Ltbp* mutant mice (reviewed in (9, 13, 23)). These shared abnormalities suggested that the *Poglut2/3 DKO* impacts the level and/or function of one or more of these proteins. Because of the high neonatal lethality associated with the *Poglut2/3 DKO*, we utilized late gestation *Poglut2/3 DKOs* to characterize the effects of the mutation on FBN *in vivo*. Immunofluorescent and transmission electron microscopy revealed reduced levels and globular appearance of FBNs and elastin in the E18.5 *DKO* lung tissues. Proteomic analyses of *Poglut2/3 DKO* primary fibroblasts suggested that blocking *O-*glucose modestly decreased FBN1 secretion and significantly decreased the level of FBN1 incorporated in the ECM. Together, these findings suggest that POGLUT2/3-mediated *O-*glucosylation is required for efficient secretion and incorporation of FBNs into the ECM.

## Results

### POGLUT2/3-mediated EGF O-glucosylation is essential for mouse neonatal survival

**Figure 1:**
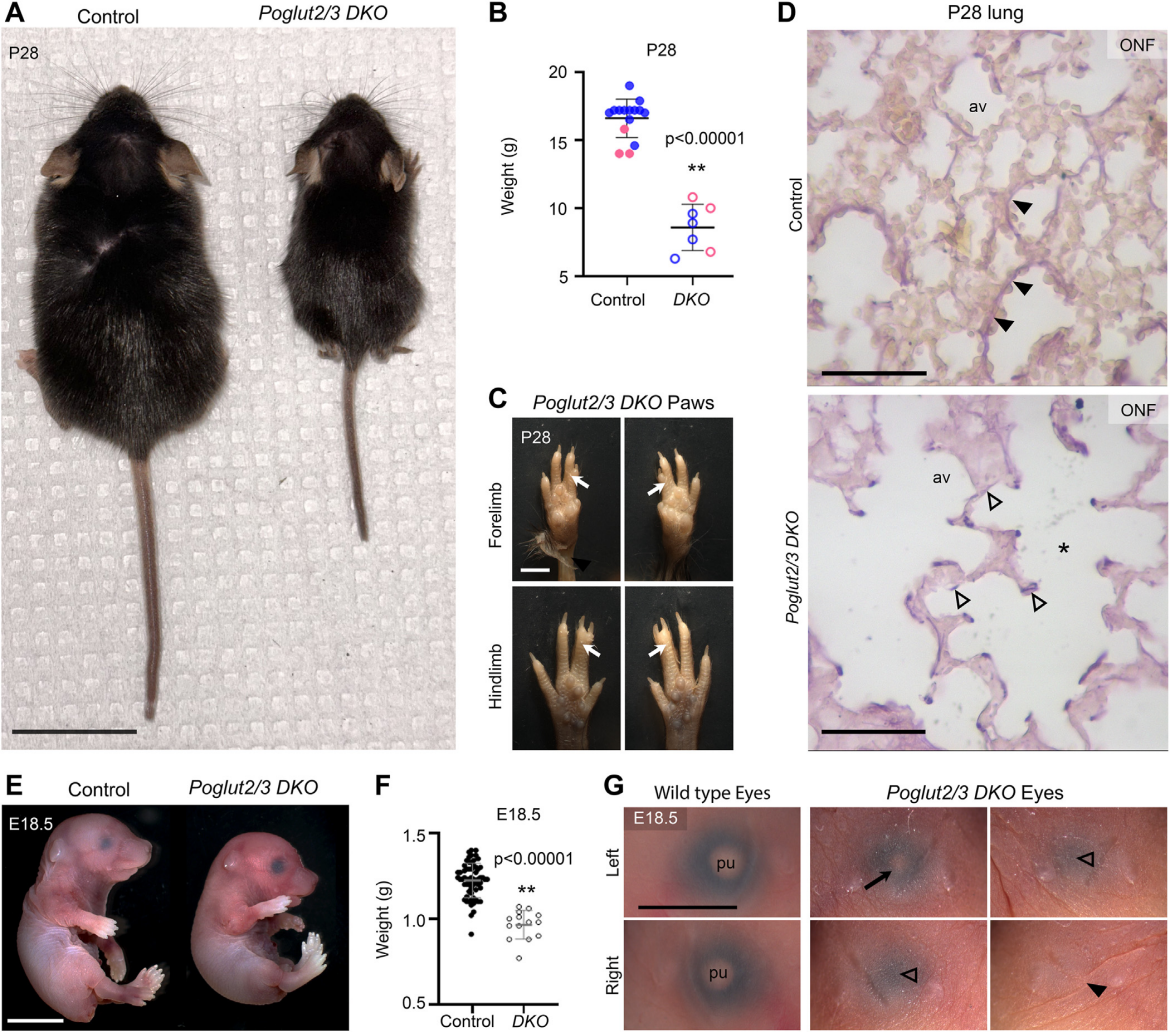
***Poglut2/3 DKOs* were runted with limb, lung, and ocular defects.***A*, size comparison of 28-days (P28) control (*left*) and *Poglut2/3 DKO* (right) and (*B*) P28 weights from control (n = 15) and *Poglut2/3 DKO* (*DKO,* n = 7) animals. The mean weight is indicated by a horizontal bar. Males and females are indicated by *blue* and *pink* color, respectively. Filled circles are controls and open circles are *DKOs*. *C*, syndactyly affected forelimb (above) and hindlimb (below) digits 2 to 3 in *Poglut2/3 DKOs* indicated by *white* arrows. *D*, comparison of postnatal day 28 lung alveolar (av) region for elastic fiber (*deep purple*) using Orcinol-New Fuchsin (ONF) stain from control (above) and *Poglut2/3 DKO* (below). In the alveolar region of *Poglut2/3 DKO*, ∗ indicates the enlarged airspace. *Closed and open arrowheads* indicate the intact and broken elastic fibers in control and *Poglut2/3 DKO,* respectively. *E*, comparison of embryonic day 18.5 (E18.5) control (*left*) and *Poglut2/3 DKO* (*right*) fetuses and (*F*) E18.5 weights from control (n = 63, filled *black* circle) and *Poglut2/3 DKO* (n = 14, open *black* circle) fetuses. *G*, E18.5 eyes in wild type with distinct pupil (pu). A range of eye phenotypes including reduced pupil (indicated by arrow), absent pupils (*open arrowheads*), and absent eye (*closed arrowheads*) was observed in E18.5 *Poglut2/3 DKO* mutants (also refer to Fig. S1*C*). The significance of weight differences was evaluated using an unpaired two-tailed *t* test; ∗∗*p* ≤ 0.01. Error bars show ± SD. Scale bars in panels A (2 cm), *E* (5 mm), *C* and *G* (2 mm), and *panels D* (50 μm).

To determine if POGLUT2/3-mediated *O*-glucose modifications on EGF repeats are required *in vivo*, we generated *Poglut2* and *Poglut3* single knockouts (*SKO*) (Tables S1 and S2, Fig. S1, *A* and *B*). Mice lacking either *Poglut2* or *Poglut3* were viable, fertile, and present at expected Mendelian ratios (Table 1). In contrast, mice lacking both *Poglut2* and *Poglut3* (*Poglut2/3 DKO*) were statistically underrepresented at weaning (Tables 1 and S3). Newborn *Poglut2/3 DKO* were viable, less active, severely runted, and usually did not survive beyond postnatal day 0 (P0), suggesting cardiopulmonary defects. Rare surviving *Poglut2/3 DKOs* were runted and had fully penetrant syndactyly between digits two and three in both the fore- and hindlimbs (Fig. 1, *A*–*C*). The lung parenchyma from rare, surviving *Poglut2/3 DKO* animals showed greatly reduced and fragmented elastic fibers with wide airspaces compared to control (Fig. 1*D*). Similar defects in elastic fibers were described for *Fbn1*^*mgR/mgR*^*, Fbn1*^*C1041G/+*^, *Ltbp4*^*−/−*^, and *Fbln5*^*−/−*^ mutants (24, 25, 26, 27). At embryonic day (E) 18.5, *Poglut2/3 DKOs* were viable and present at the expected Mendelian ratio (Table 1). The E18.5 *Poglut2/3 DKOs* were significantly smaller and had a higher incidence of eye defects (∼50%) compared to littermates (Figs. 1, *E*–*G* and S1*C*). These phenotypes were similar to mouse knockouts of *Fbn2*, which display syndactyly, and to *Fbn1* and *Ltbp1* mutants which show ocular, cardiopulmonary, and elastic fiber defects as well as early perinatal/neonatal lethality (26, 28, 29, 30, 31, 32, 33, 34, 35, 36). Together, these data suggest that the *Poglut2/3 DKO* impacted the function of FBNs and/or LTBPs.

**Table 1:** Poglut2 or Polgut3 SKO and Poglut2/3 DKO mice viability

Intercross^a^: *Poglut2 WT/KO*					Intercross^b^: *Poglut3 WT/KO*				
Age	Genotype	# Weaned	# Expected	Chi square^c^ (p)	Age	Genotype	# Weaned	# Expected	Chi square^c^ (p)
P21	*WT/WT*	18	18.75		P21	*WT/WT*	18	16.5	
	*WT/KO*	34	37.5			*WT/KO*	34	33	
	*KO/KO*	23	18.75	1.32 (0.5169)		*KO/KO*	14	16.5	0.545 (0.7613)


Intercross^d^: *Poglut2 KO; Poglut3 Het*					Intercross^d^: *Poglut2 KO; Poglut3 Het*				
Age	Genotype (*Polgut2;Poglut3*)	# Weaned	# Expected	Chi square^c^ (p)	Age	Genotype (*Polgut2;Poglut3*)	# Weaned	# Expected	Chi square^c^ (p)
P21	*KO;WT*	39	24.25		P21	*KO;WT*	31	25.25	
	*KO;Het*	50	48.5			*KO;Het*	56	50.5	
	*KO;KO*	8	24.25	19.07 (5e-5)		*KO;KO*	14	25.25	6.921 (0.03)
E18.5	*KO;WT*	22	18.5		E18.5	*KO;WT*	10	11	
	*KO;Het*	34	36.5			*KO;Het*	24	22	
	*KO;KO*	17	18.25	1.027 (0.60)		*KO;KO*	10	11	0.364 (0.83)

Het, Heterozygote; KO, Knockout; WT, Wild type.

a Animals were generated from intercrosses using C57BL/6J backcross generation N0 through N6.

b Animals were generated from intercrosses using C57BL/6J backcross generation N0 through N3.

c Chi squared with 2 degrees of freedom.

d Animals were generated from intercrosses using C57BL/6J backcross generation N0 through N2.

### POGLUT2/3-mediated O-glucosylation is lost on secreted extracellular matrix proteins in the Poglut2/3 DKO

**Figure 2:**
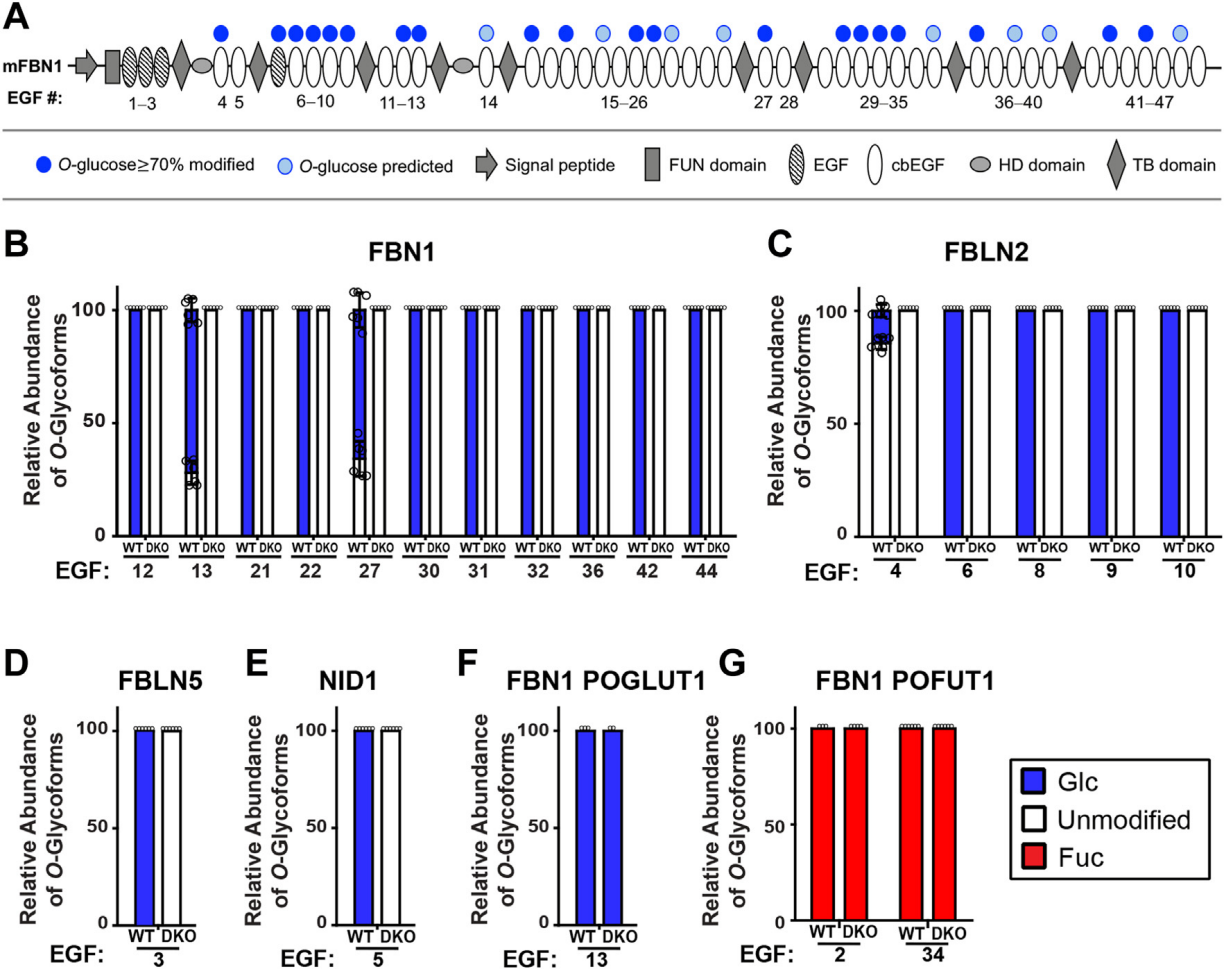
**POGLUT2 andPOGLUT3modifications were lost in *Poglut2/3 DKO* mice.***A*, domain map of mouse fibrillin1 (mFBN1) with numbered EGFs. POGLUT2/3 *O-*glucosylated EGFs as determined in (*B*) and (Fig. S2) are indicated by *blue* circles and EGFs predicted to be *O-*glucosylated are indicated by light blue circles. *B*–*E*, relative abundance of POGLUT2/3-mediated *O*-glucosylation (Glc, *blue*) on EGFs from endogenous (*B*) FBN1, (*C*) FBLN2 (fibulin 2), (*D*) FBLN5 (fibulin 5), and (*E*) NID1 (nidogen) secreted from wild type (WT) or *Poglut2/3 DKO (DKO)* E18.5 lung fibroblasts. *F*, relative abundance of *O*-glucose (*blue*) added by POGLUT1 on EGF13 of endogenous FBN1 secreted from *WT* or *DKO* lung fibroblasts. *G*, relative abundance of *O*-fucose (Fuc, *red*) added by POFUT1 on EGF2 and EGF34 of endogenous FBN1 secreted from WT or *DKO* lung fibroblasts. The relative abundance of *O-*glycoforms was calculated using the area under the curve from extracted ion chromatograms and was plotted as a percentage of the total abundance for each peptide. MS/MS spectra and Extracted Ion Chromatograms (EICs) for peptides quantified can be found in Dataset S1. Masses for these peptides are in Excel file 1. Averages were taken from n = 3 biological replicates. The number of technical replicates (media collected from fibroblast cultures) differed. *DKO* Animal 1, three technical replicates; *DKO* Animal 2, two technical replicates; *DKO* animal 3, one technical replicate. For WT animals, two technical replicates per animal were analyzed. Up to six technical replicates were reported per EGF depending on sequence coverage. Panel E had only two biological replicates. Error bars show ± SD.

We used mass spectral glycoproteomic analysis to confirm that the *Poglut2/3 DKO* blocked *O-*glucosylation of EGFs*.* In *Poglut2* and *Poglut3 SKO* dermal fibroblast cultures, *O-*glucosylation on FBN1 EGFs remained largely the same (Fig. S2*A*), except for six FBN1 EGFs that completely lost or significantly reduced *O-*glucosylation in *Poglut2* or *Poglut3 SKO* fibroblast cultures (Fig. S2*B*). This observation suggests that the POGLUT2 and POGLUT3 enzymes are largely functionally redundant but have limited site-specificity. In contrast, *O-*glucosylation was eliminated on FBN1 EGFs in *Poglut2/3 DKO* E18.5 lung fibroblast cultures (Fig. 2*A*, Dataset S1, and Excel file 1), demonstrating that no other enzymes modify these sites. Additionally, we identified POGLUT2/3-mediated *O*-glucosylation on fibulin-2, fibulin-5, and nidogen-1 from wild-type fibroblasts that were eliminated in *Poglut2/3 DKOs* (Fig. 2, *B*–*D*, Dataset S1 and Excel file 1). Neither β-hydroxylation, which occurs within the POGLUT2/3 consensus (14), nor *O*-glycosylation at other EGF locations mediated by POGLUT1 or POFUT1 were affected by loss of POGLUT2/3 (Figs. 2, *E* and *F* and S2, *C*–*F*, Dataset S1 and Excel file 1).

### *Poglut2* and *Poglut3* are widely expressed in E18.5 lung and are essential for lung tissue organization

**Figure 3:**
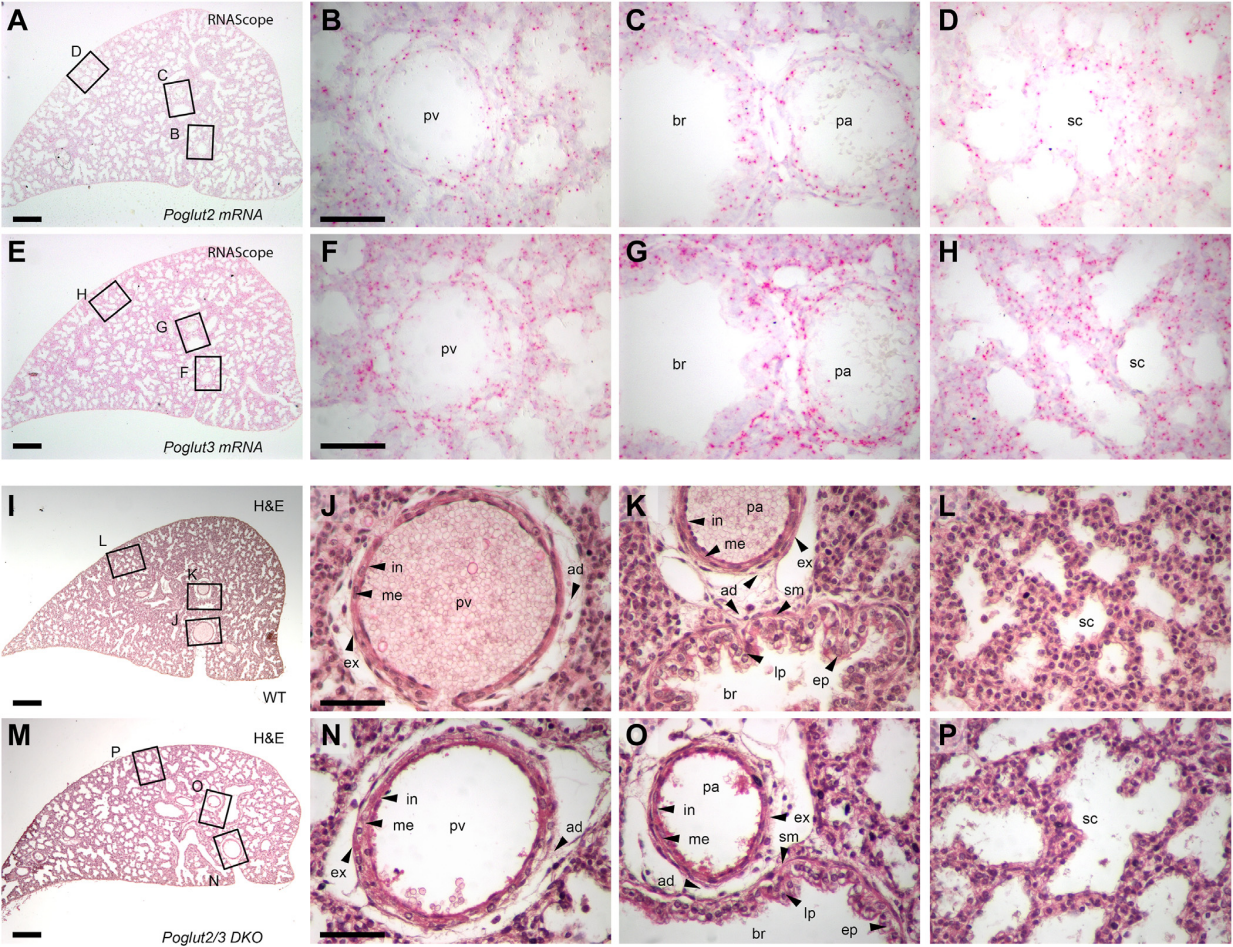
***Poglut2* and*Poglut3*were expressed in and essential for lung tissue organization.***A*–*H*, RNAScope analysis of *Poglut2* (*A*–*D*) and *Poglut3* (*E*–*H*) mRNA distribution in lungs at E18.5. *Red dots* or blobs represent detection of *Poglut2* or *Poglut3* mRNA (controls for RNAScope and qRT-PCR analyses of RNA levels are shown in Fig. S3). Rectangles in *panel A* and *E* indicate lung regions magnified in *panels B*, *C*, *D* and *F*, *G*, *H* respectively. *Poglut2* and *Poglut3* transcripts were present in all layers of the lung pulmonary vein (pv), pulmonary artery (pa) and bronchiole (br) (*B*, *C*, *F*, *G*) and saccule (sc) (*D*, *H*) regions. *I–P*, hematoxylin and eosin (H&E) staining of lung sections from E18.5 wild type (WT) (*I*–*L*) and *Poglut2/3 DKO* (*M–P*). *I* and *M*, lower magnification of H&E-stained lung section from WT (*I*) and *Poglut2/3 DKO* (*M*). *Rectangles* in *panels I* and *M* indicate lung regions magnified in (*J*), (*K*), (*L*), (*N*), (*O*), and (*P*), respectively. The regions equivalent to *I* and *M* were imaged at high magnification in all the confocal imaging in the subsequent figures. *J* and *K*, Wild-type lung pv and pa showing a well-defined structure of inner intima (in), media (me), outer externa (ex) and adventitia (ad), and br showing distinct mucosal layer with epithelial (ep) folds and lamina propria (lp) and smooth muscle (sm) and adventitia (ad) layers. *L*, wild-type lung sc region with primary septa forming air spaces. *N* and *O*, *Poglut2/3 DKO* lung pv and pa showing poorly arranged intima (in), media (me), externa (ex) and adventitia (ad) and br showing mucosal layer with flatter epithelial (ep) folds and reduced lamina propria (lp). *P*, *Poglut2/3 DKO* lung sc region with primary septa forming air spaces. Scale bars: *Panels A*, *E*, *I* and M 200 μm and panels *B*–*D*, *F*–*H*, *J*–*L* and *N–P* (50 μm, represented by scale bar in panel *B*, *F*, *J*, and *N*).

To begin to assess the impact of the *Poglut2/3 DKO* on substrates, we analyzed the late gestation lung at E18.5. At this time point, the lungs are at the saccule stage with relatively mature blood vessels and bronchioles and ongoing saccule development (37, 38, 39). We detected *Poglut2* and *Poglut3* transcripts throughout the lung epithelia, mesenchyme, and vasculature (Figs. 3, *A*–*H*, S3, *A*–*D*), with higher levels of *Poglut3* mRNA detected using quantitative RT-PCR (Fig. S3*E*). Given the overlapping patterns of *Poglut2* and *Poglut3* gene expression and the persistence of *O-*glucosylated FBN1 EGFs in the single knockouts (Fig. S2), the lack of phenotype in the *Poglut2* and *Poglut3* single gene knockouts is likely due to redundancy of the POGLUT2/3 enzymes.

In hematoxylin and eosin-stained E18.5 lung sections, we observed abnormalities in the organization of cells in the *Poglut2/3 DKOs* blood vessels, bronchioles, and saccules compared to controls (Fig. 3, *I*–*P*). The pre-acinar blood vessel (pulmonary vein, pv, and pulmonary artery, pa) walls were thicker, similar to the Marfan mouse model (*Fbn1 mgR/mgR*) (40), with poorly arranged endothelium, loose and abnormal media, and aberrant internal and external layers compared to wild-type lung blood vessel (Fig. 3, *J* and *K*, *N* and *O*). In addition, the epithelial folds of the *Poglut2/3 DKO* bronchioles were flattened with reduced lamina propria (Fig. 3, *K* and *O*). The saccule airspaces appeared enlarged in *Poglut2/3 DKOs* compared to control (Fig. 3, *L* and *P*), which could reflect either regional sampling differences or a defect in late gestation terminal bronchiole branching or saccule morphogenesis. Airspace enlargement was also reported for *Fbn1*^*mgR/mgR*^ and *FBN1*^*C1041G/+*^ mouse models (25, 26, 41). The shared structural abnormalities raise the possibility that the *Poglut2/3 DKO* impacted fibrillin levels.

### FBN1, FBN2, and elastin levels are reduced and disorganized in E18.5 *Poglut2/3* DKO lung

**Figure 4:**
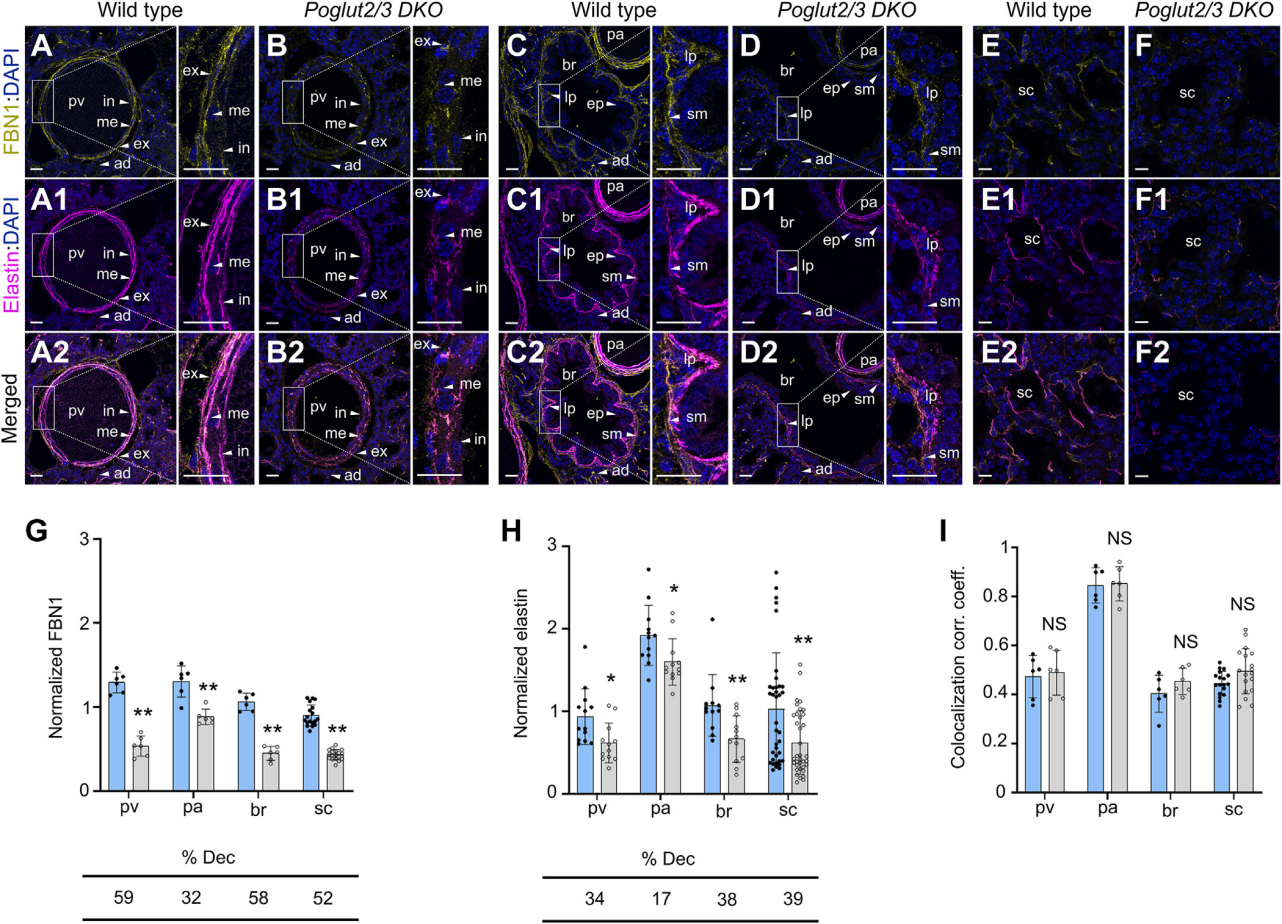
**FBN1 and elastin levels were decreased but colocalized in E18.5 *Poglut2/3 DKO* lung.** Representative maximum projection images comparing FBN1 (*yellow*) *A*–*F*, immunolocalization, elastin (*magenta*) detection (A1-F1) using alexafluor 633 (AF633) with DAPI counterstaining (*blue*), and FBN1 and elastin colocalization (A2-F2) in E18.5 lung sections. *A, A1 and inset*, in wild-type pulmonary vein (pv), FBN1 and elastin localized to distinct layers of the internal (in) and external (ex) elastic lamina with diffuse FBN1staining also observed in the media (me) and adventitia (ad) layers. (*B*, B1 and *inset*) In *Poglut2/3 DKO* pv, FBN1and elastin showed fragmented and discontinuous staining. (*C*, C1 and *inset*) In wild-type pulmonary artery (pa), FBN1 and elastin localized to distinct layers of the internal (in) and external (ex) elastic lamina with strong FBN1 staining in the media (me). *C, C1, and inset*, In wild-type bronchiole (br), strong FBN1 and elastin staining were detected in the basement membrane underlying the characteristically folded epithelial layer (ep) and smooth muscle (sm) layers with reduced staining in the lamina propria (lp). *D, D1*, In *Poglut2/3 DKO* pa, FBN1, and elastin showed reduced staining. *D, D1, and inset*, In *Poglut2/3 DKO* br, significantly reduced and thinner FBN1 and elastin staining was observed. *E, E1*, in wild-type lung saccules (sc), FBN1, and elastin localized to the matrix underlying the alveolar epithelial cells. *F, F1*, in the *Poglut2/3 DKO* sc, FBN1, and elastin showed punctate and discontinuous staining. (A2-F2) Merged channels of maximum projection images of FBN1 and elastin AF6333 in E18.5 lung pv, pa, br, and sc. White rectangles indicate the digitally enlarged regions. *G*–*I*, quantification of FBN1 (*G*) and elastin (AF633) (*H*) signals normalized to DAPI and estimation of colocalization correlation (corr.) coefficient (coeff.) (*I*) in the pv, pa, br, and sc regions. Elastin signals graphed in panel H are pooled from all the FBN1/elastin and FBN2/elastin-stained wild type and *Poglut2/3 DKO* sections, and the graph in panel H is reused in Figure 5*H*. Regions of interest (ROIs) are defined in Fig. S4 and reuse images from *panels A*–*D* for reference. Percent decreased (% dec) from wild type indicated below the graph. Data from wild type (*blue* column with *solid black circles*) and *Poglut2/3 DKO* (*gray* column with o*pen black circles*) were evaluated for statistical significance using unpaired, two-tailed *t* test: ∗*p* 0.05, ∗∗*p* 0.01 and NS, not significant. Error bars show ± SD. Scale bar panels A-D2 and magnified regions in *A*-D2: 20 μm. Scale bars in *E*-F2: 10 μm. Images were obtained from three embryos per genotype, 2 to 3 sections per embryo, and for pv, pa and br regions one field per section and for sc region 2 to 3 fields per sections. Lung sections used for immunostaining and analysis were adjacent to H&E-stained sections (Fig. 3) which showed pulmonary veins, bronchiole and pulmonary artery.

**Figure 5:**
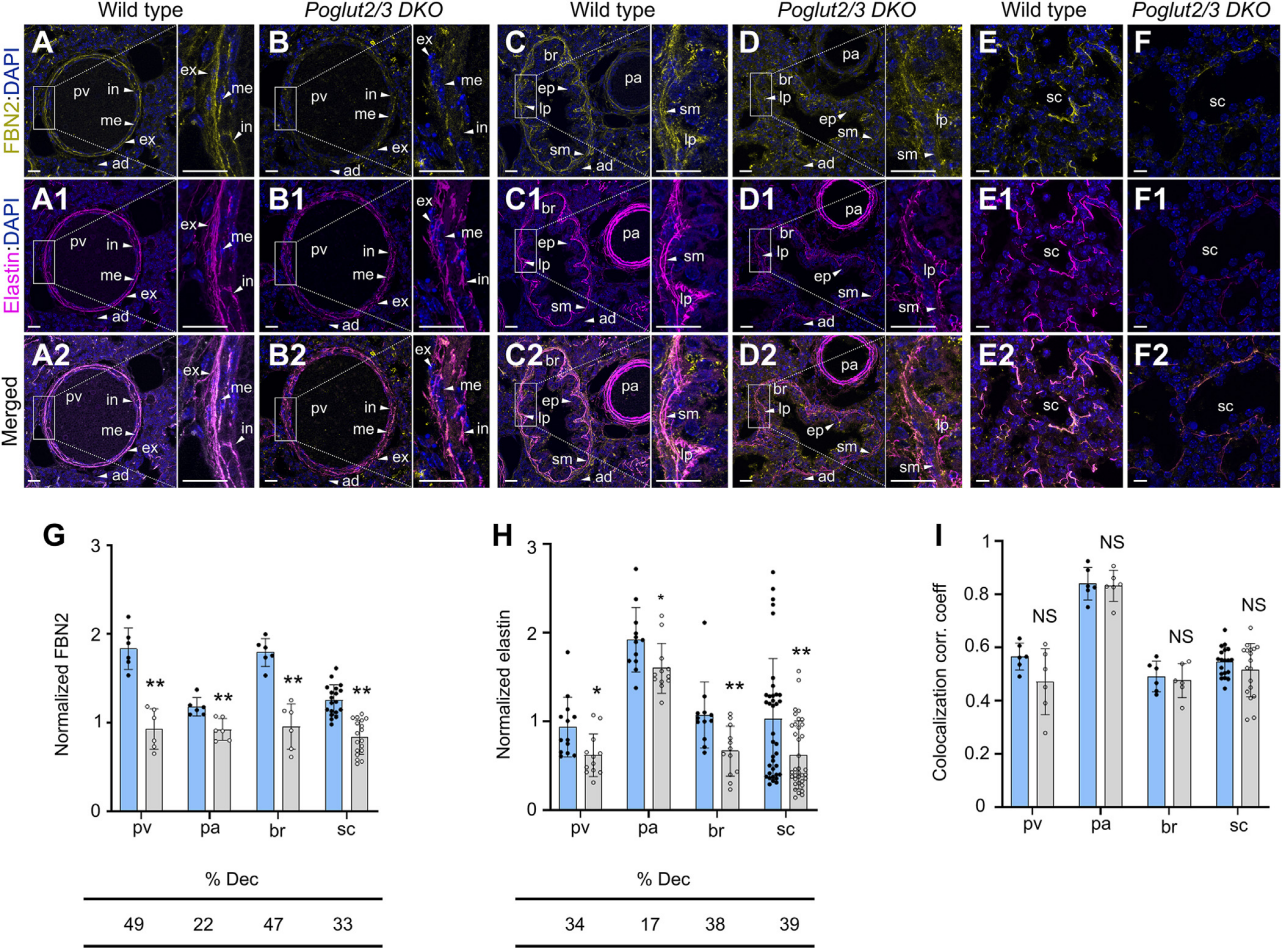
**FBN2 and elastin levels were decreased but colocalized in E18.5 *Poglut2/3 DKO* lung.** Representative maximum projection images comparing FBN2 (*yellow*) (*A*–*F*) immunolocalization, elastin (*magenta*) detection (A1-F1) using Alexa Fluor 633 (AF633) with DAPI counterstaining (*blue*), and FBN2 and elastin colocalization (A2-F2) in E18.5 lung sections. *A, A1, C, C1 and inset*, In wild-type pulmonary vein (pv) and pulmonary artery (pa), FBN2 and elastin localized to distinct layers of the internal (in) and external (ex) elastic lamina with diffuse FBN2 staining also observed in the media (me) and adventitia (ad) layers. *B, B1, and inset*, in *Poglut2/3 DKO* pv, FBN2, and elastin showed fragmented and discontinuous staining. *D, D1, and inset*, in *Poglut2/3 DKO* pa, FBN2 and elastin showed reduced staining. *C, C1 and inset*, in wild-type bronchiole (br), strong FBN2 and elastin staining were detected in the basement membrane underlying the characteristically folded epithelial layer (ep) and smooth muscle (sm) layers with reduced staining in the lamina propria (lp). *D, D1, and inset*, In *Poglut2/3 DKO br,* significantly reduced and thinner FBN2 and elastin staining was observed. (*E*, E1) In wild-type lung saccules (sc), FBN2 and elastin localized to the matrix underlying the alveolar epithelial cells. (*F*, F1) In the *Poglut2/3 DKO* sc, FBN2 and elastin showed punctate and discontinuous staining. (A2-F2) Merged channels of maximum projection images of FBN2 and elastin AF6333 in E18.5 lung pv, pa, br, and sc. White *rectangles* indicate the digitally enlarged regions. *G*–*I*, quantification of FBN2 (*G*) and elastin (AF633) (*H*) signals normalized to DAPI and estimation of colocalization correlation (corr.) coefficient (coeff.) (*I*) in the pv, pa, br, and sc regions (ROI defined in Fig. S4). The graph in *panel H* is reused for reference from Figure 4. Percent decreased (% dec) from wild type indicated below the graph. Data from wild type (*blue* column with *solid black circles*) and *Poglut2/3 DKO* (*gray* column with *open black circles*) were evaluated for statistical significance using unpaired, two-tailed *t* test: ∗*p* 0.05,∗∗*p* 0.01 and NS, not significant. Error bars show ± SD. Scale bar *panels A*-D2 and magnified regions in *A*-D2: 20 μm. Scale bars in *panels E*-F2: 10 μm. Images were obtained from three embryos per genotype, 2 to 3 sections per embryo, and for pv, pa, and br regions one field per section and for sc region 2 to 3 fields per section. Lung sections used for immunostaining and analysis were adjacent to H&E-stained sections (Fig. 3) which showed pulmonary veins, bronchiole and pulmonary artery.

We used the late gestation lung from *Poglut2/3 DKOs* at E18.5 to analyze the *in vivo* effects on FBN and elastin levels. In control lungs, FBN1 and FBN2 localized in the pre-acinar pulmonary artery and veins, bronchiole, and primary septa of developing saccules (Figs 4, *A*, *C* and *E*, 5, *A*, *C* and *E*, S4, *A*–*H* region of interest (ROI) defined, Figs. S4 and S5). Single knockout of either *Poglut2* or *Poglut3* only slightly reduced FBN1 and FBN2 levels (Figs. S4 and S5). In contrast, FBN1 and FBN2 levels were significantly reduced in all regions of the *Poglut2/3 DKO* (Figs. 4, *B*, *D*, *F* and *G* and 5, *B*, *D*, *F* and *G*). Moreover, FBN1 and FBN2 staining in the internal and external lamina of the pulmonary artery were disrupted and punctate in the *Poglut2/3 DKO* (Figs. 4*B* and 5*B*, figures in the insets). In addition, FBN staining lacked the characteristic scalloped organization in the bronchioles was non-contiguous and appeared thinner in the saccule regions of the *Polgut2/3 DKO* (Figs. 4, *D* and *F* and 5, *D* and *F*, figures in the insets).

FBN microfibrils provide the foundation for elastic fiber formation (29, 42, 43)*.* Consistent with this role, we observed a decrease in elastic fibers (detected by Alexa fluor-633) in the *Poglut2/3 DKO* pulmonary blood vessels (pv, 34% and pa, 17%), bronchiole (38%), and saccules (39%) compared to controls or *SKOs* (Fig. 4A1-F1, A2-F2, H, Fig. 5A1-F1, A2-F2, H, and Figs. S4 and S5). Elastin staining in the *Poglut2/3 DKOs* was fragmented similar to staining reported for *Fbn1* mutants (31, 44). The colocalization correlation coefficient of FBN1-elastin was comparable between wild type and *Poglut2/3 DKOs* (Figs. 4*I* and 5*I*), suggesting that elastin deposition was not impaired. Rather, the lower levels and fragmented appearance of elastin in *Poglut2/3 DKOs* were likely secondary to the sparse pattern of unglucosylated FBN1.

**Figure 6:**
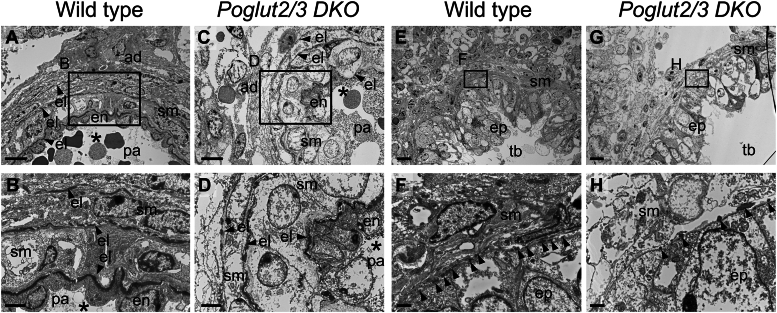
**Fragmented elastic lamella and reduced elastin in the pulmonary artery and bronchiole of *Poglut2/3 DKO* lung.***A*–*H*, transmission electron micrograph of pulmonary artery (*A*–*D*) and bronchiole (*E*–*H*) from wild type (*A*, *B*, *E*, *F*) and *Poglut2/3 DKO* (*C*, *D*, *G*, *H*) lung. Asterisk (∗) denotes artery lumen. *A* and *B*, In wild type pulmonary artery (pa) and elastic lamella (el), indicated by *arrowheads*, were largely intact and distinctly visible. The internal elastic lamina separating the endothelium (en) and smooth muscle layer (sm) was largely intact. The middle elastic lamina separating smooth muscle layers and the external elastic lamina separating smooth muscle and adventitia (ad) layers had occasional breaks. *C* and *D*, in the *Poglut2/3 DKO*, all elastic lamella were fragmented and with sparse elastic fibers in the outer layers. *E* and *F*, in wild-type bronchiole (br) elastin deposits were localized (indicated by arrowheads) in the region between the epithelium (ep) and smooth muscle (sm) layer. *G* and *H*, in *Poglut2/3 DKO* elastin deposits were greatly reduced (indicated by *arrowheads*). Additional neighboring images from these wild type and *Poglut2/3 DKO* sections are shown in Fig. S6 and indicate the position of *panels A*, *C*, *E*, and *G* in the sections. *Rectangles* in *A*, *C*, *E*, and *G* indicate the magnified regions in *B*, *D*, *F*, and *H*, respectively. Scale bars: *panels A* and *C* (6 μm), *E* and *G* (10 μm), *B* and *D* (2 μm) and *F* and *H* (1 μm).

Transmission electron microscopy (TEM) imaging of *Poglut2/3 DKO* E18.5 lung confirmed defects in the elastic fibers of the blood vessels and bronchiole (Figs. 6 and S6). In wild-type pulmonary artery, the internal, medial, and external elastic layers were distinct (Figs. 6, *A* and *B*, and S6, A-A4). The internal elastic lamina (next to endothelium) was largely intact and continuous with few breaks (Figs. 6*B*, and S6, A1–A4), while the middle and external elastic laminae were thinner with a greater number of breaks (Fig. 6*B*). In contrast, the elastic laminae were sparse in the *Poglut2/3 DKO* with extensive fragmentation in the internal elastic lamina and nearly punctate in the middle and external laminae (Figs. 6, *C* and *D*, and S6, B-B4). The ultrastructure of wild-type bronchiole showed intermittent elastin deposition in the area at the base of the folded epithelium (Figs. 6, *E* and *F*, and Fig. S6, C, C1, D-D2). In contrast, elastin deposition in the *Poglut2/3 DKO* was more dispersed in the bronchiole (Figs. 6, *G* and *H*, S6, E, E1, and F-F2). In the wild type, the smooth muscle cells were elongated in both the pulmonary artery and the bronchiole, whereas they appeared round in the *Poglut2/3 DKO* (Figs. 6, *C*, *D*, *G* and *H*, and S6). The altered characteristics of the *Poglut2/3 DKO* smooth muscle cells are reminiscent of Marfan syndrome, where rounded smooth muscle cells in the aorta are attributed to elastic fiber fragmentation observed by TEM (45, 46).

**Figure 7:**
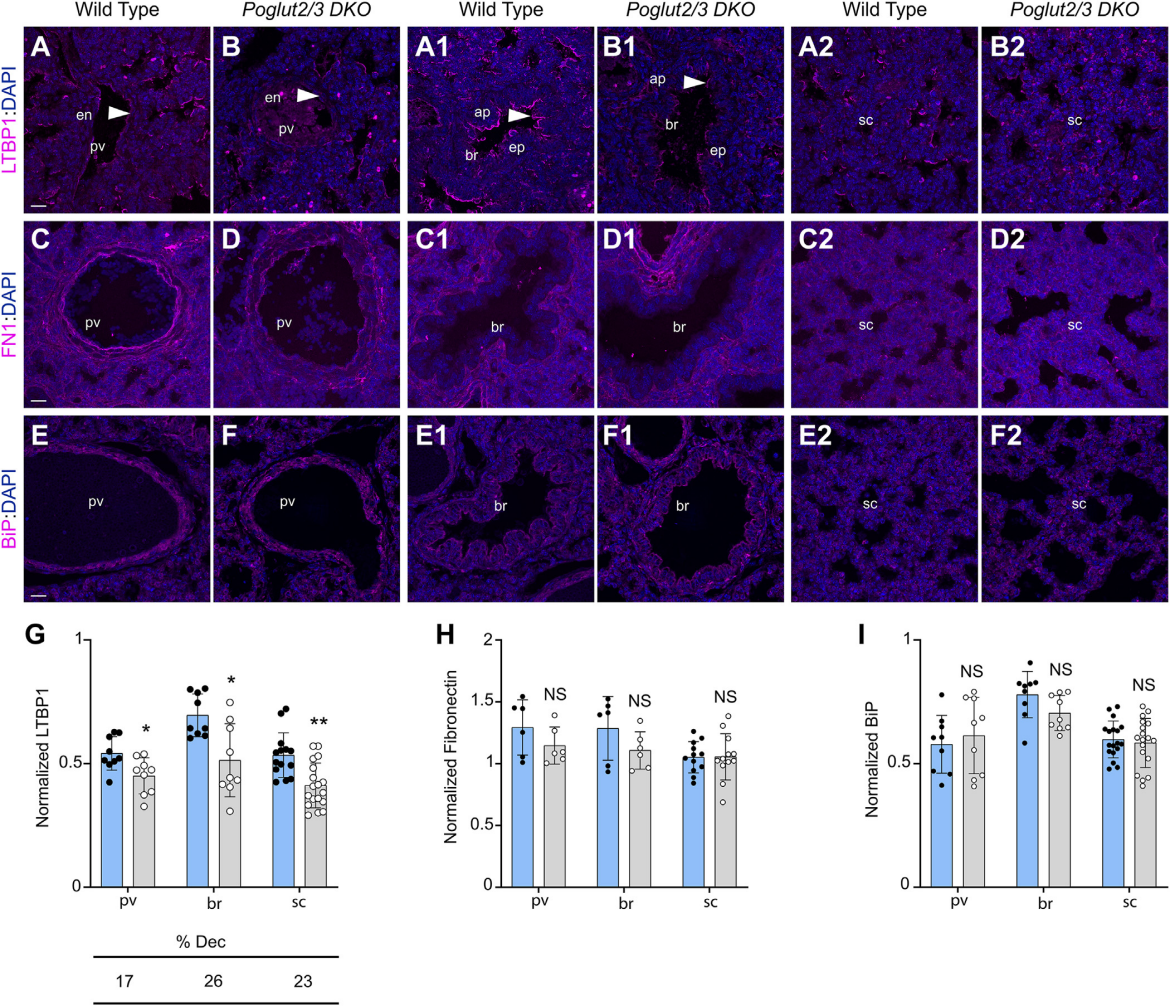
**Loss of *Poglut2/3* slightly decreased LTBP1 levels in E18.5 lung.***A-B2*, comparison of representative maximum projection images of LTBP1 (*magenta*) localization and counterstained with DAPI (*blue*) in E18.5 lung pulmonary vein (pv, *A* and *B*), bronchiole (br; A1-B1), and saccule (sc; A2-B2) regions from wild type and *Poglut2/3 DKO*. (A-A2) In wild type lung, strong LTBP1 signal was observed in the pv endothelium (en), the tb apical (ap) epithelial surface (ep), and in the interstitium of the sc region. *B-B2*, in *Poglut2/3 DKO* lung LTBP signal was similarly localized in pv, br, and sc, but the signal intensity was reduced. *C-D2*, comparison of representative maximum projection images of fibronectin (*magenta*) localization and counterstained with DAPI (*blue*) showed similar staining in E18.5 lung blood vessel (pv; *C* and *D*), bronchiole (br; C1-D1) and saccules (sc; C2-D2) from wild type (C-C2) and *Poglut2/3 DKO* (D-D2). *E-F2*, comparison of representative maximum projection images of BiP (*magenta*) localization and counterstained with DAPI (*blue*) showed similar staining and localization in E18.5 lung blood vessel (pv; *E* and *F*), bronchiole (br; E1-LF) and saccule (sc; E2-F2) from wild type (E-E2) and *Poglut2/3 DKO* (F-F2). *G*–*I*, quantification of LTBP1 (*G*), fibronectin (*H*), and BiP (*I*) immunofluorescence signals in the pv, br, and sc (ROIs defined in Fig. S4) from wild type and *Poglut2/3 DKO*. Immunofluorescence signals from individual images was normalized with DAPI signals from the same image. Data from wild type (*blue column* with s*olid black circles*) and *Poglut2/3 DKO* (*gray column* with *open circle*) were evaluated for statistical significance using unpaired, two-tailed *t* test: ∗*p* < 0.05, ∗∗*p* < 0.01 and NS, not significant. Error bars show ± SD. Scale bars: all panels 20 μm. Images were obtained from three embryos per genotype, 2 to 3 sections per embryo, and for pv and br regions one field per section and for sc region 2 to 3 fields per sections.

By comparison, levels of LTBP1 (a POGLUT2/3 substrate with 11 out of 18 EGFs *O-*glucosylated), which facilitates fibrillin microfibril assembly (47, 48, 49), were unchanged in *Poglut2* or *Poglut3 SKO*s lung sections and slightly reduced in the *Poglut2/3 DKO* (Figs. 7, *A*-B2, *G*, and S7, A-C2, *J*). In addition, levels of fibronectin, which is required for FBN microfibrils (50, 51), were unaffected in the *Poglut2/3 DKO* and *SKOs* (Figs. 7, *C*-D2, H, and S7, D-F2, *K*). BiP levels were not elevated in the *Poglut2/3 DKO* lung tissues, suggesting that loss of *O-*glucose did not impair the secretion of FBN enough to activate the unfolded protein response (Figs. 7, *E*-F2, *I*, and S7, G-I2, *L*). Combined, these observations suggest that FBN1 and FBN2 were sensitive to loss of POGLUT2/3 mediated *O-*glucosylation, leading to reduced levels and punctate appearance of microfibrils and elastin deposition which could contribute to developmental deficiencies in *Poglut2/3 DKO* lung.

### Loss of *O*-glucose reduces FBN secretion and incorporation into the ECM

**Figure 8:**
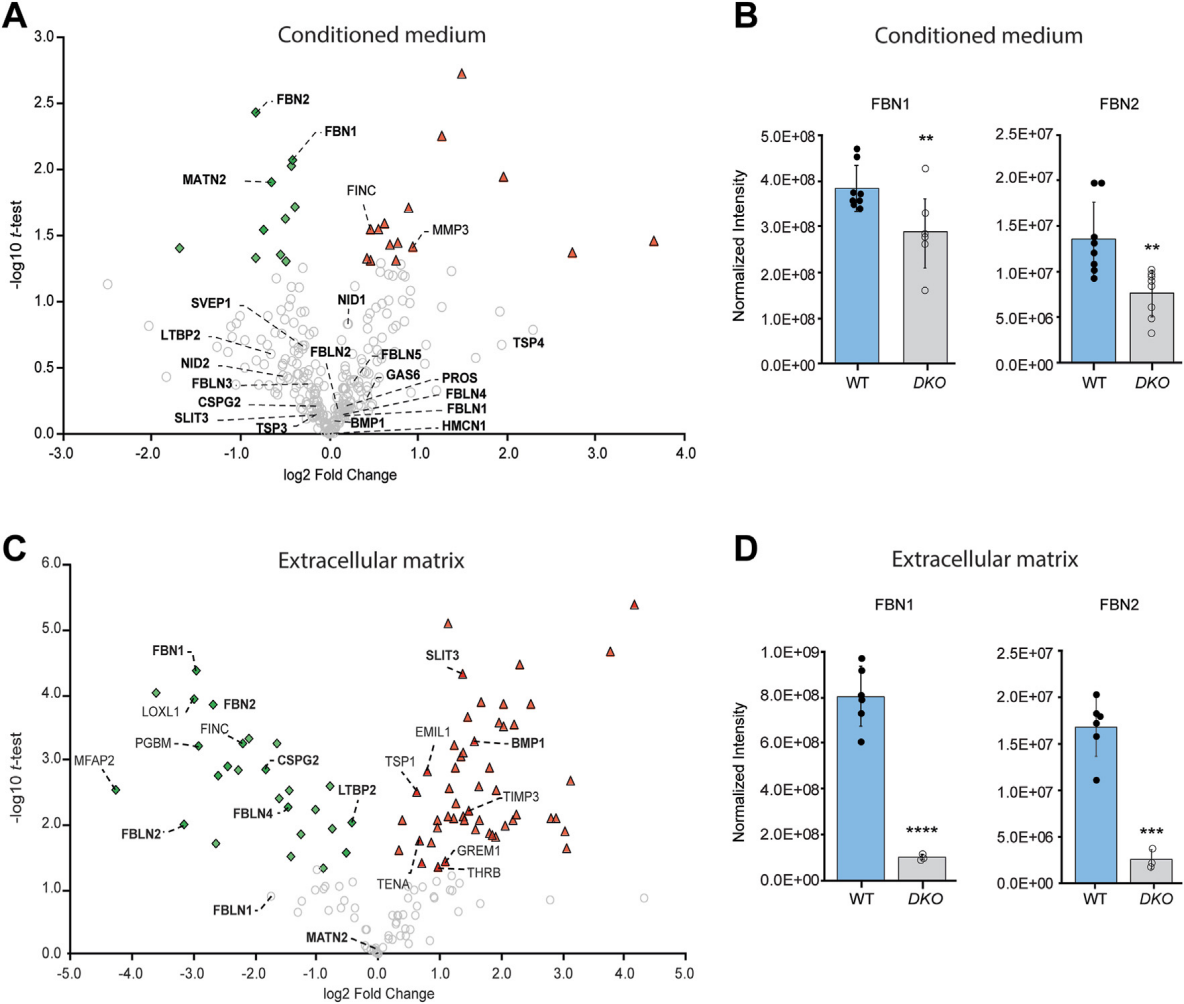
**Microfibril-incorporated levels of FBN are reduced more than secreted levels in *Poglut2/3 DKO* dermal fibroblasts at E18.5.** Proteins present in WT and *Poglut2/3 DKO* dermal fibroblast culture medium and ECM were detected and quantified by mass spectrometry. A complete list of all identified extracellular proteins (and corresponding protein abbreviations) is in Excel files 2 and 3. *A* and *C*, volcano plot displays log2 fold changes in abundance of extracellular proteins in *Poglut2/3 DKO* (*DKO*) dermal fibroblasts (*A*) medium or (*C*) deposited extracellular matrix compared to wild type (WT) relative to the -log10 *t* test*.* POGLUT2/3 substrates are bolded. *Green*-filled diamonds identify proteins with statistically significant reduced abundance (*p* ≤ 0.05); *gray open circles,* proteins with no statistically significant change in abundance; red filled triangles, proteins with statistically significant increased abundance. *B* and *D*, using the same data sets used to generate *panels A* and *C*, the abundance of FBN1 and FBN2 in dermal fibroblast (*B*) media and (*D*) deposited extracellular matrix was compared between *DKO* and WT. For *panels A*–*D*, replicates were normalized to the sample with the highest total protein intensity. WT n = 8 (4 biological replicates run in duplicate for media) and n = 6 (3 biological replicates run in duplicate for deposited extracellular matrix). *Poglut2/3 DKO* n = 8 (4 biological replicates run in duplicate for media) and n = 3 (2 biological replicates, one run in duplicate for deposited extracellular matrix). A two-tailed *t* test was used to calculate statistical significance, ∗∗*p* ≤ 0.01, ∗∗∗∗*p* < 0.0001. All *p* values are reported in Excel files 2 and 3. Error bars show ± SD.

In HEK293T cells, loss of either *POGLUT2* or *POGLUT3* moderately reduces the secretion of an overexpressed FBN1 fragment, with a 75% reduction seen in the *POGLUT2/3 DKO* cell line (14). For this reason, we hypothesized that a similar defect in the secretion of FBN and/or other POGLUT2/3 substrates contributed to the *Poglut2/3 DKO* phenotypes. Alternatively, if unmodified substrates were secreted at appreciable levels and the *O-*linked glucose was important for their extracellular function, then loss of POGLUT2/3 mediated *O-*glucosylation could lead to defects in microfibril incorporation and/or function. To begin to distinguish these possibilities, we performed mass spectral analyses to quantify the abundance of POGLUT2/3 substrates using Proteome Discoverer (v2.5) (52, 53). For these studies, we quantified peptide levels in a conditioned medium and deposited ECM produced by E18.5 control and *Poglut2/3 DKO* dermal fibroblasts (Fig. 8, Excel files 2 and 3). FBN1 and -2 levels were reduced by approximately 30 and 50 percent, respectively, in the conditioned medium of *Poglut2/3 DKO* fibroblasts compared to controls (Fig. 8, *A* and *B*, Excel file 2). By comparison, levels of other identified POGLUT2/3 substrates in the culture medium, including FBLNs 2, -3, and -5, Hemicentin-1, SVEP1, Nidogens 1 to 2, and LTBP 2, were unchanged compared to controls (Fig. 8*A*, Excel file 2).

In marked contrast to the conditioned medium, levels of FBN1 and -2 in the ECM were reduced 85 percent in the *Poglut2/3 DKO* compared to controls (Fig. 8, *C* and *D*, Excel file 3) and is similar to the reduction observed in lung tissues (Fig. 4). This outcome suggests that a major impact of the *Poglut2/3 DKO* was at the level of microfibril assembly and/or stability with lesser effects due to impaired secretion. The ECM levels of other POGLUT2/3 substrates such as FBLN2 and FBLN4, CSPG2, and LTBP2 were also reduced in the ECM (Fig. 8*C*, Excel file 3). Since the levels of these proteins were unchanged in the conditioned medium and these proteins bind to extracellular FBN, the observed changes could be secondary to the lowered ECM-levels of FBNs. The reduced level of non-POGLUT2/3 substrate protein MFAP2 (also called MAGP1) in *Poglut2/3 DKO* (Fig. 8*C*) could occur secondarily to the reduced level of FBN1. Elevated levels of two POGLUT2/3 substrates, BMP1 and SLIT3, and other non-POGLUT2/3 substrates such as EMILIN1 (Fig. 8*C*, Excel file 3) could arise from altered characteristics in the *Poglut2/3* mutant dermal fibroblasts, increased binding to ECM components in the absence of *O-*glucose modification or alternatively could be elevated as a compensatory response to fibrillin or elastic fiber defects.

## Discussion

FBN microfibrils afford tissues such as blood vessels, lungs, joints, and skin with tensile strength and flexibility in part by acting as a template for elastin deposition (17, 54). FBN microfibrils also provide a platform for the binding of proteins such as LTBPs, fibulins, and A Disintegrin And Metalloproteinase with ThromboSpondin motifs family members (ADAMTS/TSLs) that regulate developmental signaling and the ECM environment (8, 29). Mutations in FBNs and/or their associated proteins are responsible for connective tissue disorders that are associated with microfibril and elastic fiber fragmentation and dysregulated TGF-β signaling affecting skeletal, ocular, cardiac, and respiratory systems (11, 23, 29, 44, 55). The physiological importance of FBNs has been studied for decades, yet nothing was known about how *O-*glucose impacts their function *in vivo*.

Here, using *Poglut2* and *Poglut3* mouse knockout models we demonstrated that POGLUT2/3-mediated *O*-glucosylation was critical for viability and for efficient secretion and ECM levels of FBNs in lung tissues. Our mass-spectral analyses of primary fibroblasts demonstrated that POGLUT2/3 were responsible for adding *O-*glucose between Cysteines three and four of FBN EGFs in these cells, and also highlighted the presence of these modifications on EGFs of additional ECM proteins (Fig. 2). These analyses also demonstrated that the FBNs, with their high number of modified EGFs, were particularly sensitive to loss of *O-*glucose compared to other POGLUT2/3 substrates with fewer modified EGFs (Fig. 8). Reduced levels or altered function of secreted, unglucosylated FBNs likely contributes to runting, syndactyly, ocular, limb, and lung phenotypes in *Poglut2/3 DKOs.* Consistent with this prediction, these abnormalities are also observed in mouse *Fbn1*^*C1041G/+*^*, FBN1*^*mgR/mgR*^*, or Fbn2*^*−/−*^ mutants (26, 29, 30, 31, 36, 54, 56). In addition, lung abnormalities are also reported in mice bearing mutations in genes encoding elastin as well as proteins that interact with elastin or modulate FBN assembly/turnover including fibulin-4, fibulin-5 and LTBP4 (3, 24, 27, 34, 57, 58, 59, 60), we cannot exclude the possibility that altered function of these unglycosylated substrates in *Poglut2/3 DKOs* also impairs functions of FBN and elastin.

**Figure 9:**
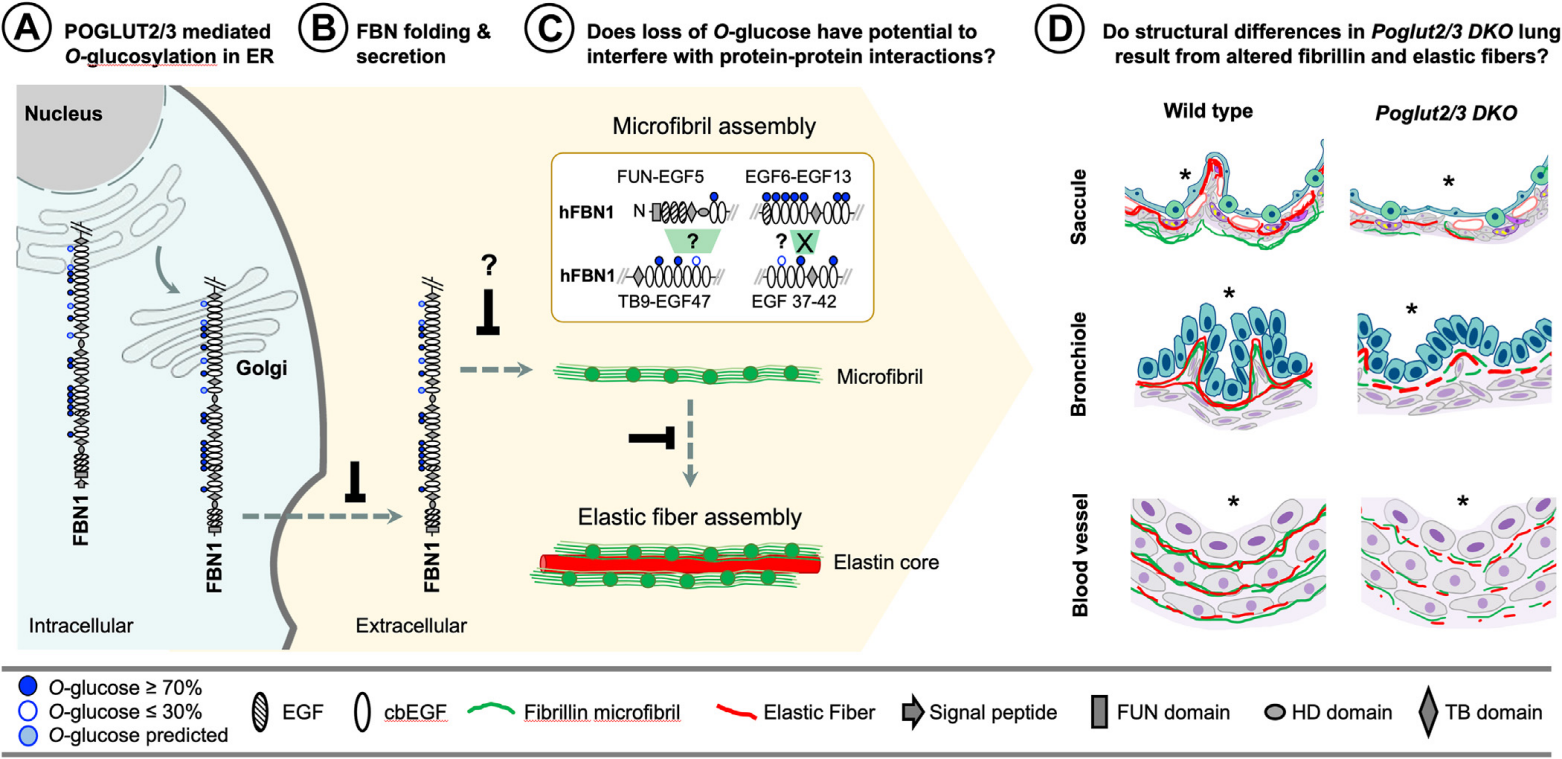
**Loss of POGLUT2/3-mediated *O-*glucosylation likely impacts secretion and extracellular function of FBNs in lung development.***A*, POGLUT2/3 mediated *O*-glucosylation (*blue circles*) of fibrillin EGF domains (hatched, non-calcium binding and white ovals, calcium-binding) occurs in the endoplasmic reticulum (ER). *B* and *C*, reduced secretion of FBNs into the extracellular space likely contributes in part to a deficit in microfibrils and elastic fibers. *C top*, Defective fibrillin intermolecular interactions (*green polygon*) could potentially impair microfibril assembly, transglutaminase (TG) cross-linking (*green polygon* with X), and/or physical properties of FBN microfibrils. *C bottom*, Altered fibrillin microfibril assembly would lead to deficit in elastic fibers. “?” marks indicate where future studies are required. Interactions shown are based on previous reports using human FBN1 (hFBN1) fragments (16, 18, 71). *D*, defects in microfibril and elastic fibers likely contributed to structural defects saccule (or bronchiole branching), flattened epithelial folds in bronchiole, and structurally compromised blood vessel in the *Poglut2/3 DKO* lung. ∗ indicates lumen of saccule, bronchiole and blood vessel. ER, endoplasmic reticulum, FUN, fibrillin unique N-terminal.

Similar *O-*linked glycosylation of folded Notch EGFs mediated by POFUT1 (*O-*fucose), POGLUT1 (*O-*glucose), and EOGT (*O-*GlcNAc) stabilizes EGFs to facilitate their trafficking (21, 61, 62). Like these enzymes, POGLUT2 and POGLUT3 are localized to the ER and only modify folded EGFs (15), suggesting a role for POGLUT2/3-mediated *O-*linked glucosylation in promoting efficient folding and trafficking of FBNs (Fig. 9, *A* and *B*). One potential mechanism by which *O-*linked glucosylation could stabilize the EGF fold is by optimizing calcium binding (63, 64). Several amino acids in the POGLUT2/3 consensus sequence are known to coordinate calcium binding (14, 65, 66, 67), and calcium binding influences the stability and rigidity of FBN1 microfibrils (14, 63, 66, 68, 69).

The striking reduction of FBN levels in primary dermal fibroblast ECM and *Poglut2/3 DKO* E18.5 lung tissue compared to the modest decrease in secretion from primary fibroblasts (Figs. 4, 5 and 8) raises the possibility that *O*-glucose modifications on FBN EGFs could facilitate the assembly of FBN microfibrils (Fig. 9*C*) [5, 58]. Given that a similar type of EGF *O-*linked glycosylation (*O-*fucose) was identified in the binding interface between Notch and its ligands’ protein modules (68, 70), these *O-*linked glucose modifications on FBN EGFs have the potential to modulate interactions needed for FBN structure and/or microfibril assembly in the extracellular space (Fig. 9*C*). Several FBN1 EGFs located in regions involved in the assembly of FBN microfibrils are *O*-glucosylated by POGLUT2/3 (18, 19, 71). Alternatively, loss of *O-*glucose could alter interactions between FBN and associated proteins such as LTBPs which could negatively impact microfibril assembly or function (Fig. 9*C*) (47). Consistent with this prediction, we observed reduced levels of other microfibril-associated proteins in the dermal fibroblast ECM (Fig. 8*C*). MFAP2 (MAGP1), which normally colocalizes and interacts with FBN1 (72, 73), could be reduced in the ECM as a consequence of lower levels of FBN1 in the *Poglut2/3 DKO.* While the increased level of EMILIN1, which is normally deposited on FBN1 and required for elastic fiber formation (42), could potentially result from upregulation in response to the decrease in FBN1 and elastic fibers in the *Poglut2/3 DKO*.

Since FBN microfibrils serve as a template to form elastic fibers (reviewed in (74)), the reduction and fragmentation of elastic fibers in E18.5 *Poglut2/3 DKO* lungs likely resulted from a primary defect in FBN microfibrils (Fig. 9*C*). Defects in elastic fibers could contribute to loosely arranged layers in the blood vessel and flatter epithelial folds in the bronchiole of *Poglut2/3 DKO* lung (Fig. 9*D*) by altering physical properties or mechanical forces required for lung maturation (38). However, it is also possible that defects in other secreted, unglucosylated POGLUT2/3 substrates such as FBLN4, FBLN5, and LTBP4 also contributed to defects in the elastin network of *Poglut2/3 DKO* lung (3, 27, 60, 75, 76, 77). During elastic fiber formation, FBLN4 and -5 are required for the coacervation of tropoelastin and to facilitate the deposition of tropoelastin onto microfibrils (5, 6). FBLN4 and -5 each have six total EGF repeats with two and three EGFs predicted to be modified by POGLUT2/3, respectively. LTBP4 contains 16 total EGFs with 12 predicted to be modified by POGLUT2/3. This elastin network is critical for the development of the lung’s complex alveolar architecture (3, 78, 79, 80) and for blood vessel development and function (81, 82). Mutations in *Fbln5, Fbln4,* and *Ltbp4* cause either fragmented elastic fibers or elastin aggregates in the lung with enlarged airspaces (24, 27, 59, 60), similar to what we observed in *Poglut2/3 DKOs*.

Collectively, the results from this study suggest that POGLUT2/3-mediated *O-*glucosylation on endogenous FBNs is not only required for efficient secretion but is also necessary for FBN extracellular function. Future studies are needed to determine whether the *O-*linked glucose on FBN EGFs is important for the assembly and/or stability of FBN microfibrils. In addition, it will be important to determine whether the *O-*linked glucose facilitates intermolecular interactions between FBN and other POGLUT2/3 substrates. This includes but is not limited to the LTBPs and fibulins, which are important for organizing FBN microfibrils and elastic fibers and modulating TGFβ signaling (5, 9, 42). CryoEM structural studies that can resolve the glycan-peptide interactions combined with atomic force microscopy, as recently described (83, 84), will help to better understand the extracellular role of the *O-*glucose for FBN function.

## Experimental procedures

### Ethics statement

All animal research was carried out in accordance with relevant national and international regulations and protocols. The Office of Laboratory Animal Welfare (OLAW) assurance number (D-16-00006) at Stony Brook University was approved by the National Institutes of Health (A3011-01). The OLAW assurance number (D16-00276) at the University of Georgia was approved by NIH (A3437-01). The animal studies were approved by the University of Georgia and Stony Brook University Institutional Animal Care and Use Committees (IACUC), which followed all of the guidelines outlined in: the Public Health Service Policy on Humane Care and Use of Laboratory Animals, distributed by the National Institutes of Health's Office of Laboratory Animal Welfare; Animal Welfare Act and Animal Welfare Regulations, distributed by the United States Department of Agriculture; and Public Health Service Policy on Humane Care and Use of Laboratory Animals, distributed by the Office of Laboratory Animal Welfare, NIH; Animal Welfare Act and Animal Welfare Regulations distributed by United States Department of Agriculture and Guide for the Care and Use of Laboratory Animals distributed by the National Research Council. The University of Georgia and Stony Brook University animal facilities are accredited by the Association for the Assessment and Accreditation of Laboratory Animal Care (AAALAC International).

### Mice and genotyping

For *Poglut2* (formerly *Kdelc1*; MGI:1919300), heterozygous *Poglut2*^*tm2a(EUCOMM)Hmgu*^ (*knockout-first*) ES cells were purchased from EuMMCR. *Poglut3* (formerly *Kdelc2*; MGI:1923765) targeting vector (PG00252_Z_1_B04) (*Poglut3*^*tm380258*(L1L2_Bact_P)^) was purchased from EuMMCR and used to generate *Poglut3*^*tm1Rsh*^ (*knockout-first*) heterozygous ES cells generated at Texas A&M Institute for Genomic Medicine using ES cell line JM8A3 (C57BL/6N). Targeted ES cells were injected into C57BL/6N blastocysts at the Mouse Transgenic and Gene Targeting Core at Emory University, and resultant chimeras were mated to females of the same strain of mice to generate animals heterozygous for the *Poglut2*^*tm2a(EUCOMM)Hmgu*^ (MGI:5296598) and *Poglut3*^*tm1Rsh*^ (MGI:7465084) *Knockout First* alleles (floxed null, neo in, reporter) (Fig. S1). *Poglut2*^*tmd2b(EUCOMM)Hmgu*^ (MGI:7465077) and *Poglut3*^*tm1.1Rsh*^ (MGI:7465085) null reporter alleles were generated using Cre-recombinase (B6.C-*Tg(CMV-cre)1Cgn*/J; common name: CMV-cre) (https://www.jax.org/strain/006054). The *Poglut2*^*tm2c(EUCOMM)Hmgu*^ (MGI:7465078) and *Poglut3*^*tm1.2Rsh*^ (MGI:7465086) floxed; reporter and neo removed, alleles were generated from the appropriate *Knockout First* allele using the mouse codon-optimized FLP (FLPo) flippase (B6.129S4-*Gt(ROSA)26Sor*^tm2(FLP^∗^)Sor^/J; common name: ROSA26Flpo) (https://www.jax.org/strain/012930). The *Poglut2*^*tm2d(EUCOMM)Hmgu*^ (MGI:7465080) and *Poglut3*^*tm1.3Rsh*^ (MGI:7465087) null (KO) alleles were subsequently generated from the appropriate floxed; reporter and neo removed, allele using the same CMV-cre line as above. The Maps of alleles and primers used for genotyping are in Fig. S1. Detailed primer information and PCR conditions are found in Table S1. Only the *Poglut2*^*tm2d(EUCOMM)Hmgu*^ and *Poglut3*^*tm1.3Rsh*^ single and double knockout alleles were analyzed for this paper. All alleles for *Poglut2* and *Poglut3* were maintained at the University of Georgia and Stony Brook University by backcrossing to C57BL/6J.

### Whole-mount imaging

The whole-mount image of P28 and E18.5 embryo, limbs and eyes were photographed immersed in PBS on a low melting agarose bed using a Zeiss Discovery V8 microscope, AxioCam MRc camera and AxioVisionLE program (Zeiss). The presence of syndactyly in limbs and the presence of eye/pupil abnormalities were determined by inspecting the photographs. Data were presented as photographs and graphs.

### Primary fibroblast cultures

Lung and tail tissues were collected at E18.5 or post-weaning. Tissues were minced with sterile surgical blades into approximately 1 mm pieces. Tissue pieces were transferred to 1 ml vials containing 2.5 mg/ml Collagenase D (Roche) in DMEM high glucose media supplemented with 10% fetal calf serum and 1% penicillin/streptomycin (complete medium). Samples were incubated 45 to 60 min, shaking 200 rpm, at 37^o^C. Seventy-micron cell strainers attached to 50 ml conical tubes were pre-wet with 10 ml of complete media. Tissues were passed through cell strainers using a double-sided pestle. Cells were pelleted at 600*g* for 6 min at room temperature. The supernatant was removed and cells were washed once with 10 ml of complete media. Cells were pelleted again at 600 x g for 6 min at room temperature. Cells were resuspended in 1 ml of complete medium. Cell suspension was added to 1 ml of complete medium in a single well of a 6-well plate or to 2 ml of complete medium in a 6 cm dish. After 24 to 48 h, the medium was removed, cells were rinsed with PBS, and a fresh complete medium was added. The medium was changed every 2 to 3 days until confluency. All cells were maintained at 37 °C and 5% CO_2_ in a complete medium. For glycoproteomic and proteomic experiments, fibroblast cultures no older than three passages were grown to confluency in 6 cm dishes. At confluency, the medium was switched to DMEM high glucose media with no serum or antibiotics. After 3 days, the medium was collected, cleared, and stored at −20 °C for mass spectral analysis. For analysis of the extracellular matrix, cells were grown to confluency in 10 cm dishes, then maintained in DMEM high glucose media for approximately 1 week. The medium was removed, cells were rinsed with PBS, and incubated with 0.5% Triton X-100, 20 mM NH_4_OH in PBS for 5 to 10 min to decellularize plates. Plates were washed 3 times with 8 ml of PBS. Approximately 0.5 ml of 8 M Urea, 400 mM ammonium bicarbonate, and 10 mM tris(2-carboxyethyl)phosphine (TCEP) (warmed for 5 min at 50 °C) was added to dishes. Dishes were scraped and collected for mass spectral analysis.

### Immunoprecipitation of FBN1 from adult mouse primary dermal fibroblast medium

Immunoprecipitations were performed as previously described (14). Briefly, 50 μl of magnetic beads (Protein G Dynabeads, Invitrogen) were incubated with 5 μl anti-FBN1 antibody (generously supplied by Dr Dieter Reinhardt, diluted in 200 μl 1 × PBS, 0.02% Tween) for 20 min rotating at room temperature. Beads were then washed in 1X PBS, 0.02% Tween 20, and resuspended in 1 ml of cleared, conditioned medium and incubated for 1 h at room temperature, rotating. After three washes, beads were eluted in 30 μl of 8 M urea in 400 mM ammonium bicarbonate at 37 °C for 15 min. Elution fractions were stored at −20 °C.

### Mass spectral analysis

BCA assays were used to determine the protein concentration of each sample. Media volume equivalent to 10 μg was acetone precipitated in ice-cold acetone (1:4 media:acetone) overnight at −20 °C. Samples were spun down at max speed, 10 min, 4 °C. The supernatant was removed, and the pellet was processed for mass spectral analysis. Equivalent protein amounts of extracellular matrix samples were used. Media samples were denatured and reduced using 15 μl of reducing buffer containing 8 M Urea, 400 mM ammonium bicarbonate, and 10 mM TCEP at 50 °C for 5 min. Alkylation of both media and extracellular matrix samples was performed at room temp by adding 100 mM iodoacetamide in 50 mM TrisHCl, pH 8.0, to a final concentration of approximately 33 mM iodoacetamide and incubating in the dark for 30 min to 1 h. Mass spectral grade water was added to each sample to dilute urea to approximately 1 M. 500 ng of trypsin (cleaves C-terminal to lysine and arginine, Thermo Scientific Pierce 90057) protease was added per sample. Samples were incubated in 37 °C water bath for 3 to 4 h. Samples were acidified with 5% of formic acid and sonicated for 15 min. Samples were desalted with Millipore C18 Zip Tip Pipette Tips. After elution in 50% acetonitrile, 0.1% acetic acid, samples were diluted to an approximate concentration of 1 μg/μl, 15% acetonitrile, and 0.1% formic acid. Approximately 2 to 3 μg of each sample was injected on a Q-Exactive Plus Orbitrap mass spectrometer (Thermo Fisher) with an Easy nano-LC HPLC system with a C18 EasySpray PepMap RSLC C18 column (50 μm × 15 cm, Thermo Fisher Scientific). A 90-min binary gradient solvent system (Solvent A: 0.1% formic acid in water and Solvent B: 90% acetonitrile, 0.1% formic acid in water) with a constant flow of 300 nl/min was used. Positive polarity mode was used with an m/z range of 350-2000 at a resolution of 35,000 and automatic gain control set to 1 × 10^6^. Higher energy collisional dissociation-tandem mass spectrometry (HCD-MS/MS) was used on the top 10 precursor ions in each full scan (collision energy set to 27%, 2 × 10^5^ gain control, isolation window m/z 3.0, dynamic exclusion enabled, and 17,500 fragment resolution.

For glycoproteomic analysis, PMi-Byonic (v4.1.10) was used to identify glycopeptides. Fixed modifications: Carbamidomethyl +57.021464 at C. Variable modifications: Oxidation +15.994915 at M,N,D, Deamidated +0.984016 at N,Q, and Ammonia-loss −17.026549 at N-Term C. Precursor mass tolerance was set to 20 ppm and fragment mass tolerance was set to 10 ppm. Two missed cleavages were allowed. Protein and peptide false discovery rates were set to a threshold of 1% and calculated using the 2-dimensional target decoy strategy as described (53). Initial searches were performed against the mouse proteome database (see Proteomic analysis below) to identify POGLUT2/3 substrates in the media. Subsequently, all data was searched against a mouse FBN1 database (UniProt accession number Q61554 version 174 updated July 6, 2016, one entry), FBLN2 (UniProt accession number P37889 version 185 updated October 3, 2012, one entry), NID1 (UniProt accession number P10493 version 217 updated July 27, 2011, one entry), HMCN1 (UniProt accession number D3YXG0 version 97 updated April 20, 2010, one entry), FBLN5 (UniProt accession number QPWVH9 version 181 updated November 1, 1999, one entry), and SVEP1 (UniProt accession number A2AVA0 version 108 updated February 5, 2008, one entry). Peptide libraries generated in Byonic and RAW mass spectral files for each sample run were imported into Skyline (v20.2), which was used to generate Extracted Ion Chromatograms (EICs) for all identified peptides. For each peptide, area under the curve was calculated for each peak corresponding to the searched glycoforms (Dataset S1 and Excel file 1). Relative abundance was calculated by comparing the area under the curve for a single glycoform to the total area under the curve for all searched glycoforms of a specific peptide. Glycoforms searched: unmodified peptide, unmodified peptide plus β-hydroxylation, modified peptide with *O*-hexose, and modified peptide with *O*-hexose plus β-hydroxylation.

For proteomics analysis, Proteome Discoverer (v2.5) was used to identify and quantify peptides (52). The default Comprehensive Enhanced Annotation LFQ and Precursor Quantitation workflow was used. Precursor abundance was based on peak intensity. Protein abundance calculations were performed using the Summed Abundance approach where protein abundances are calculated using the abundances of associated peptide groups. The minimum peptide length was set to 6, and the false discovery rate was set to 1%. Precursor mass tolerance was 10 ppm and fragment mass tolerance was 0.02 Da. PSM Confidence was set to “at least High” to filter out low-scoring peptide matches. A minimum of three unique peptides had to be identified for a protein to be considered. Two missed cleavages were allowed. Static modifications: Carbamidomethyl +57.021464 at C. All data was searched against the reviewed *Mus musculus* proteome from UniProt (Reviewed 17,114 proteins Swiss Prot). Data was moved into Excel for statistical analysis. Gene Ontology (GO) was used to select for “extracellular region” proteins only, filtering out detected intracellular proteins (85, 86, 87). All samples were normalized to the sample with the highest protein abundance. Average abundance was calculated for each protein, and fold change was determined by dividing the *Poglut2/3 DKO* average by the wild-type average for each protein. A two-tailed *t* test was used to calculate statistical significance. Only proteins that were identified in at least half of control samples and half of *Poglut2/3 DKO* samples were plotted in volcano plots.

### Histology and immunohistochemistry

Histology and immunostaining were performed on 5 μm thin paraffin sections prepared from E18.5 left lungs that were either fixed in 4% paraformaldehyde or 5% acetic acid in ethanol (volume/volume) as previously described (88). Briefly, paraformaldehyde-fixed lungs were dehydrated through graded series of ethanol:30%, 50%, 70%, 80%, 90%, 95%, and 100%, and then xylene, whereas acetic acid fixed tissues were directly transferred to 100% ethanol and then xylene. Tissues were then processed through xylene and paraffin (1:1) mix and infiltrated with and embedded in paraffin and processed for sectioning using a microtome.

Sections were routinely stained with hematoxylin and eosin (H&E) staining as previously described (89). Briefly, sections were deparaffinized in xylenes and rehydrated using ethanol (100%, 95%, 80% and 70%), washed with reverse osmosis water for 10 min and stained in Mayer’s hematoxylin (Sigma, Cat. no. MHS32-1 L) for 10 min and washed in running tap water for 20 min. The sections were again passed through 70% ethanol for 2 min and counterstained with eosin (Fisher Scientific Cat. no. E511-25) for a minute. Finally, sections were dehydrated for 2 min each in 70%, 95%, 100%, 100%, and 100% ethanol, and cleared in xylenes before mounting. Sections were mounted with Secure Mount (Fisher Scientific, Cat. no. 23-022208) and coverslipped.

Elastic fiber was stained using the Orcinol-New Fuchsin technique as described previously (90). Briefly, deparaffinized sections were hydrated using running tap water and incubated with freshly prepared Orcinol-New Fuchsin [Orcinol (Sigma, Cat. no. 447420), New Fuchsin (Sigma, Cat. no. 72200), Ferric chloride (Sigma, Cat. no. 2364890)] solution at 37 °C for 15 min. The sections were washed three times with 70% ethanol for 5 min each and counter-stained with Weigert’s Iron Hematoxylin [1:1mixture of Weigert’s Iron Hematoxylin A and B (Electron Microscopy Sciences, Cat. no. 236370)] for 5 min and washed with running tap water. The sections were then stained with Van Gieson’s solution (Electron Microscopy Sciences, Cat. no. 26370) for 1 min, dehydrated in ethanol, cleared in xylene, and mounted and coverslipped.

Slides adjacent to H&E stained slides (Fig. 3, *I* and *M*) showing the pulmonary vein, bronchiole, pulmonary artery, and saccule were used for all immunohistochemistry staining. Sections were deparaffinized using xylenes and rehydrated using ethanol (100%, 95%, 80%, and 70%), rinsed with water, and finally with 1X PBS. The immunofluorescence on lung paraffin sections was performed as previously described (88, 89). The sections were incubated with primary antibodies followed by secondary antibodies (Table S4). Slides were mounted with DAPI Fluoromount-G (SouthernBiotech, Cat. no. 1000-20) and coverslipped.

The H&E-stained histological sections were photographed using a Nikon Optiphot microscope, AxioCam MRc camera, and AxioVisionLE program (Zeiss). Fluorescent images were taken at 63 × using Leica TCS SP8 X scanning confocal microscope (Leica, Germany) and processed using Leica Application Suite X or Imaris. ImageJ (http://imagej.net/) was used to measure the level of fluorescence. Immunofluorescence signals from individual images was normalized with DAPI signals from the same image. The same immunostained image from wild-type animals was presented to compare with *Poglut2/3 DKO*, *Poglut2 KO,* and *Poglut3 KO.*

For colocalization analysis the correlation index (Pearson’s correlation coefficient) of colocalized pixels between the “green (fibrillins) and red (elastin)” colors were measured in ImageJ and plotted as a graph in GraphPad Prism as described previously (89). Briefly, the color of the image was split into component channels, and “green and red” channels were used to evaluate the colocalization using ImageJ plugin “Colocalization threshold” which produced the correlation coefficient of colocalization. The higher the correlation coefficient, the higher will be the colocalization, and *vice versa*.

### RNAscope assay

Embryonic day 18.5 mouse left lung was dissected and fixed overnight in 4% PFA and processed for paraffin sectioning (5 mm). RNAscope assay was carried out as previously described (89) and according to Advanced Cell Diagnostics’ instructions using *Poglut2* (1052881-C1) and *Poglut3* (1052891-C1) probes. ACD HybEZ II Oven (Advanced Cell Diagnostics, Inc, Hayward, CA) was used for the probe hybridization. Probe binding was visualized by using RNAscope 2.5 HD Detection Kit (Red) using Mayer’s hematoxylin as a counterstain. The bacterial gene encoding dihydrodipicolinate reductase (DapB; 310043) and *M. musculus* gene encoding peptidylprolyl isomerase B (Ppib; 313911) were used as negative and positive controls (Fig. S3), respectively.

### RNA extraction and real-time quantitative RT-PCR (qRT-PCR)

RNA extraction and qRT-PCR were performed as described previously (88, 89). Briefly, total RNA was extracted from E18.5 wild-type lung using RNeasy Mini Kit (Qiagen, ID 74104), total of 2 μg RNA from each sample was reverse transcribed using SuperScript VILO cDNA Synthesis Kit (Invitrogen, Cat. no. 11754050) and qRT-PCR was carried out using PowerTrack SYBR Green Master Mix (Thermo Fisher Scientific, Cat. no. A46109) on StepOnePlus Real-Time PCR System. The results from control (n = 3; three replicates per sample) and mutant (n = 3; three replicates per sample) animals for each gene were normalized to those with *Gapdh* and expressed as fold change ± standard deviations. The mean expression levels from control and mutants were compared using Student’s *t* test. The primers of genes used were listed in Key Resources Table and Table S5.

### Transmission electron microscopy

Lungs were initially fixed in 10% neutral buffered formalin (Electron Microscopy Sciences, EMS), then transferred to 2.5% glutaraldehyde (EMS) in 1x PBS for 1 week at 4 °C and processed as previously described (91). Briefly, samples were washed in PBS, post-fixed with 1.25% osmium tetroxide (EMS), and then stained sequentially with 2% tannic acid (Sigma-Aldrich) and 6% uranyl acetate (EMS). Samples were washed again and dehydrated in a graded series of ethanol. Samples were infiltrated in a graded series of resin and propylene oxide (PolySciences), then embedded in fresh resin. Blocks were sectioned at 60 nm thickness, collected on formvar coated grids, post-stained with 6% uranyl acetate and Reynold’s lead citrate, and imaged on a JEOL 1400 electron microscope with an AMT XR111 digital camera. The *Poglut2 KO; Poglut3 heterozygous* mice used to obtain *Poglut2/3* double knockout embryos were generated from intercrosses using C57BL/6J backcross generations N0 through N2. Strain/Stage matched wild type animal was used as a control.

### Statistical analysis

Power analysis was performed using FBN1 fluorescence data from the wild type and *Poglut2/3 DKO* saccule region to determine the required sample size. The power analysis showed that total sample size of 6, three each from wild type and *Poglut2/3 DKO* would give > 98% power. The analysis was calculated using G∗Power 3.1.9.7 (92). All histology, immunohistochemistry, and RNA analyses in this study were performed using a left lung isolated from at least three embryos per genotype (n = 3–6) with 2 to 6 sections per embryo. Statistical significance was determined using GraphPad Prism version 8.0.0 for Windows, GraphPad Software. Data were evaluated for significance using an unpaired Student’s *t* test. Data are expressed as mean ± standard deviation (SD) with *p*-value <0.05 considered significant (Excel file 4). For mass spectral analyses, the numbers of biological and technical replicates are indicated in the respective figure legends.

## Data availability

The mass spectral proteomic data was deposited to the ProteomeXchange Consortium *via* the PRIDE (93) partner repository (https://www.ebi.ac.uk/pride/archive) with the data set identifier PXD043730.

The glycoproteomic data have been deposited to Panorama Public through the PanoramaWeb archive with the permanent link https://panoramaweb.org/JxIx9s.url. The data were copied to the Proteome Exchange Consortium with the data set identifier PXD043827.

## Supporting information

This article contains supporting information.

## Conflict of interest

The authors declare that they have no known competing financial interests or personal relationships that could have appeared to influence the work reported in this paper.
