# Supplementary material for: Poglut2/3 double knockout in mice results in neonatal lethality with reduced levels of fibrillin in lung tissues
Source: J Biol Chem. 2024 Jun 4;300(7):107445. doi: 10.1016/j.jbc.2024.107445 (PMC11261140; doi:10.1016/j.jbc.2024.107445)
Supplement: Supporting Tables [file mmc1.docx]

***Poglut2/3* double knockout in mice results in neonatal lethality with reduced levels of fibrillin in lung tissues**

Sanjiv Neupane^1†^, Daniel B. Williamson^2†^, Robyn A. Roth^3^, Carmen M. Halabi^3^, Robert S. Haltiwanger^2^*, and Bernadette C. Holdener^1^*

^1^Department of Biochemistry and Cell Biology, Stony Brook University, Stony Brook, NY, 11794-5215, USA

^2^ Complex Carbohydrate Research Center, Department of Biochemistry and Molecular Biology, University of Georgia, Athens, GA 30602, USA.

^3^Department of Pediatrics, Division of Nephrology, Washington University School of Medicine, St. Louis, MO 63110, USA

^†^These authors contributed equally.

*Co-corresponding authors: rhalti@uga.edu and bernadette.holdener@stonybrook.edu

**Contents:**

Supplemental Tables S1-S5

**Supplemental Tables**

**Table S1. Primers and PCR conditions for genotyping *Poglut2* mice**

| *Poglut2* allele | Primer Name & [concentration] | Primer Sequence 5’-3’ | PCR Product size (bp) | PCR Conditions |
| --- | --- | --- | --- | --- |
| *Wild type (WT)* | K1 5’ F (a)  [0.25 μm] | gcctttagtagctgagacatctctc | 496 | 95ºC—3 min  95ºC—30 sec  60ºC—30 sec  70ºC—30 sec  (35 cycles)  70ºC—5 min  4ºC—hold |
|  | K1 3’ R (b)  [0.25 μm] | cacagcggtgaacatctcacttaacacc |  |  |
| *Knockout-first (tm2a)* | K1 5’ F (a)  [0.25 μm] | gcctttagtagctgagacatctctc | 279 |  |
|  | LAR3 (c)  [0.25 μm] | caacgggttcttctgttagtcc |  |  |
| *lacZ-Δ3-4 (tm2b)* | CSD Lac F (d)  [0.25 μm] | gctaccattaccagttggtctggtgtca | 386 |  |
|  | K1 3^rd^ LoxP R (e)  [0.25 μm] | caccacaatgaactgatggcgag |  |  |
| *floxed3-4*  *(tm2c)* | K1 5’ F (a)  [0.25 μm] | gcctttagtagctgagacatctctc | 630 |  |
|  | K1 3’ R (b)  [0.25 μm] | cacagcggtgaacatctcacttaacacc |  |  |
| *KO*  *(tm2d)* | K1 5’ F (a)  [0.25 μm] | gcctttagtagctgagacatctctc | 259 |  |
|  | K1 3^rd^ LoxP R (e)  [0.25 μm] | caccacaatgaactgatggcgag |  |  |

**Table S2. Primers and PCR conditions for genotyping *Poglut3* mice**

| *Poglut3* allele | Primer Name & [concentration] | Primer Sequence 5’-3’ | PCR Product size (bp) | PCR Conditions |
| --- | --- | --- | --- | --- |
| *Wild type*  *(WT)* | K2 5’ F (f)  [0.25 μm] | ggttactagacacttcaatggc | 353 | 95ºC—3 min  95ºC—30 sec  59ºC—30 sec  72ºC—30 sec  (35 cycles)  72ºC—3 min  4ºC—hold |
|  | K2 3’ R (g)  [0.25 μm] | aacgttgccatttcctgatcatt |  |  |
| *Knockout-First*  *(tm1Rsh)* | K2 5’ F (f)  [0.25 μm] | ggttactagacacttcaatggc | 241 |  |
|  | KOMP R (h)  [0.25 μm] | ggtggtgtgggaaagggttc |  |  |
| *lacZ-Δ2-3 (tm1.1Rsh)* | CSD Lac F (i)  [0.25 μm] | gctaccattaccagttggtctggtgtca | 497 |  |
|  | K2 b_d R (j)  [0.25 μm] | actcgtgtgagatccttgcc |  |  |
| *floxed2-3*  *(tm1.2Rsh)* | K2 5’ F (f)  [0.25 μm] | ggttactagacacttcaatggc | 561 |  |
|  | K2 3’ R (g)  [0.25 μm] | aacgttgccatttcctgatcatt |  |  |
| *KO*  *(tm1.3Rsh)* | K1 5’ F (f)  [0.25 μm] | ggttactagacacttcaatggc | 524 |  |
|  | K2 new b_d R (k)  [0.25 μm] | tcaccagctgctcatgcaata |  |  |

**Table S3. Viability of *Poglut2;Poglut3* double heterozygote intercross progeny at weaning**

| Intercross^a^: *Poglut2 WT/KO;Poglut3 WT/KO* | | | | | |
| --- | --- | --- | --- | --- | --- |
| Genotype | Weaning  # | Expected  # | Frequency (%) | | Chi square^b^ (p) |
| *Poglut2;Poglut3* |  |  | Weaning | Expected |  |
| *Het;Het* | 18 | 28 | 16.07 | 25 |  |
| *WT;Het* | 10 | 14 | 8.92 | 12.5 |  |
| *Het;WT* | 19 | 14 | 16.96 | 12.5 |  |
| *Het;KO* | 17 | 14 | 15.18 | 12.5 |  |
| *KO;Het* | 16 | 14 | 14.29 | 12.5 |  |
| WT;*WT* | 8 | 7 | 7.14 | 6.25 |  |
| *WT;KO* | 15 | 7 | 13.39 | 6.25 |  |
| *KO;WT* | 7 | 7 | 6.25 | 6.25 |  |
| *KO;KO* | 2 | 7 | 1.79 | 6.25 | 20.29 (0.0093) |

^a^Animals were generated from intercrosses using C57BL/6J backcross generation N0 through N2.

^b^Chi squared with 8 degrees of freedom

Abbreviations: WT, Wild type; KO, Knockout

**Table S4: Immunohistochemistry conditions**

| **Tissue** | **Fixative** | **Section type** | **Antigen** | **Antibody type/Vendor/Cat.#/RRID** | **Antigen Retrieval Method** | **1^0^ antibody Type & dilution** | **2^0^ antibody/Dilution/Vendor/Cat#/RRID** |
| --- | --- | --- | --- | --- | --- | --- | --- |
| E18.5 lung | 4% PFA | Paraffin | Fibronectin | Rabbit polyclonal/Abcam/ab2413/RRID:AB_2262874 | Boiling under pressure in Tris-EDTA buffer (pH 9.0; Tris 1.21 g, EDTA 0.37 g, distilled water 1 L, adjust pH 9.0, add 0.5 ml of tween 20l) for 10 minutes | 1:100 | Goat Anti-Rabbit IgG H&L (Alexa Fluor® 647/1:500/Abcam/ab150079/RRID:AB_2722623 |
| E18.5 lung | 4% PFA or 5% Acetic Acid in Ethanol | Paraffin | GRP78 BiP | Rabbit polyclonal/Abcam/ab21685/RRID:AB_2119834 | Boiling under pressure in Tris-EDTA buffer (pH 9.0; Tris 1.21 g, EDTA 0.37 g, distilled water 1 L, adjust pH 9.0, add 0.5 ml of tween 20l) for 10 minutes | 1:1000 | Goat Anti-Rabbit IgG H&L (Alexa Fluor® 647/1:500/Abcam/ab150079/RRID:AB_2722623 |
| E18.5 lung | 5% Acetic Acid in Ethanol | Paraffin | Fibrillin1 (FBN1) | Rabbit polyclonal (pAb 9543) (Courtesy of Dr. Lynn Sakai) | Permeabilization with 0.1% Triton-X-100 (EMD Chemicals, TX1568-1) in PBS for 30 minutes | 1:200 | Goat Anti-Rabbit IgG H&L (Alexa Fluor® 488/1:500/Abcam/ab150077/RRID:AB_2630356 |
| E18.5 lung | 5% Acetic Acid in Ethanol | Paraffin | Fibrillin2 (FBN2) | mFib2-Gly (Gly-Rich domain) polyclonal antiserum to mouse Fibrillin 2 (Courtesy of Dr. Robert Mecham) | Permeabilization with 0.1% Triton-X-100 (EMD Chemicals, TX1568-1) in PBS for 30 minutes | 1:500 | Goat Anti-Rabbit IgG H&L (Alexa Fluor® 488/1:500/Abcam/ab150077/RRID:AB_2630356 |
| E18.5 Lung | 4% PFA | Paraffin | LTBP1 | Anti-LTBP1 antibody (Rabbit Polyclonal)/Abcam/ ab78294/RRID:AB_1952060 | Permeabilization with 0.1% Triton-X-100 (EMD Chemicals, TX1568-1) in PBS for 30 minutes | 1:200 | Goat Anti-Rabbit IgG H&L (Alexa Fluor® 647/1:500/Abcam/ab150079/RRID:AB_2722623 |
| E18.5 lung | 4% PFA or 5% Acetic Acid in Ethanol | Paraffin | Elastin | Alexa Fluor™ 633 Hydrazide/Thermo scientific/ A30634/ |  | 0.2 μM/L | - |

**Table S5: Primers used in qRT-PCR**

| **Gene Name** | **Abbreviation** | **Accession #** | **Forward Primer (5' to 3')** | **Reverse Primer (5' to 3')** |
| --- | --- | --- | --- | --- |
| Protein  *O*-glucosyltransferase 2 | *Poglut2* | NM_023645.3 | CGTTGGCTTTAGGATTTTCATGG | AGATCGGCTGAATGTTGGAG |
| Protein  *O*-glucosyltransferase 3 | *Poglut3* | NM_212445.2 | TGAATTTTGCTGTCACTGGC | AGCCACACCAGGAAATGATAG |
| **Control gene:** |  |  |  |  |
| Glyceraldehyde  3-phosphate dehydrogenase | *Gapdh* | NM_008084 | TGGAAAGCTGTGGCGTGA | TGCTTCACCACCTTCTTGAT |
