# Supplementary material for: Poglut2/3 double knockout in mice results in neonatal lethality with reduced levels of fibrillin in lung tissues
Source: J Biol Chem. 2024 Jun 4;300(7):107445. doi: 10.1016/j.jbc.2024.107445 (PMC11261140; doi:10.1016/j.jbc.2024.107445)

FBN1 EGF 12

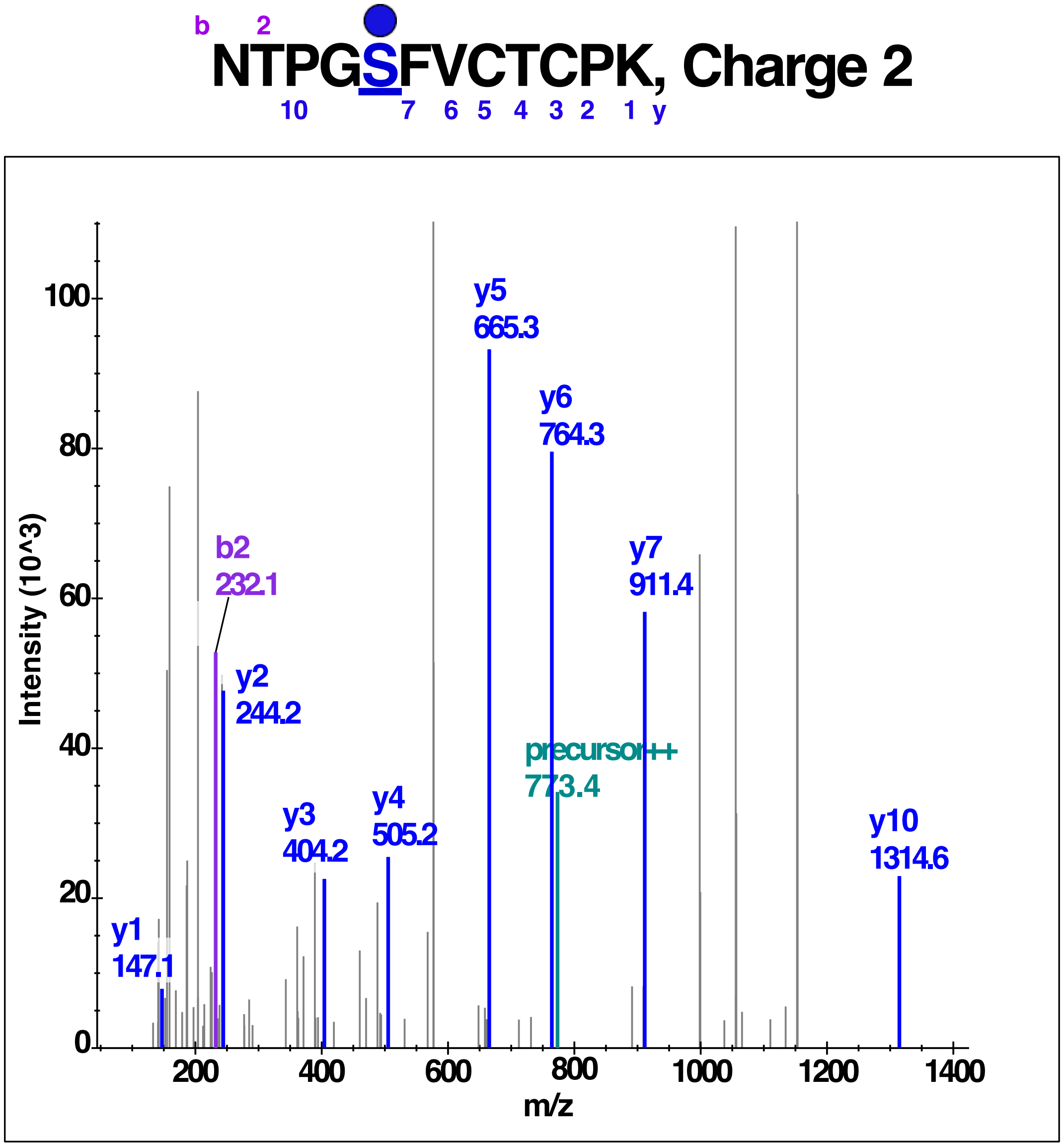

b 2
NTPGSFVCTCPK, Charge 2
10 7 6 5 4 3 2 1 y
y5
665.3
y6
764.3
y7
911.4
b2
232.1
y2
244.2
precursor++
773.4
y3
404.2
y4
505.2
y10
1314.6
y1
147.1
Intensity (10^3)
m/z

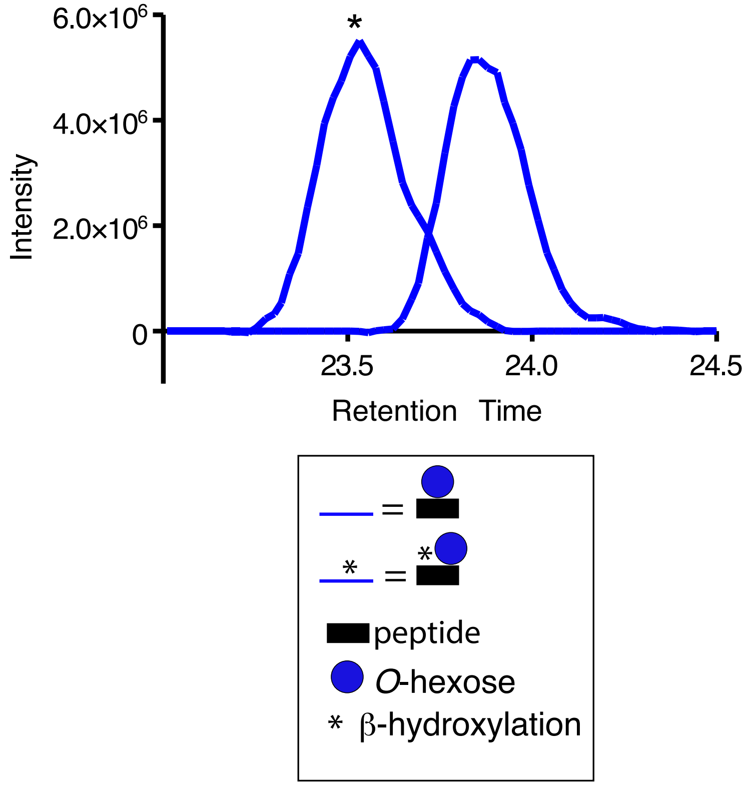

Intensity
Retention Time
6.0×10^6
4.0×10^6
2.0×10^6
0
23.5
24.0
24.5
peptide
O-hexose
* β-hydroxylation

FBN1 EGF 13

NSPGSFICECSPESTLDPTK, Charge 3

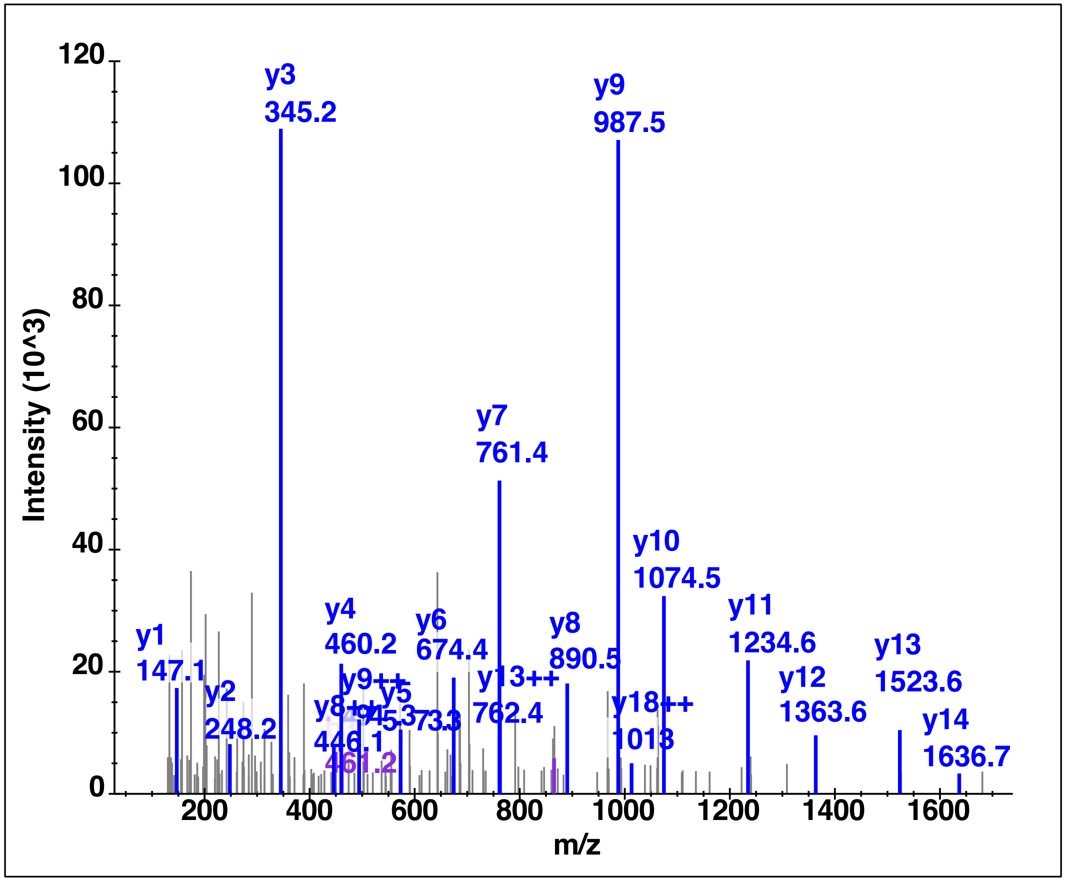

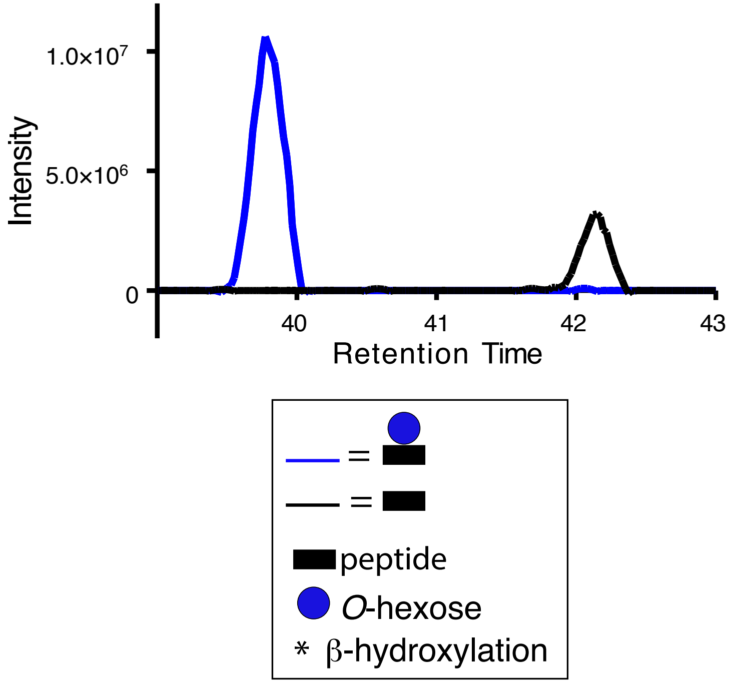

FBN1 EGF 21

b 2 3 4 6 8 9 10 11

GSFICHCDMGYSGK, Charge 3

7 2 1 y

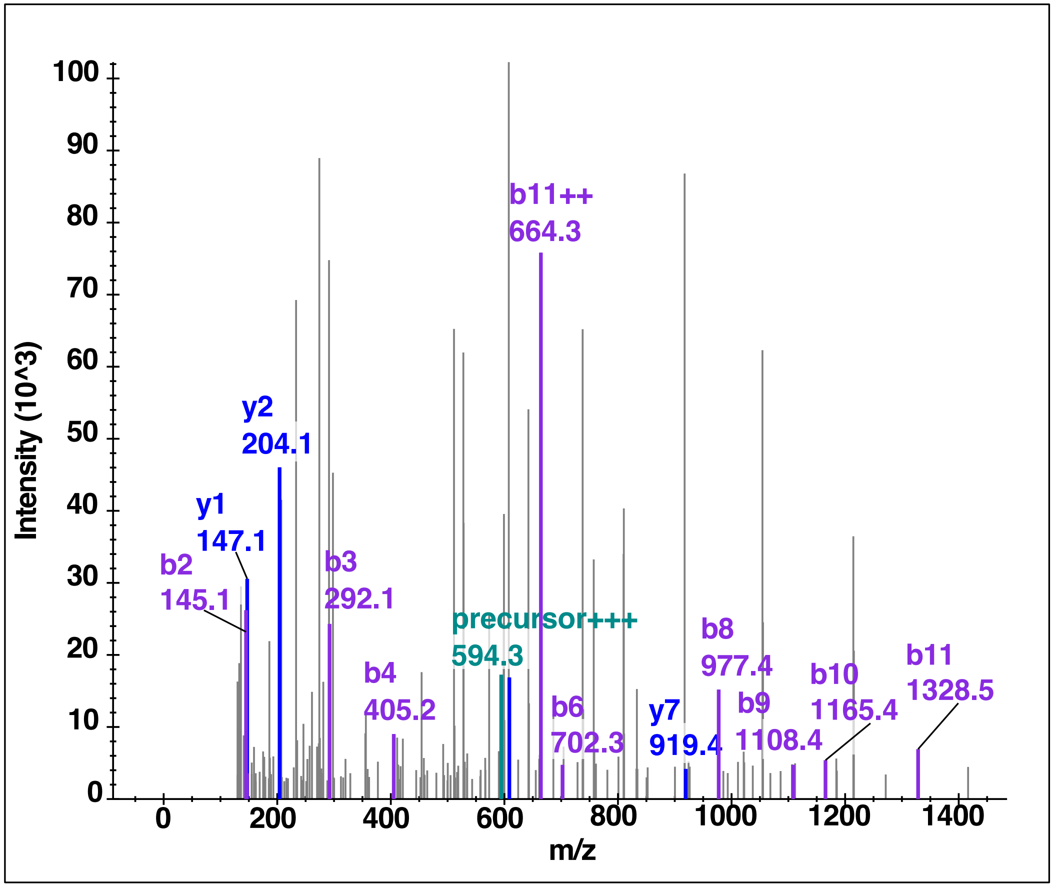

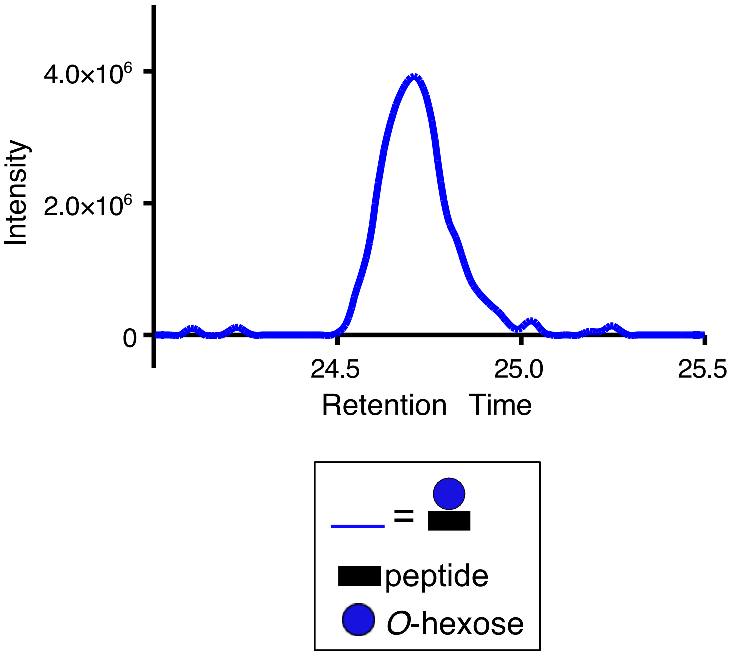

FBN1 EGF 22

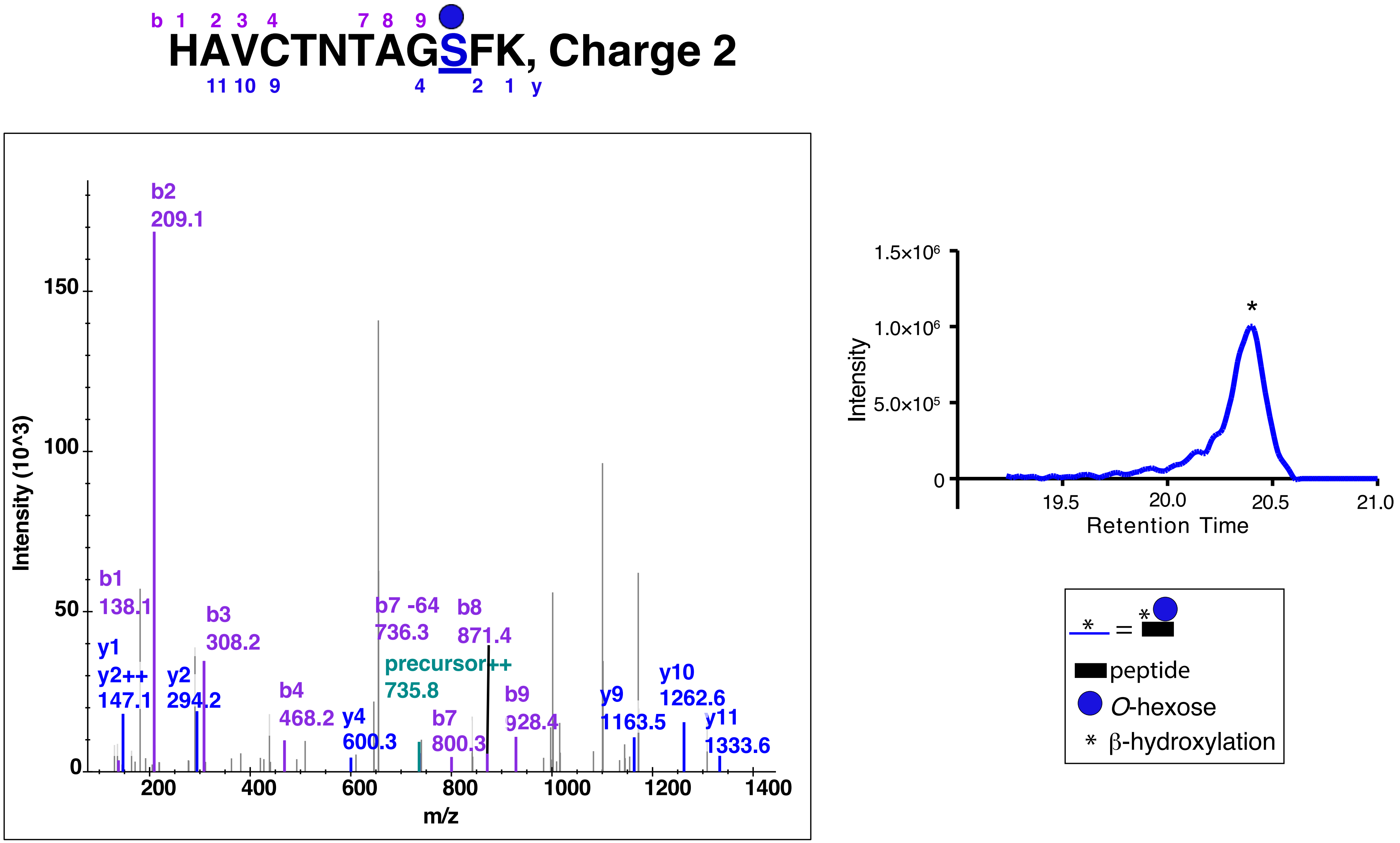

b 1 2 3 4 7 8 9
HAVCTNTAGSFK, Charge 2
11 10 9 4 2 1 y
b2 209.1
b1 138.1
b3 308.2
y1
y2++ 147.1
y2 294.2
b4 468.2
y4 600.3
b7 -64 736.3
precursor++ 735.8
b8 871.4
b7 800.3
b9 928.4
y9 1163.5
y10 1262.6
y11 1333.6
Intensity (10^3)
150
100
50
0
200
400
600
800
1000
1200
1400
m/z
Intensity
1.5×10⁶
1.0×10⁶
5.0×10⁵
0
19.5
20.0
20.5
21.0
Retention Time
*
peptide
O-hexose
* β-hydroxylation

FBN1 EGF 27

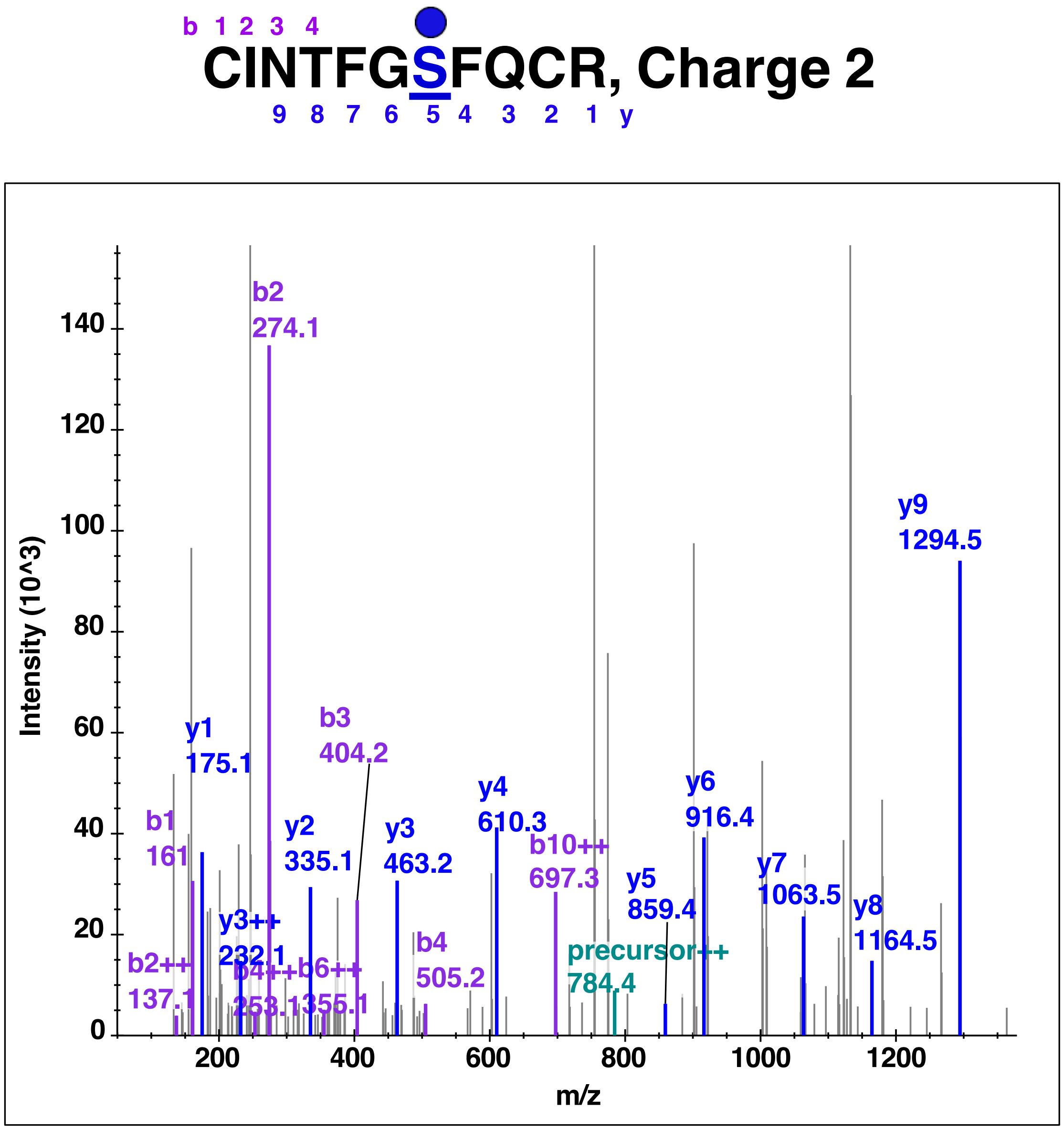

b 1 2 3 4
CINTFGSFQCR, Charge 2
9 8 7 6 5 4 3 2 1 y
Intensity (10^3)
m/z
b2 274.1
y9 1294.5
y1 175.1
b3 404.2
y4 610.3
y6 916.4
b1 161
y2 335.1
y3 463.2
b10++ 697.3
y5 859.4
y7 1063.5
y8 1164.5
y3++ 232.1
b4 505.2
precursor++ 784.4
b2++ 137.1
b4++ 253.1
b6++ 355.1

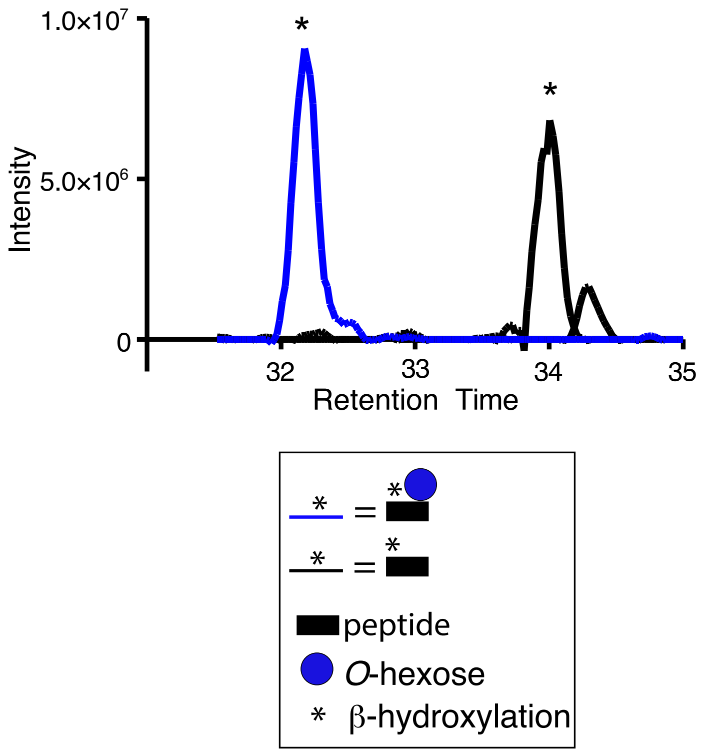

Intensity
1.0×10^7
5.0×10^6
0
Retention Time
peptide
O-hexose
* β-hydroxylation

FBN1 EGF 30

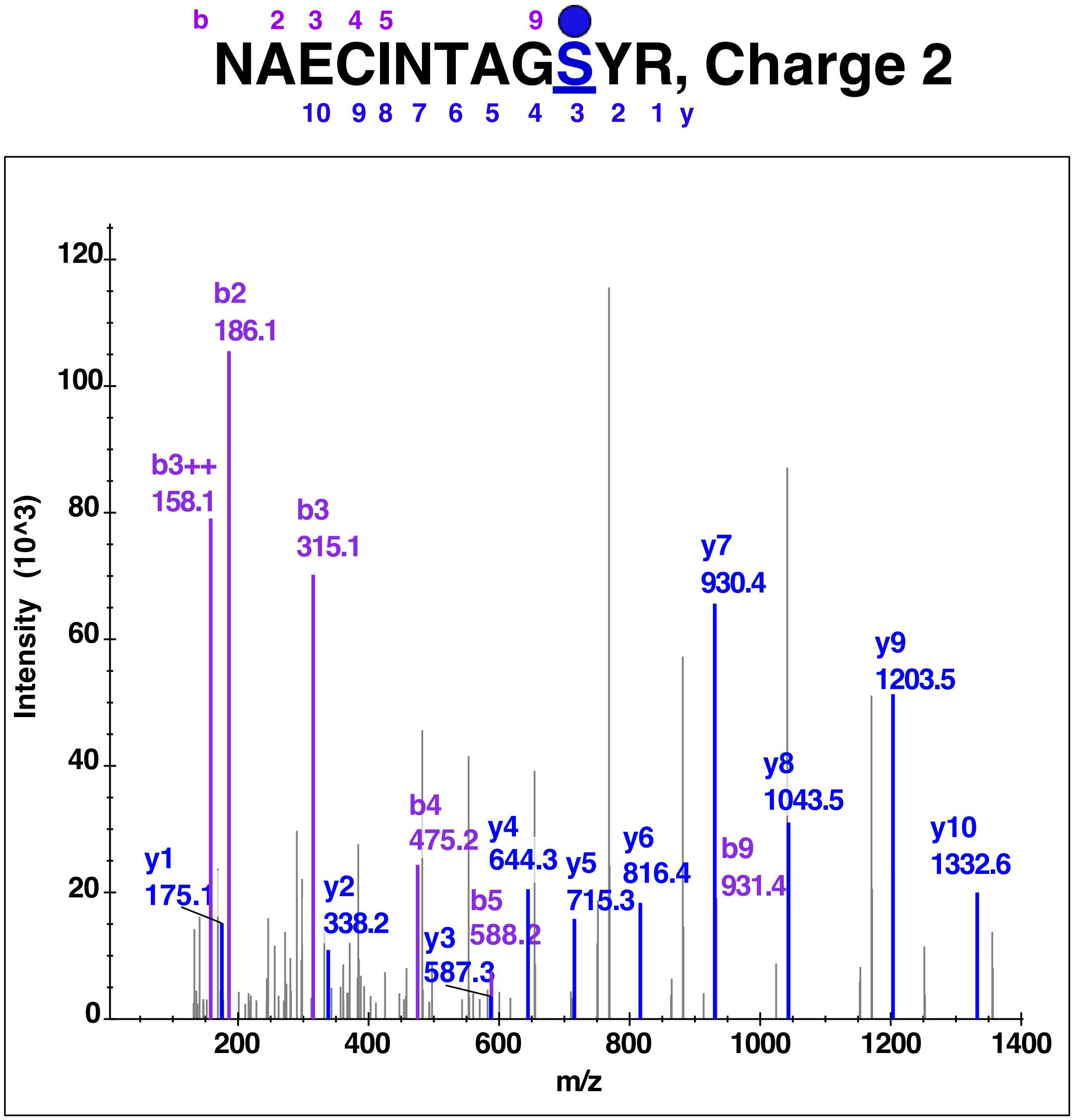
b 2 3 4 5 9
NAECINTAGSYR, Charge 2
10 9 8 7 6 5 4 3 2 1 y
Intensity (10^3)
m/z
b2 186.1
b3++ 158.1
b3 315.1
y7 930.4
y9 1203.5
y8 1043.5
y1 175.1
y2 338.2
b4 475.2
y4 644.3
y5 715.3
y6 816.4
b9 931.4
y10 1332.6
b5 588.2
y3 587.3

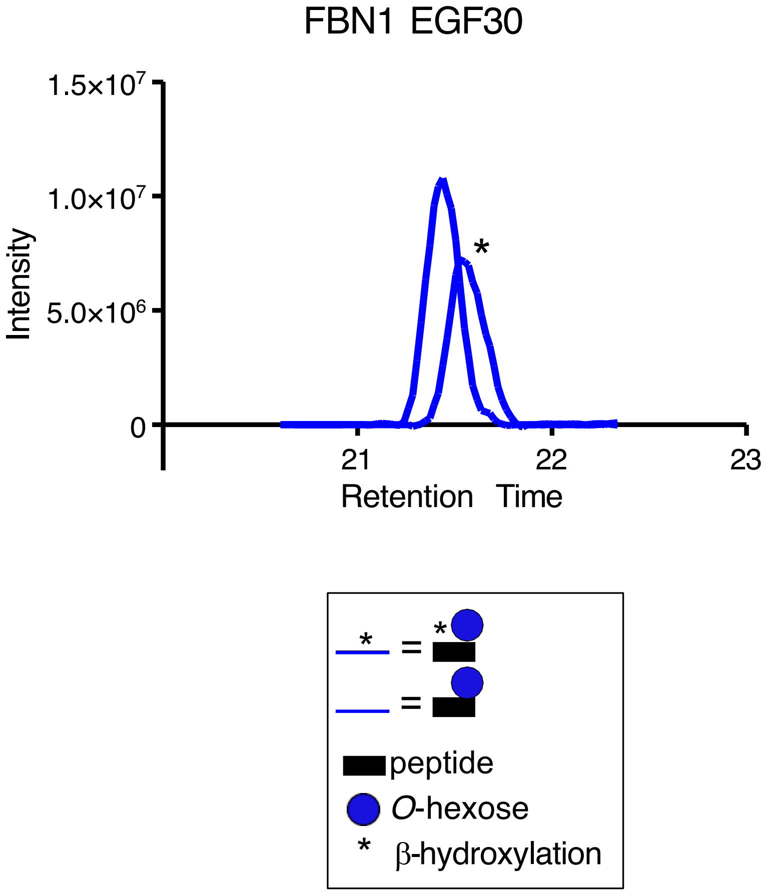
FBN1 EGF30
Intensity
Retention Time
peptide
O-hexose
β-hydroxylation

FBN1 EGF 31

b 2 3 4 5 6 7 9 10 11 12 13 15 16

LTSTGQCNDRNECQEIPNICSHGQCIDTVGSFYCLCHTGFK, Charge 5

12 10 9 8 7 6 5 4 3 2 1 y

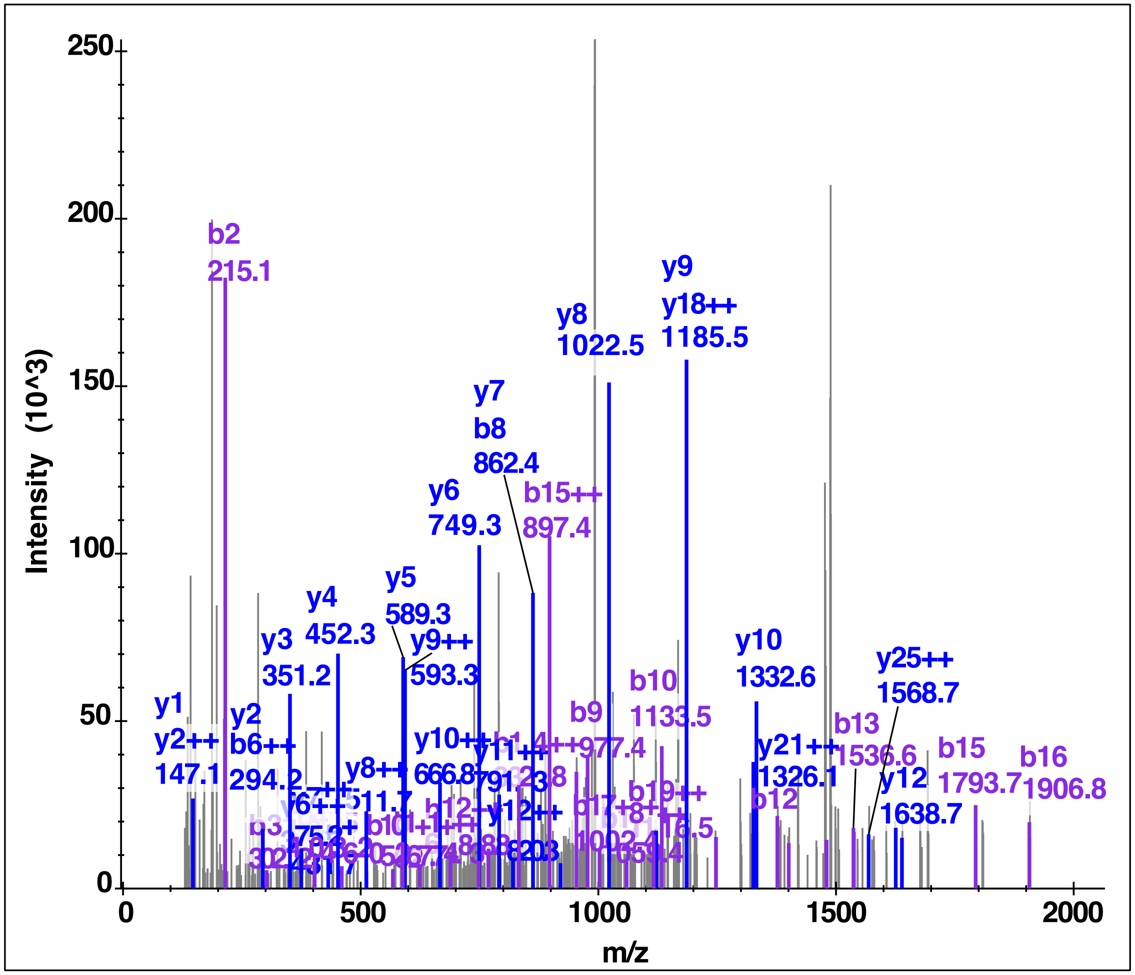

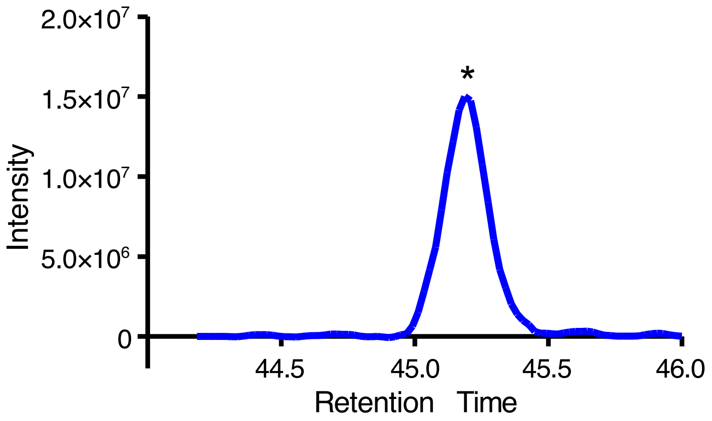

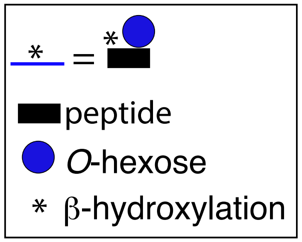

FBN1 EGF 32

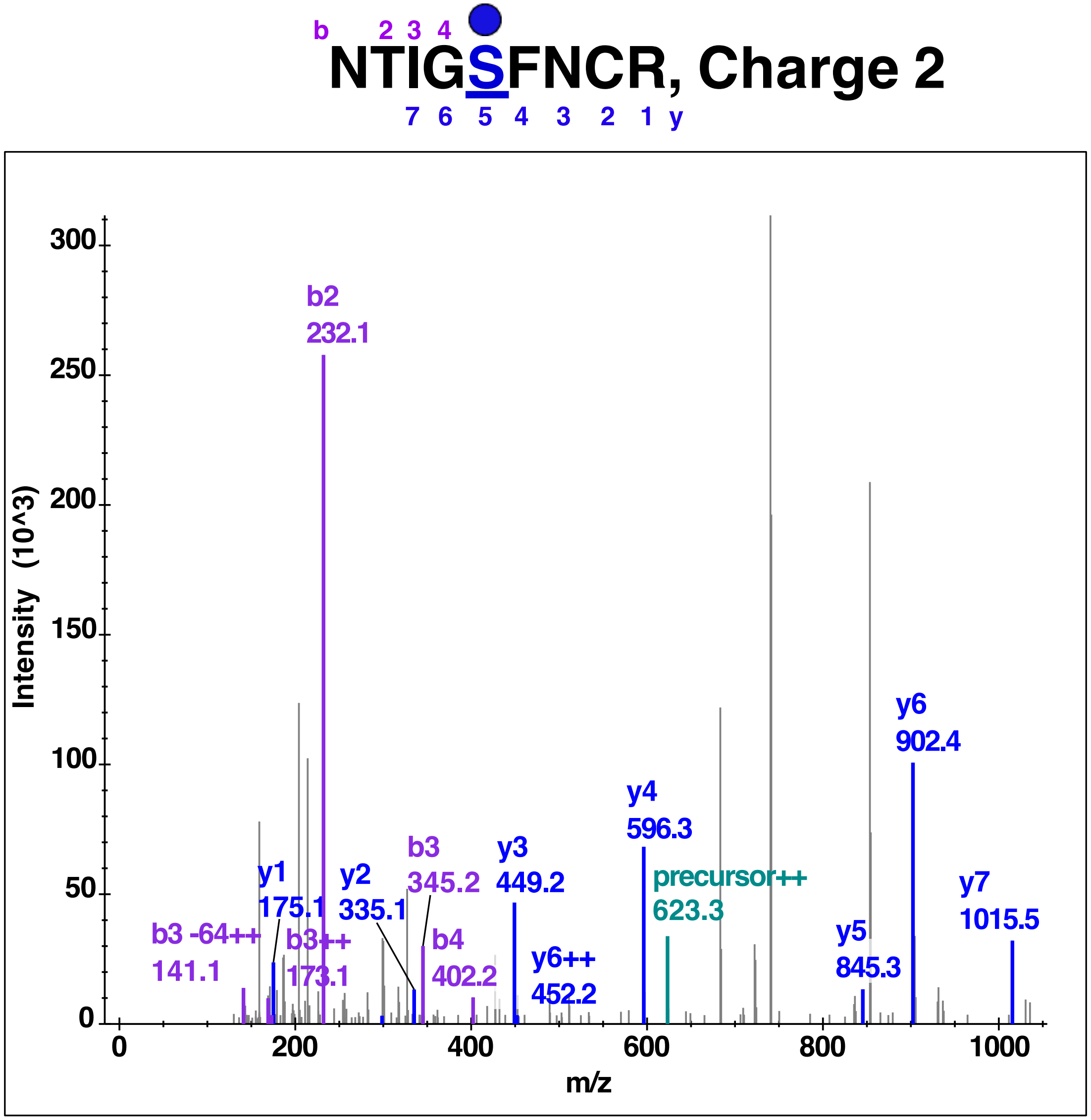

NTIGSFNCR, Charge 2
b 2 3 4
7 6 5 4 3 2 1 y
Intensity (10^3)
m/z
b2 232.1
b3 345.2
b4 402.2
b3 -64++ 141.1
b3++ 173.1
y1 175.1
y2 335.1
y3 449.2
y4 596.3
y5 845.3
y6 902.4
y6++ 452.2
y7 1015.5
precursor++ 623.3

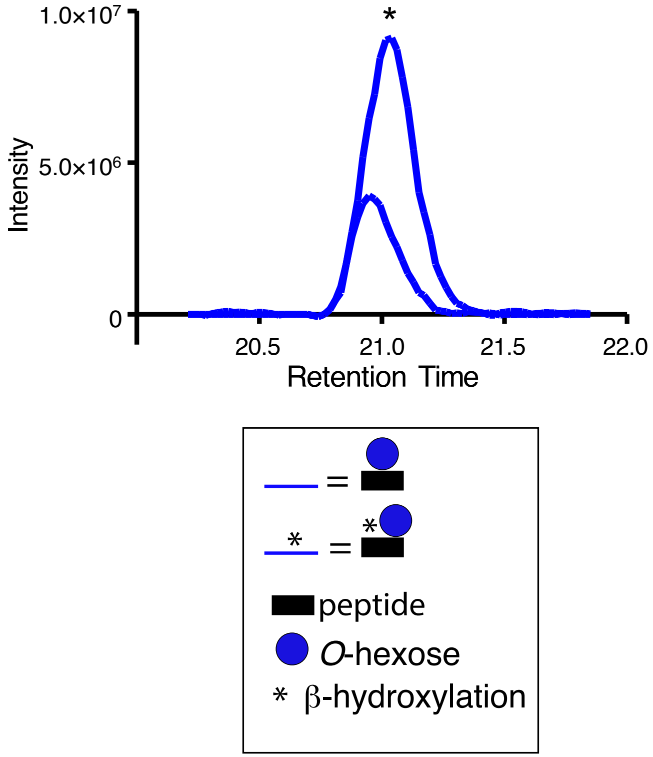

Intensity
Retention Time
peptide
O-hexose
* β-hydroxylation

FBN1 EGF 36

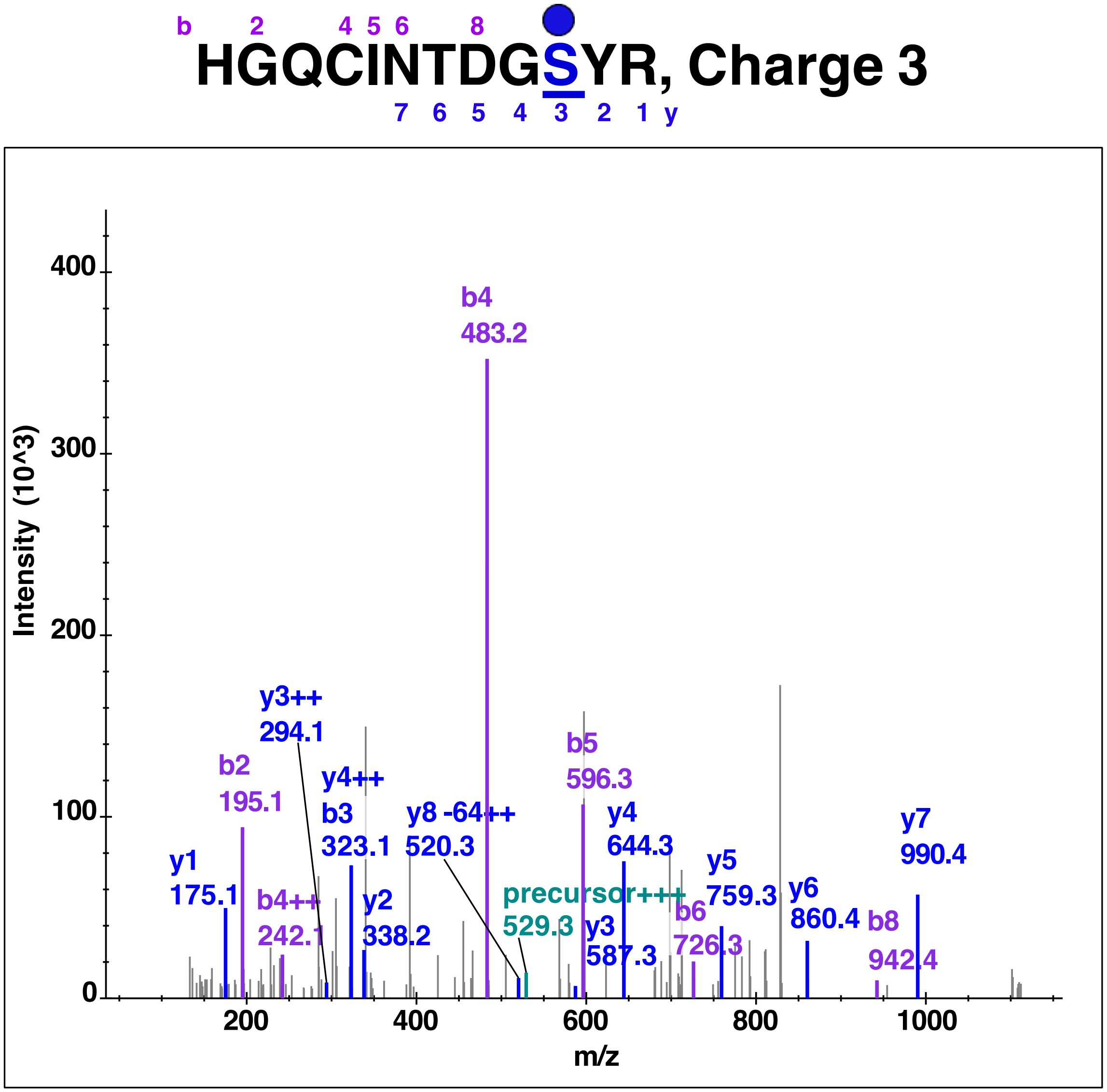

b 2 4 5 6 8
HGQCINTDGSYR, Charge 3
7 6 5 4 3 2 1 y
Intensity (10^3)
m/z
b4
483.2
y3++
294.1
b2
195.1
y4++
b3
323.1
y8 -64++
520.3
b5
596.3
y4
644.3
y7
990.4
y1
175.1
b4++
242.1
y2
338.2
precursor+++
529.3
y3
587.3
b6
726.3
y5
759.3
y6
860.4
b8
942.4

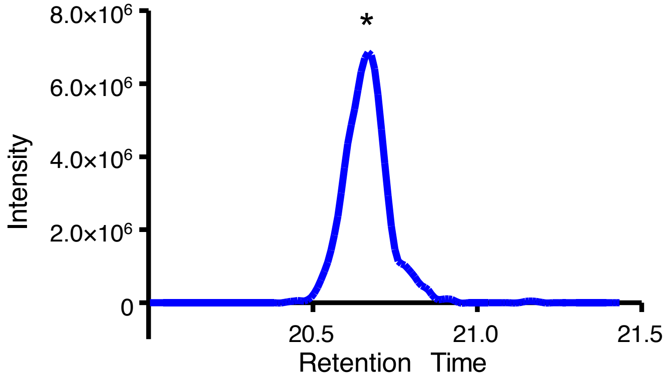

Intensity
Retention Time
*

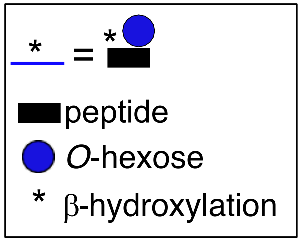

* = *
peptide
O-hexose
* β-hydroxylation

FBN1 EGF 42

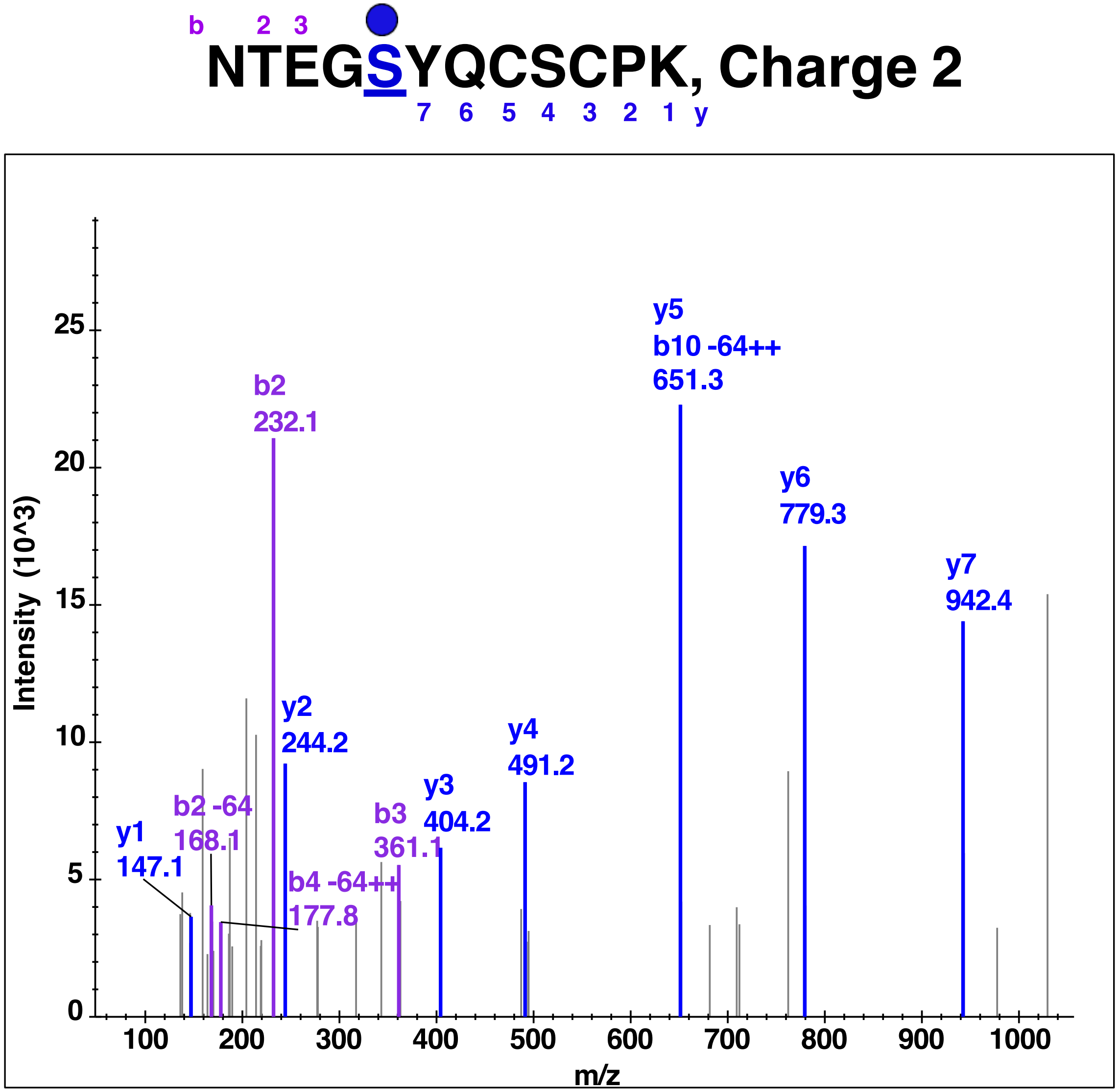

b 2 3
NTEGSYQCSCPK, Charge 2
7 6 5 4 3 2 1 y
Intensity (10^3)
m/z
y5
b10 -64++
651.3
b2
232.1
y6
779.3
y7
942.4
y2
244.2
y4
491.2
y3
404.2
b3
361.1
b2 -64
168.1
y1
147.1
b4 -64++
177.8

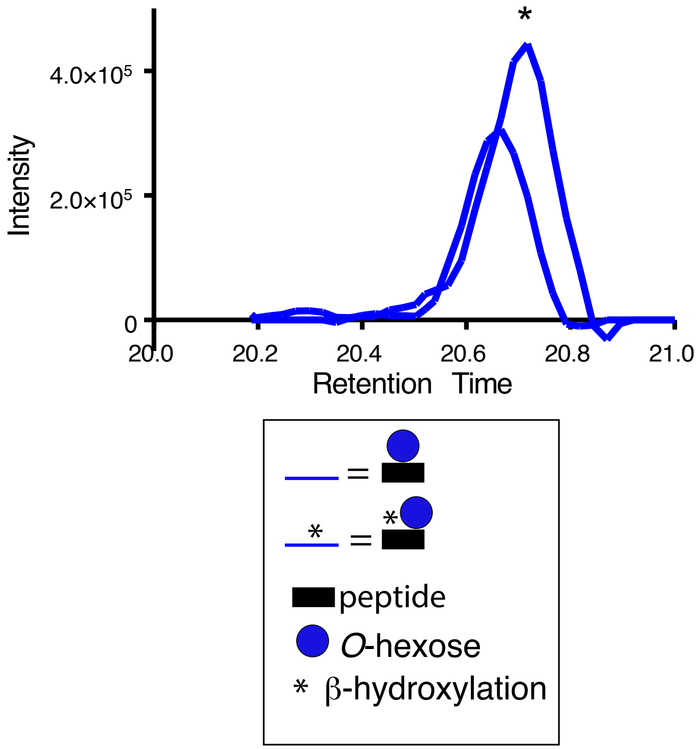

Intensity
Retention Time
peptide
O-hexose
* β-hydroxylation

FBN1 EGF 44

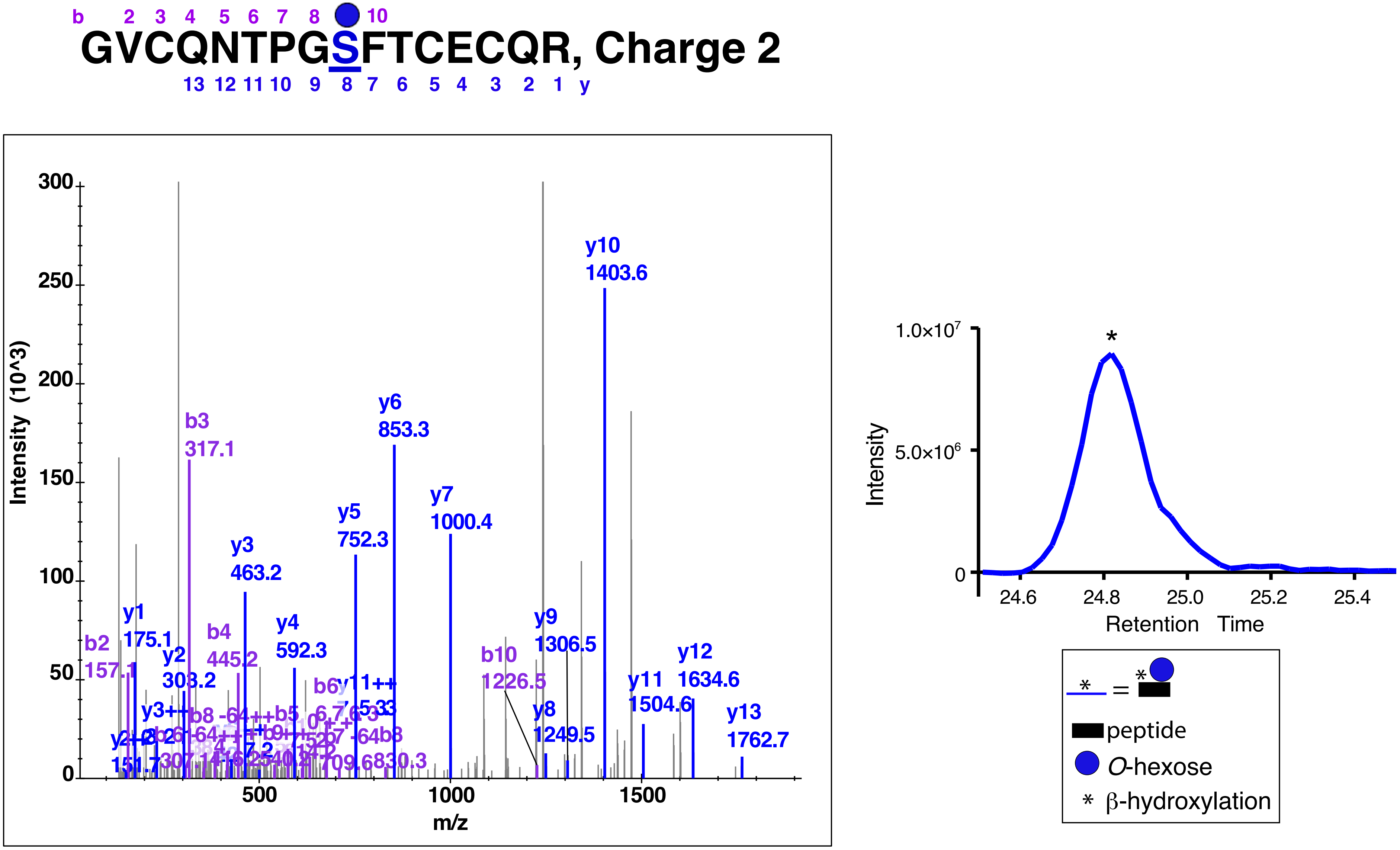

GVCQNTPGSFTCECQR, Charge 2
y10 1403.6
y6 853.3
b3 317.1
y7 1000.4
y5 752.3
y3 463.2
y1 175.1
b4 445.2
y4 592.3
b2 157.1
y2 303.2
b10 1226.5
y9 1306.5
y12 1634.6
y11 1504.6
y8 1249.5
y13 1762.7
Intensity (10^3)
m/z
Intensity
Retention Time
peptide
O-hexose
β-hydroxylation

FBLN2 EGF 4

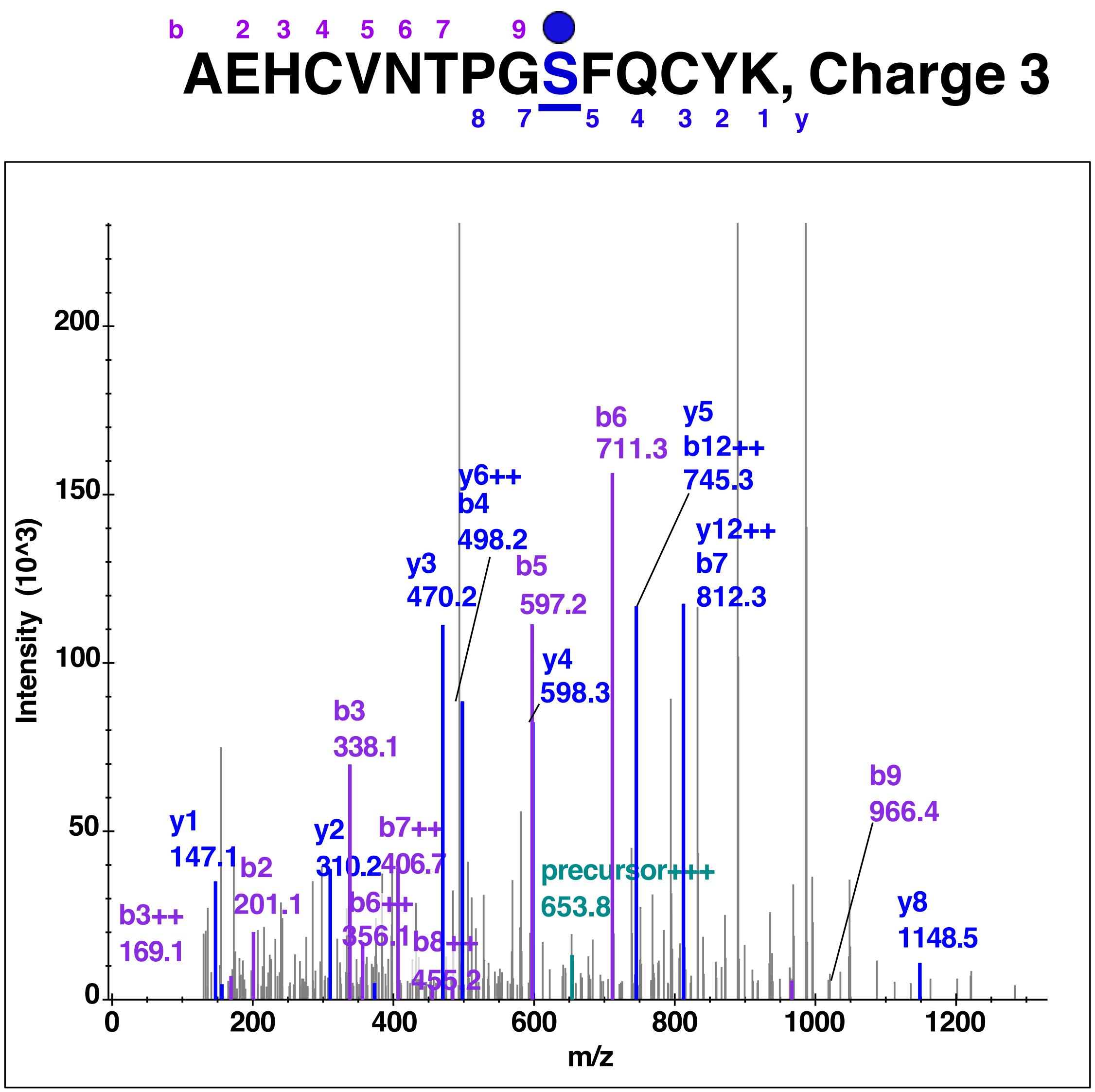

b 2 3 4 5 6 7 9
AEHCVNTPGSFQCYK, Charge 3
8 7 5 4 3 2 1 y
Intensity (10^3)
m/z
y1 147.1
b2 201.1
b3++ 169.1
y2 310.2
b3 338.1
b7++ 406.7
b6++ 356.1
b8++ 455.2
y3 470.2
y6++ b4 498.2
b5 597.2
y4 598.3
precursor+++ 653.8
b6 711.3
y5 b12++ 745.3
y12++ b7 812.3
b9 966.4
y8 1148.5

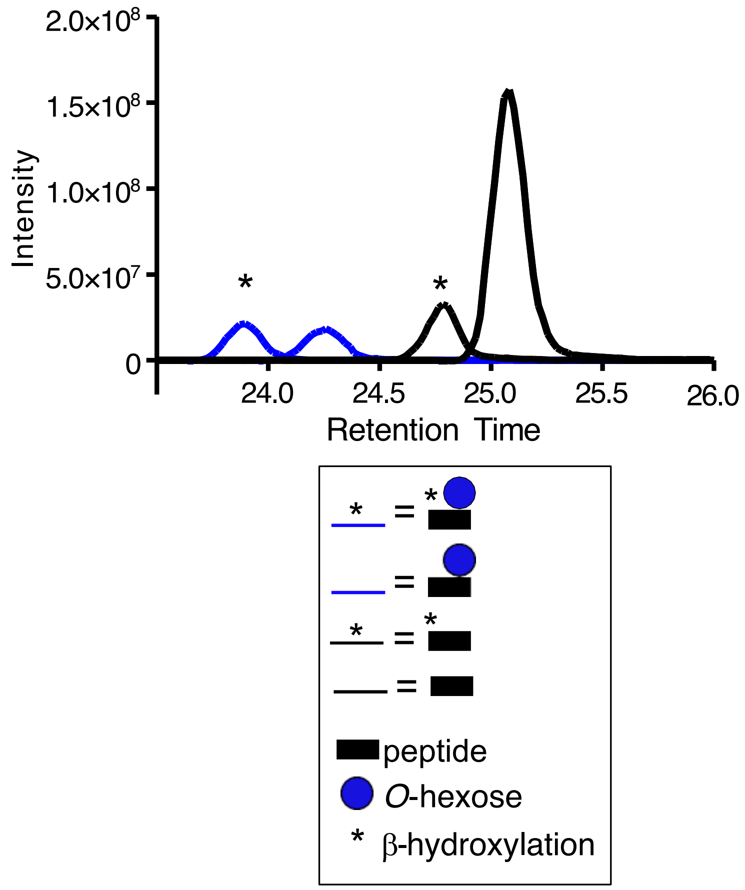

Intensity
Retention Time
*
*
peptide
O-hexose
* β-hydroxylation

FBLN2 EGF 6

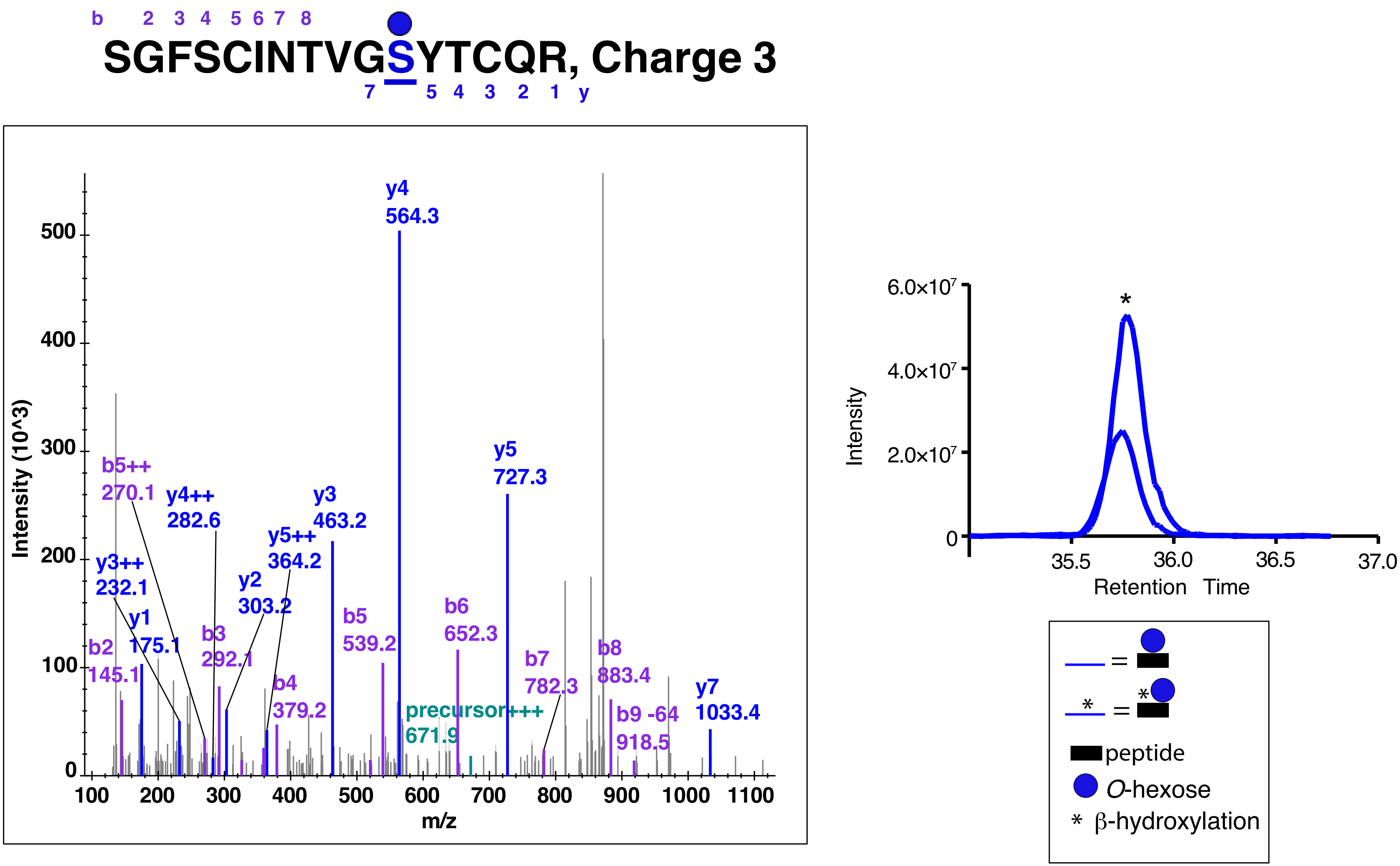

b 2 3 4 5 6 7 8
SGFSCINTVGSYTCQR, Charge 3
7 5 4 3 2 1 y
Intensity (10^3)
m/z
y4 564.3
y5 727.3
y3 463.2
y5++ 364.2
y4++ 282.6
b5++ 270.1
y3++ 232.1
y2 303.2
y1 175.1
b2 145.1
b3 292.1
b4 379.2
b5 539.2
b6 652.3
precursor+++ 671.9
b7 782.3
b8 883.4
b9 -64 918.5
y7 1033.4
Intensity
Retention Time
6.0×10^7
4.0×10^7
2.0×10^7
0
35.5
36.0
36.5
37.0
peptide
O-hexose
* β-hydroxylation

FBLN2 EGF 8

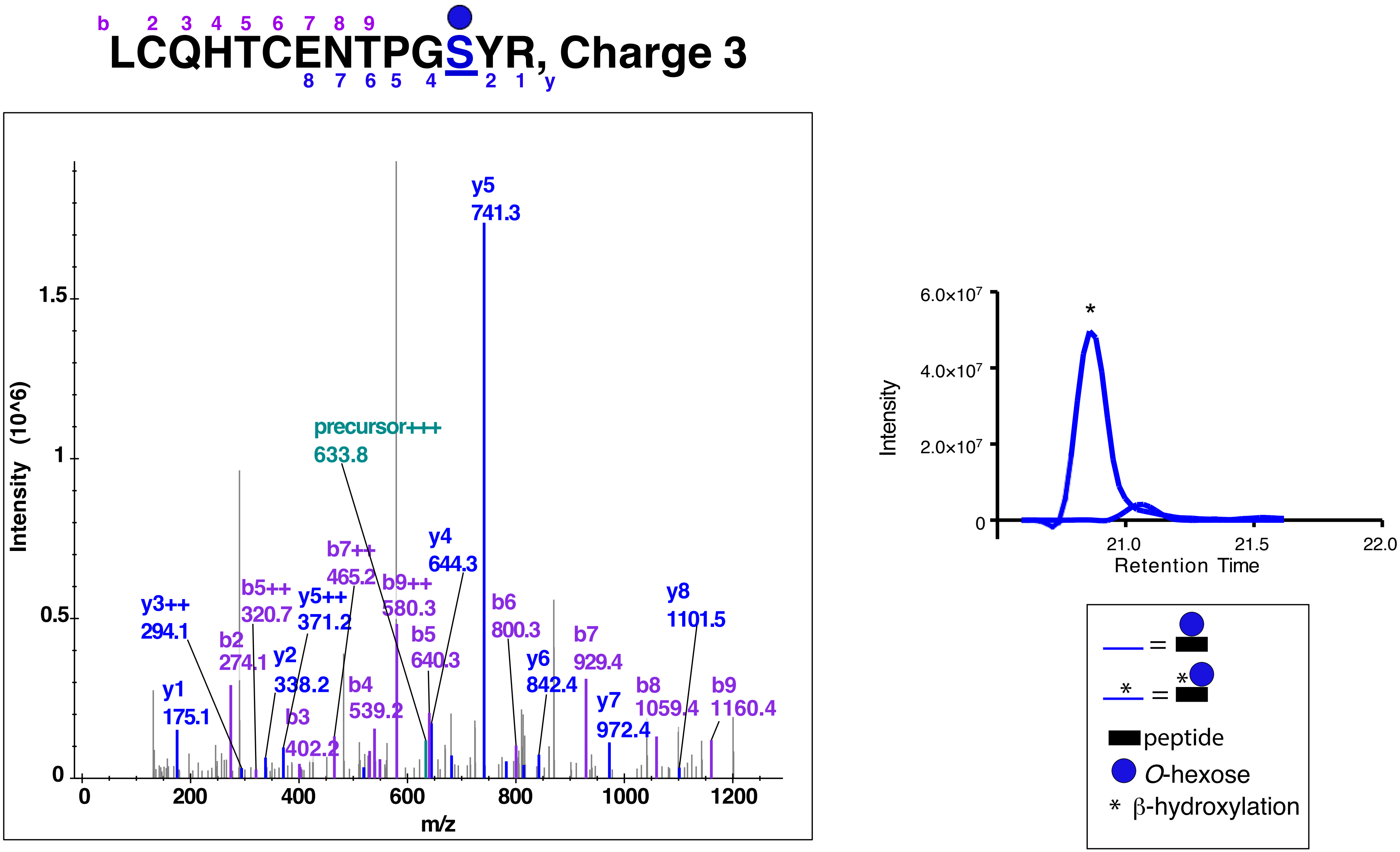

b 2 3 4 5 6 7 8 9
LCQHTCENTPGSYR, Charge 3
8 7 6 5 4 2 1 y
Intensity (10^6)
m/z
y5 741.3
precursor+++ 633.8
y4 644.3
b7++ 465.2
b9++ 580.3
b5++ 320.7
y5++ 371.2
y3++ 294.1
b2 274.1
y2 338.2
b3 402.2
b4 539.2
b5 640.3
b6 800.3
y6 842.4
b7 929.4
y7 972.4
y8 1101.5
b8 1059.4
b9 1160.4
y1 175.1
Intensity
Retention Time
= peptide
O-hexose
* β-hydroxylation

FBLN2 EGF 9

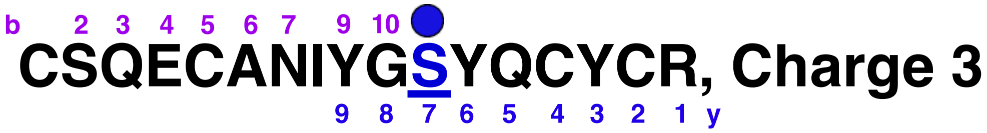

b 2 3 4 5 6 7 9 10
CSQECANIYGSYQCYCR, Charge 3
9 8 7 6 5 4 3 2 1 y

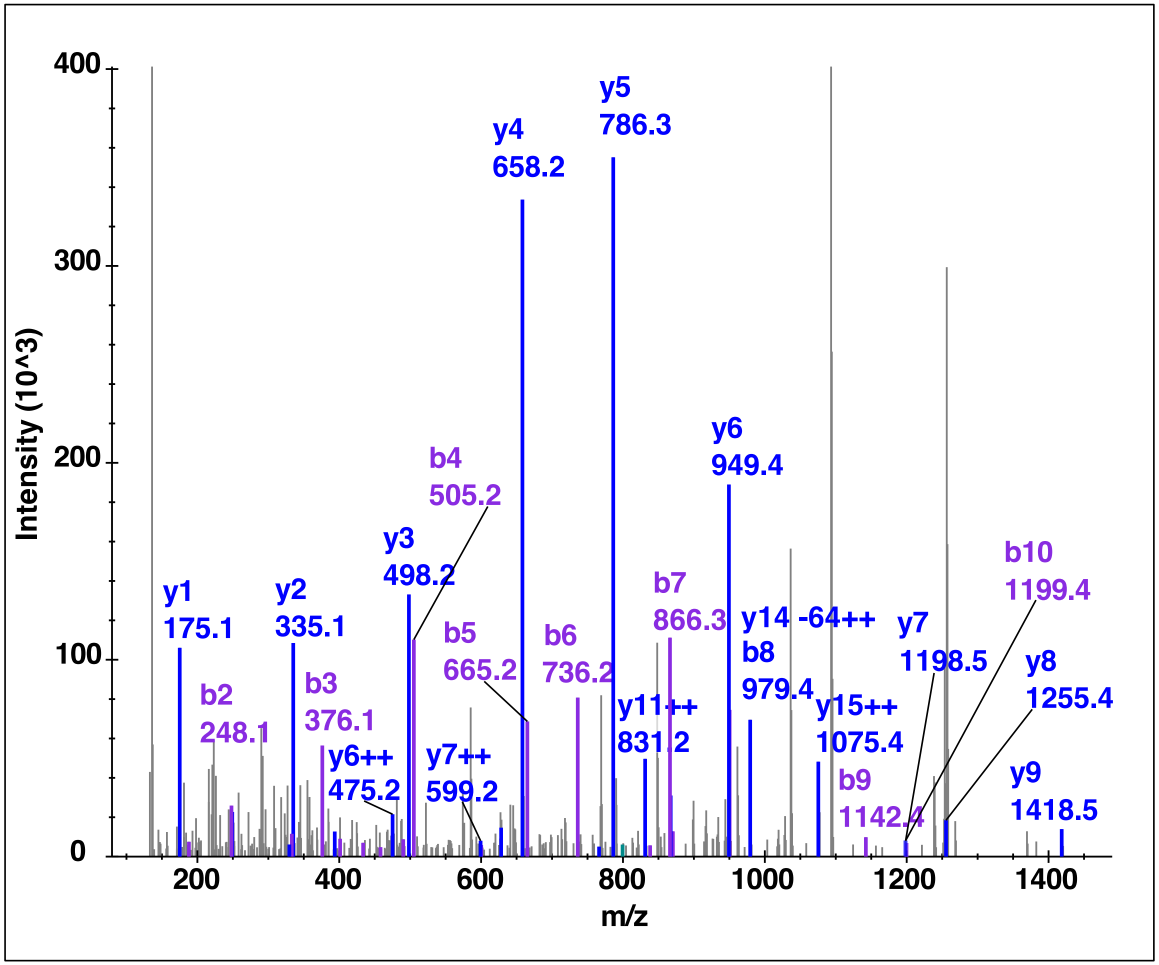

Intensity (10^3)
400
300
200
100
0
200
400
600
800
1000
1200
1400
m/z
y1 175.1
y2 335.1
b2 248.1
b3 376.1
y3 498.2
b4 505.2
y4 658.2
y5 786.3
y6++ 475.2
y7++ 599.2
b5 665.2
b6 736.2
y11++ 831.2
b7 866.3
y6 949.4
y14 -64++
b8 979.4
y15++ 1075.4
b9 1142.4
y7 1198.5
b10 1199.4
y8 1255.4
y9 1418.5

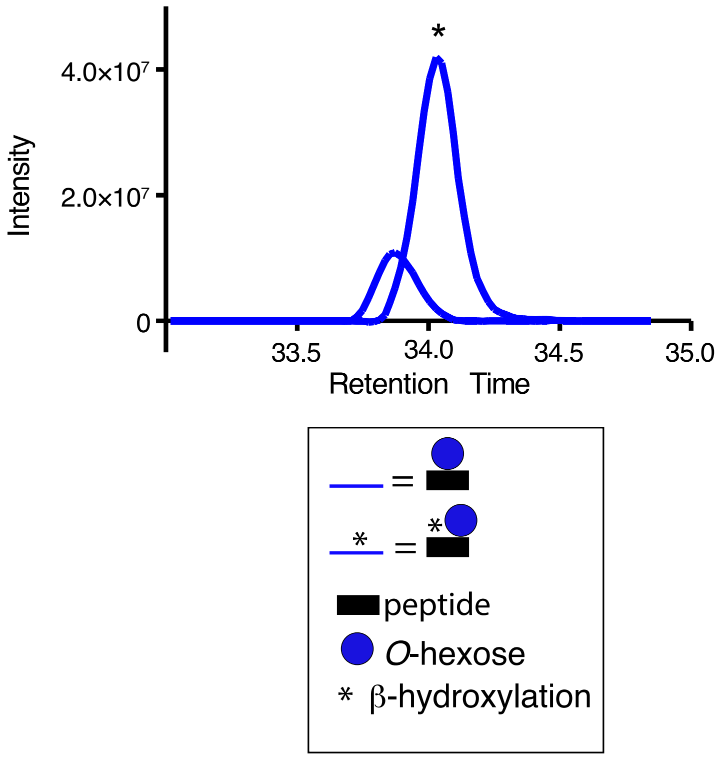

Intensity
4.0×10^7
2.0×10^7
0
33.5
34.0
34.5
35.0
Retention Time
*
=
* =
peptide
O-hexose
* β-hydroxylation

FBLN2 EGF 10

b 2 3 4
CVNVPGSYQCACPEQGYTMMANGR, Charge 3
13 12 11 10 9 8 7 6 5 4 3 2 1 y

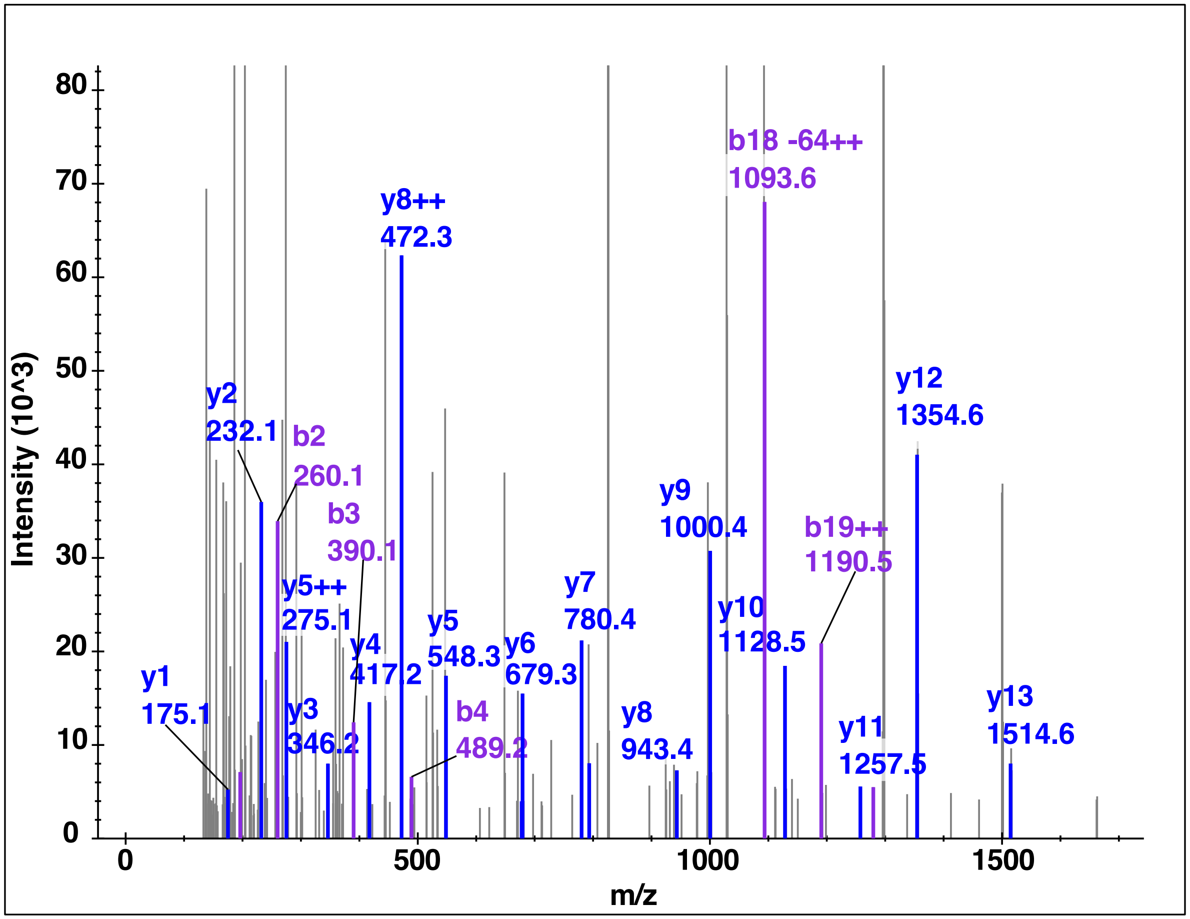

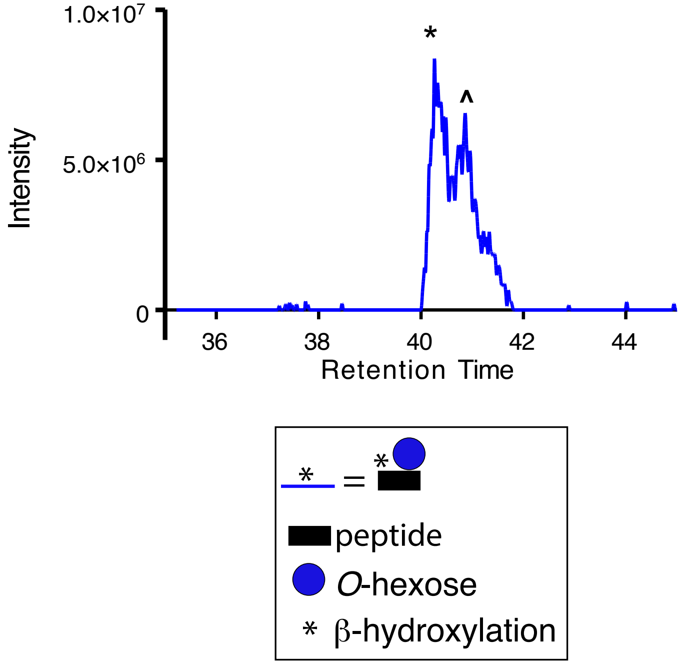

^different co-eluting peptide

FBLN5 EGF 3

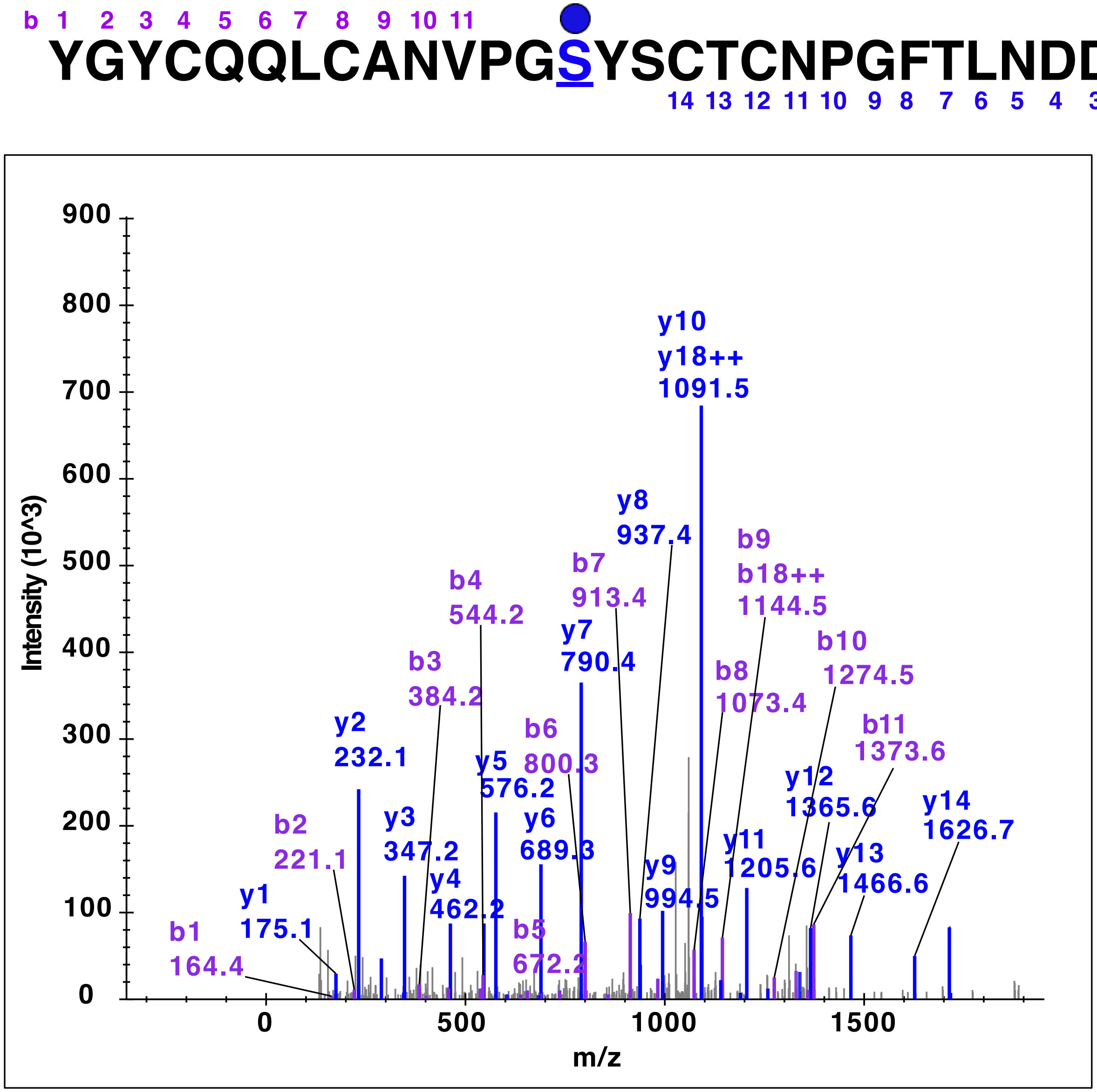
b 1 2 3 4 5 6 7 8 9 10 11
YGYCQQLCANVPGSYSCTCNPGFTLNDDGR, Charge 3
14 13 12 11 10 9 8 7 6 5 4 3 2 1 y
Intensity (10^3)
m/z
y10
y18++
1091.5
y8
937.4
b9
b18++
1144.5
b4
544.2
b7
913.4
y7
790.4
b10
1274.5
b3
384.2
b8
1073.4
y2
232.1
b6
800.3
b11
1373.6
y5
576.2
y12
1365.6
y14
1626.7
b2
221.1
y3
347.2
y6
689.3
y11
1205.6
y13
1466.6
y4
462.2
y9
994.5
y1
175.1
b1
164.4
b5
672.2

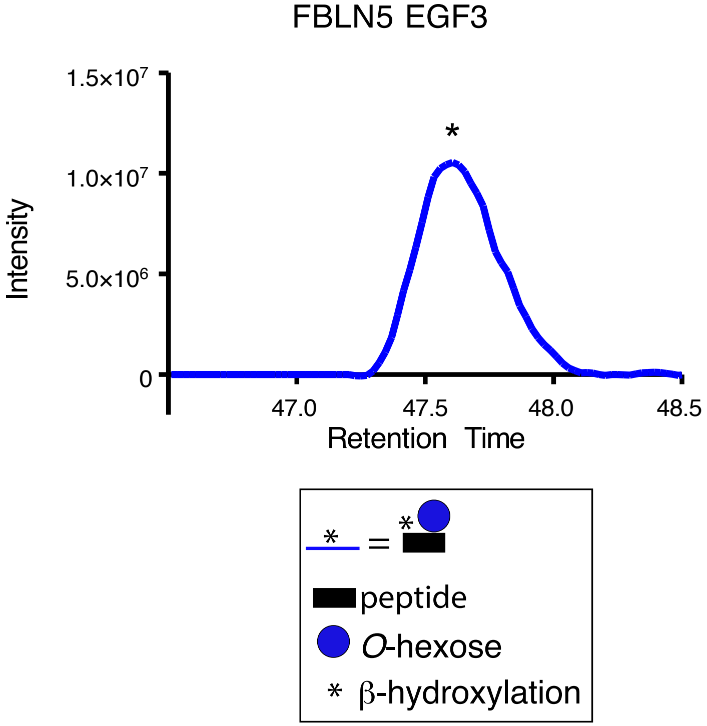
FBLN5 EGF3
Intensity
Retention Time
*
peptide
O-hexose
* β-hydroxylation

NID1 EGF 5

b 2 3 4 5 6 7 8 9 10

CHPDAFCYNTPGSFTCQCKPGYQGDGFR, Charge 4

13 12 11 10 9 8 7 6 5 4 3 2 1 y

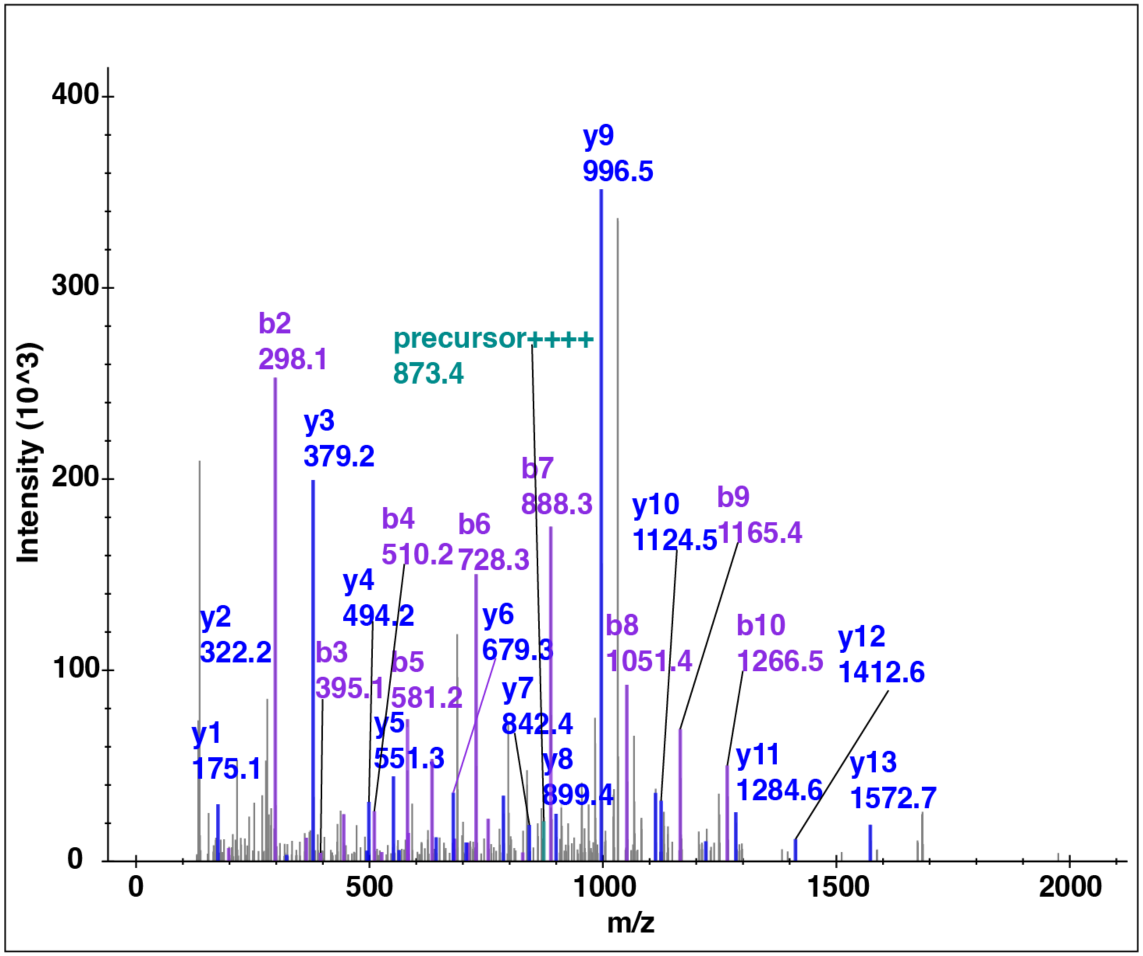

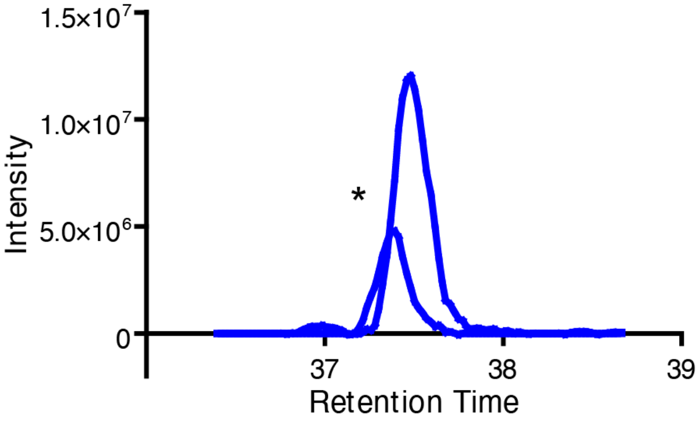

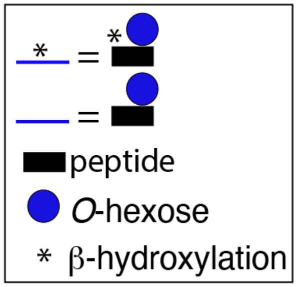

FBN1 POGLUT1 EGF 13

TCEDIDECESSPCINGVCK, Charge 3

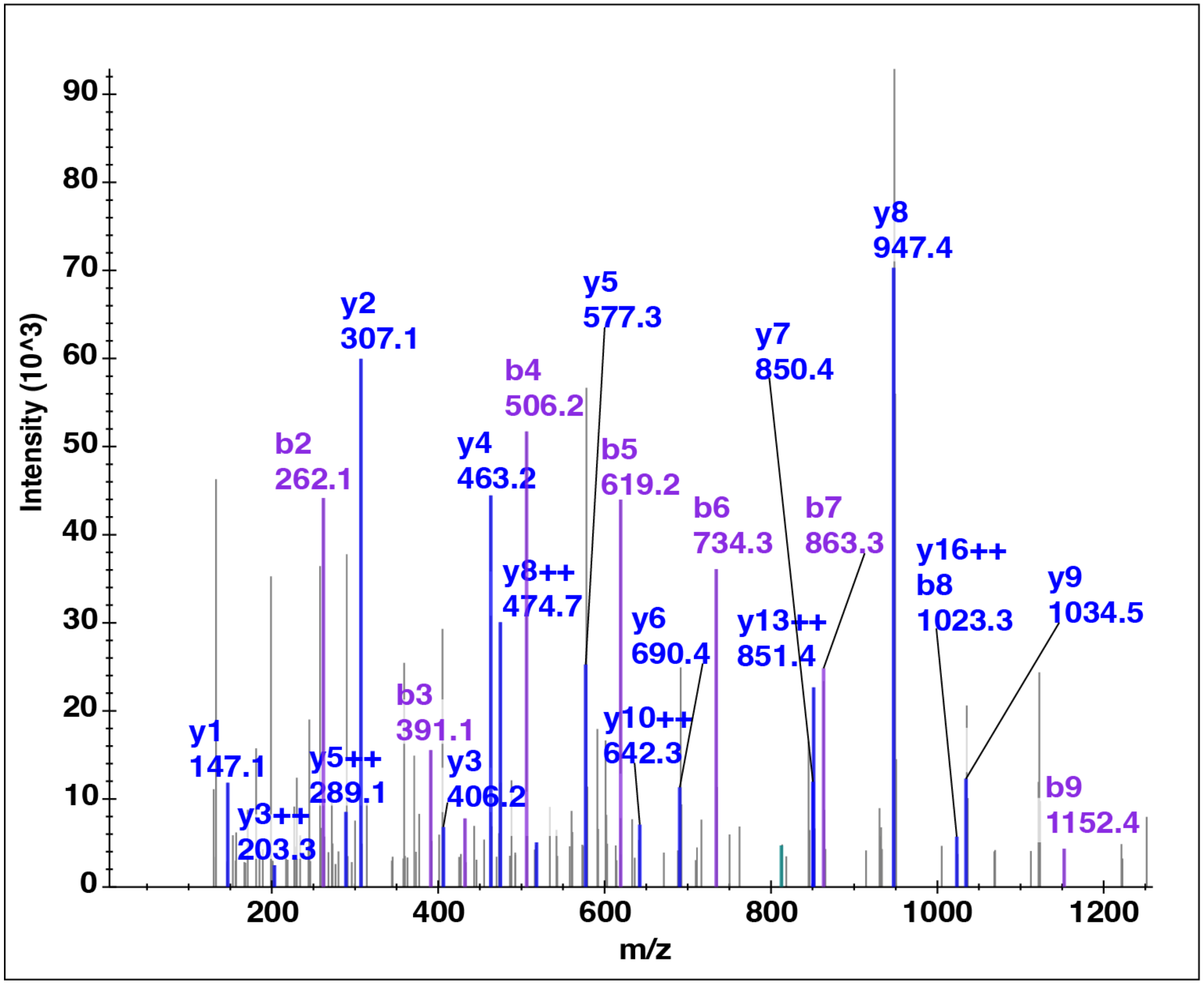

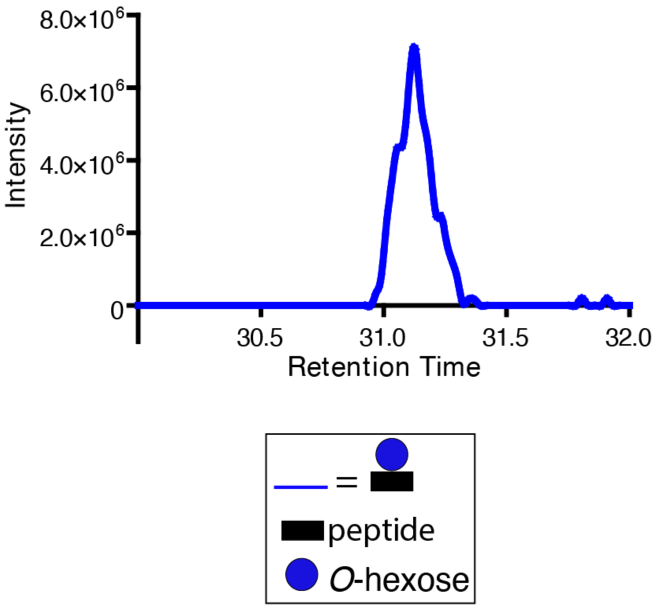

FBN1 POFUT1 EGF 2

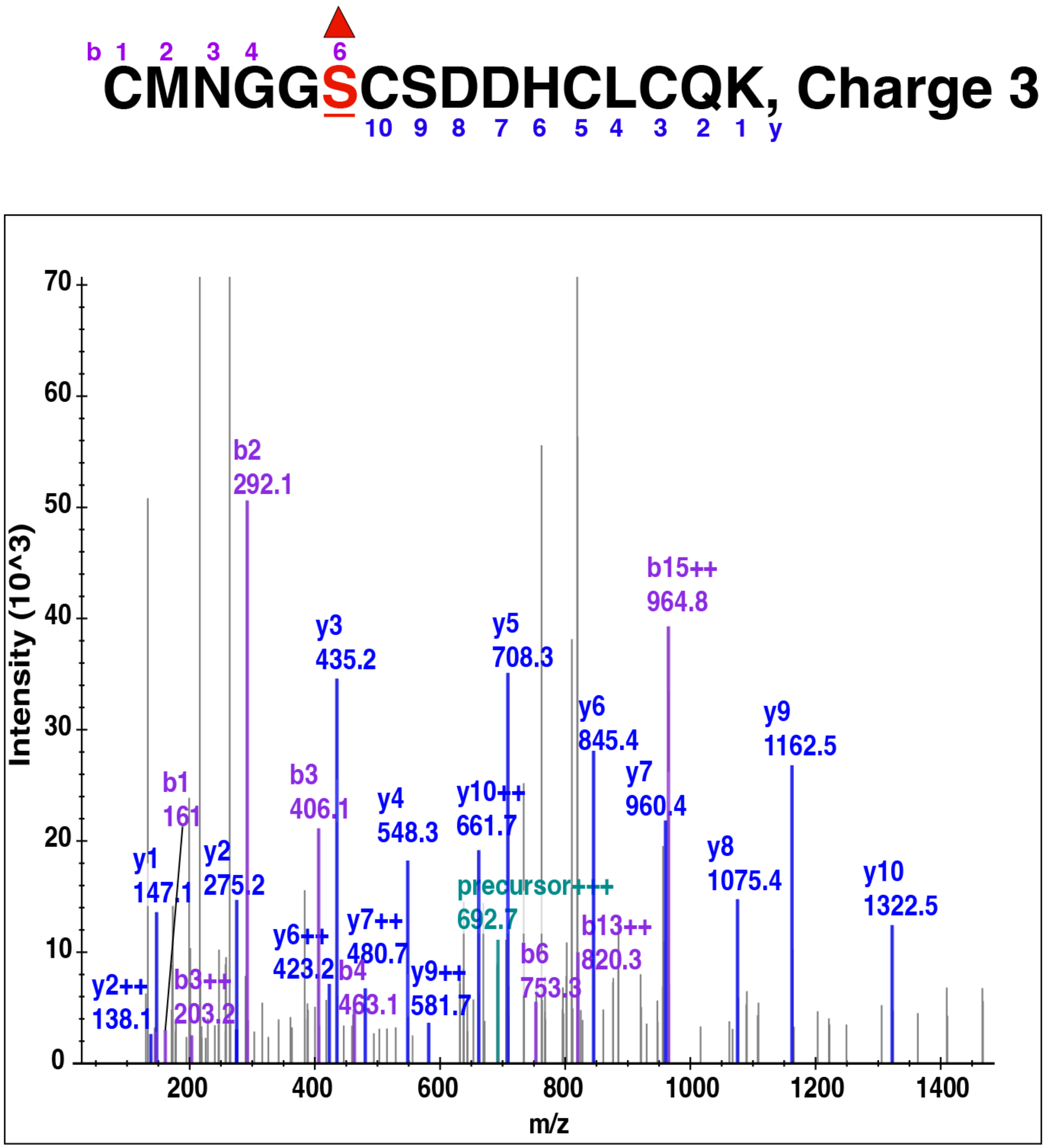

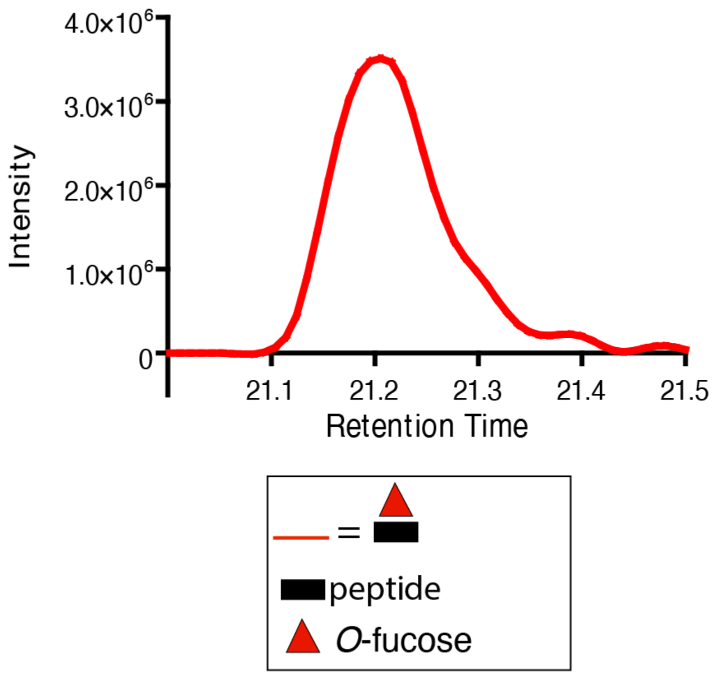

FBN1 POFUT1 EGF 34

CAPGTCQNLDGSYR, Charge 2

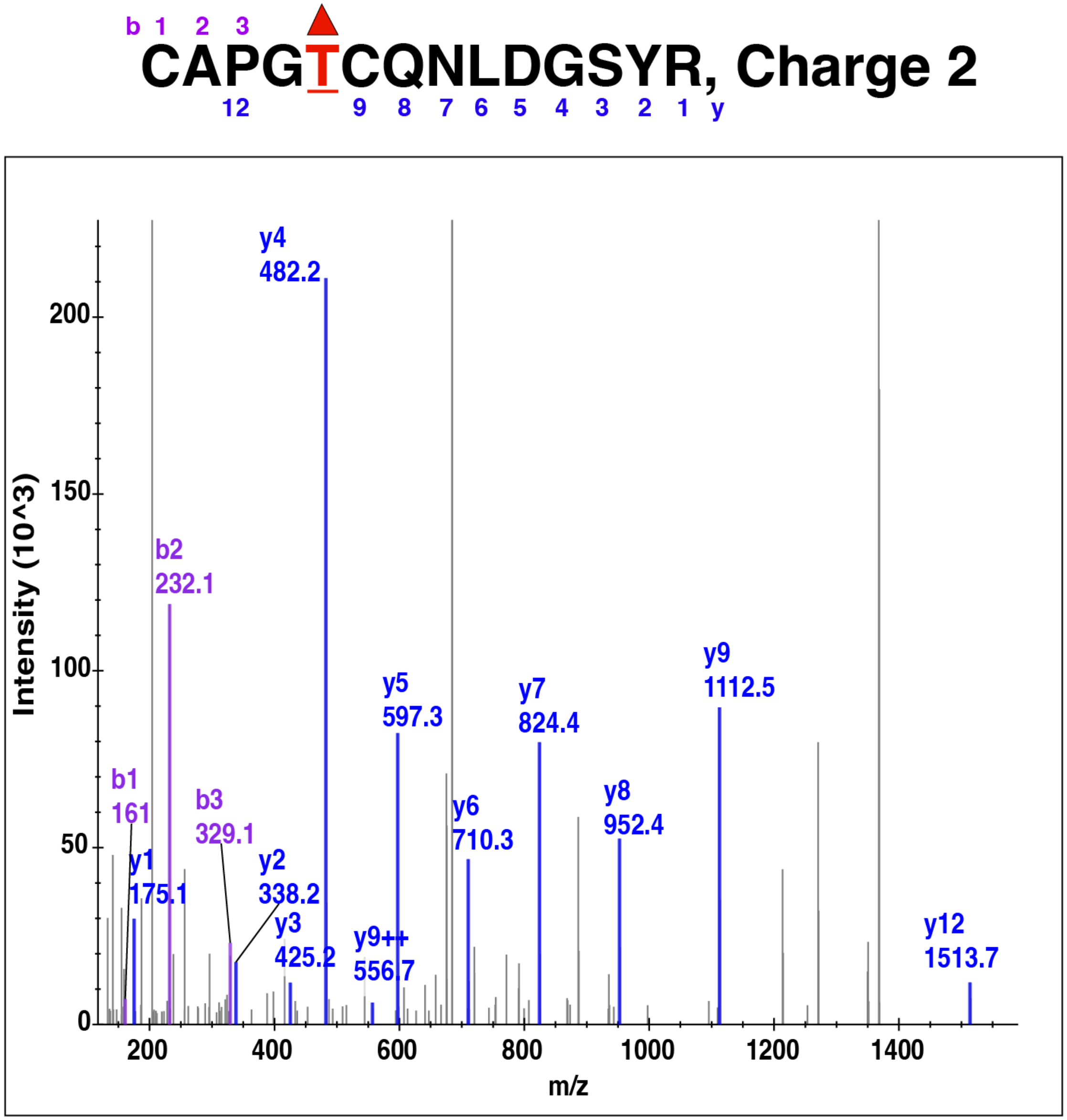

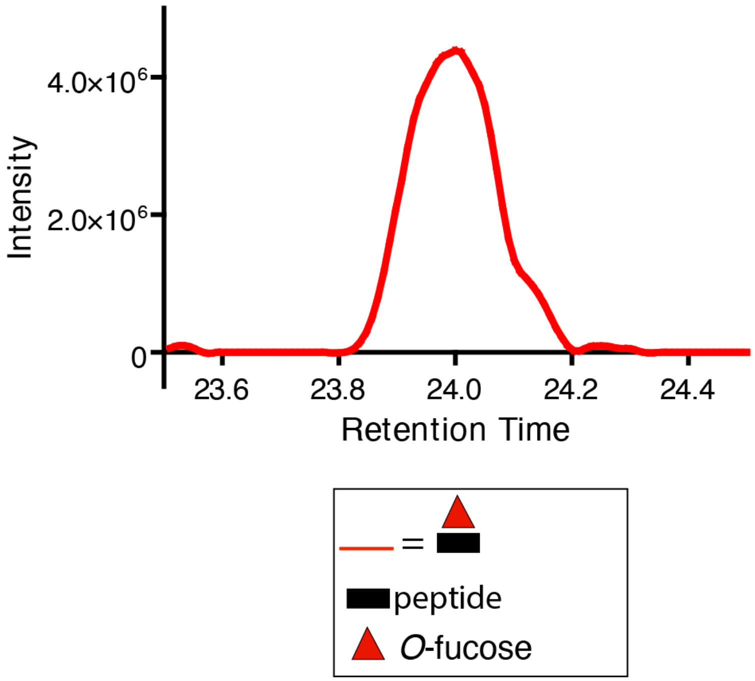

Supplement: Supporting Data [file mmc2.pdf]
