# Supplementary material for: Poglut2/3 double knockout in mice results in neonatal lethality with reduced levels of fibrillin in lung tissues
Source: J Biol Chem. 2024 Jun 4;300(7):107445. doi: 10.1016/j.jbc.2024.107445 (PMC11261140; doi:10.1016/j.jbc.2024.107445)
Supplement: Supporting Figures [file mmc3.docx]

***Poglut2/3* double knockout in mice results in neonatal lethality with reduced levels of fibrillin in lung tissues**

Sanjiv Neupane^1†^, Daniel B. Williamson^2†^, Robyn A. Roth^3^, Carmen M. Halabi^3^, Robert S. Haltiwanger^2^*, and Bernadette C. Holdener^1^*

^1^Department of Biochemistry and Cell Biology, Stony Brook University, Stony Brook, NY, 11794-5215, USA

^2^ Complex Carbohydrate Research Center, Department of Biochemistry and Molecular Biology, University of Georgia, Athens, GA 30602, USA.

^3^Department of Pediatrics, Division of Nephrology, Washington University School of Medicine, St. Louis, MO 63110, USA

^†^These authors contributed equally.

*Co-corresponding authors: rhalti@uga.edu and bernadette.holdener@stonybrook.edu

**Contents:**

Supplemental Figures S1-S7

Dataset S1 legend

Excel Files 1-4 legends

**Supplementary Figures**

**
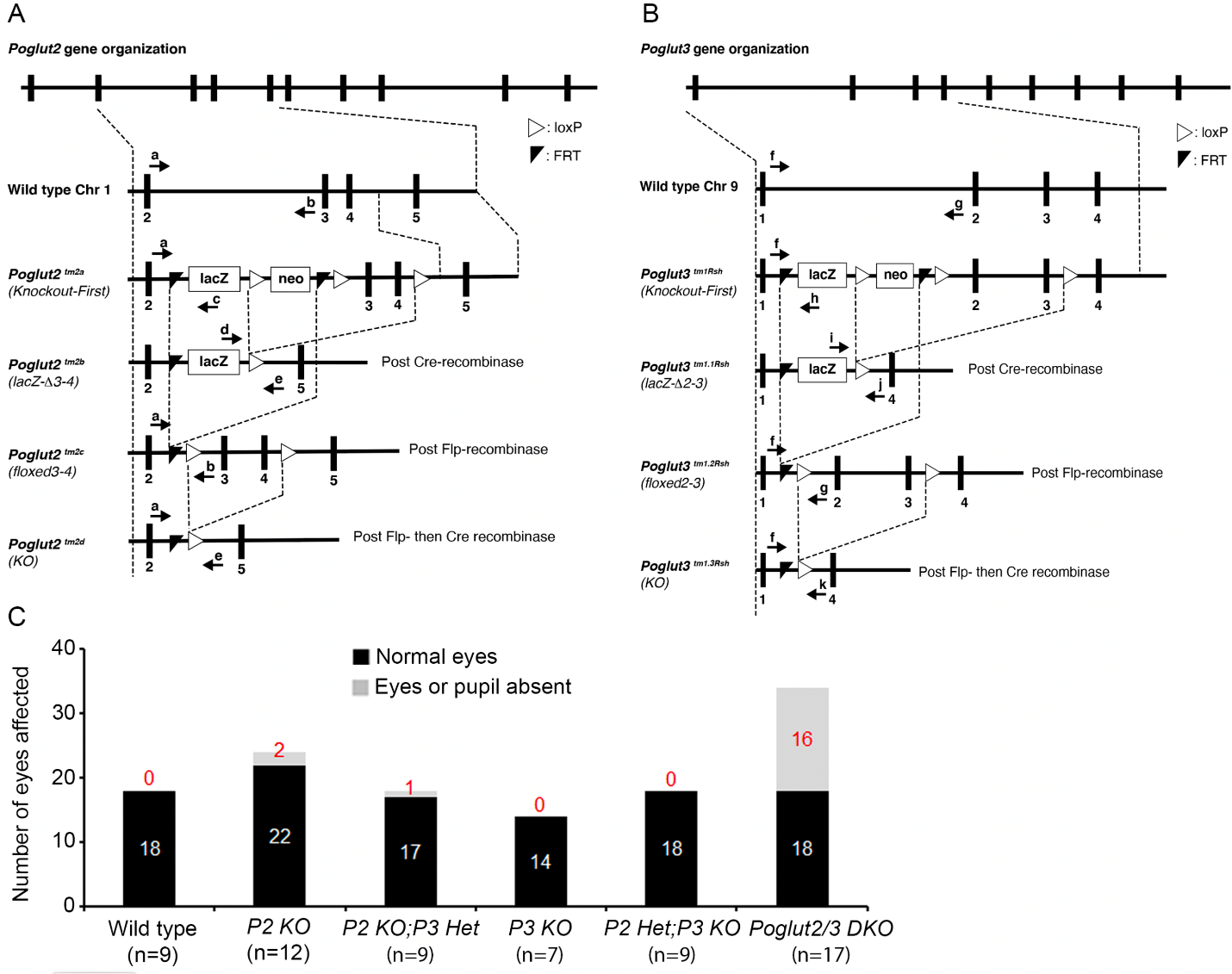
**

**Figure S1. *Poglut2* and *Poglut3* gene targeting strategy for generation of *Poglut2* and *Poglut3* knockout mice. (A-B)** Targeting strategy and locations of primers used for genotyping *Poglut2* (A) and *Poglut3* (B) alleles. Letters above arrows correspond to primers listed in Tables S1 and S2. Detailed breeding methods were described in the methods. (**C**) Incidence of eyes phenotypes at E18.5 in animals from different *Poglut2* and *Poglut3* genotypes. Abbreviations: *P2*, *Poglut2*; *P3*, *Poglut3*; *Het*, Heterozygote.


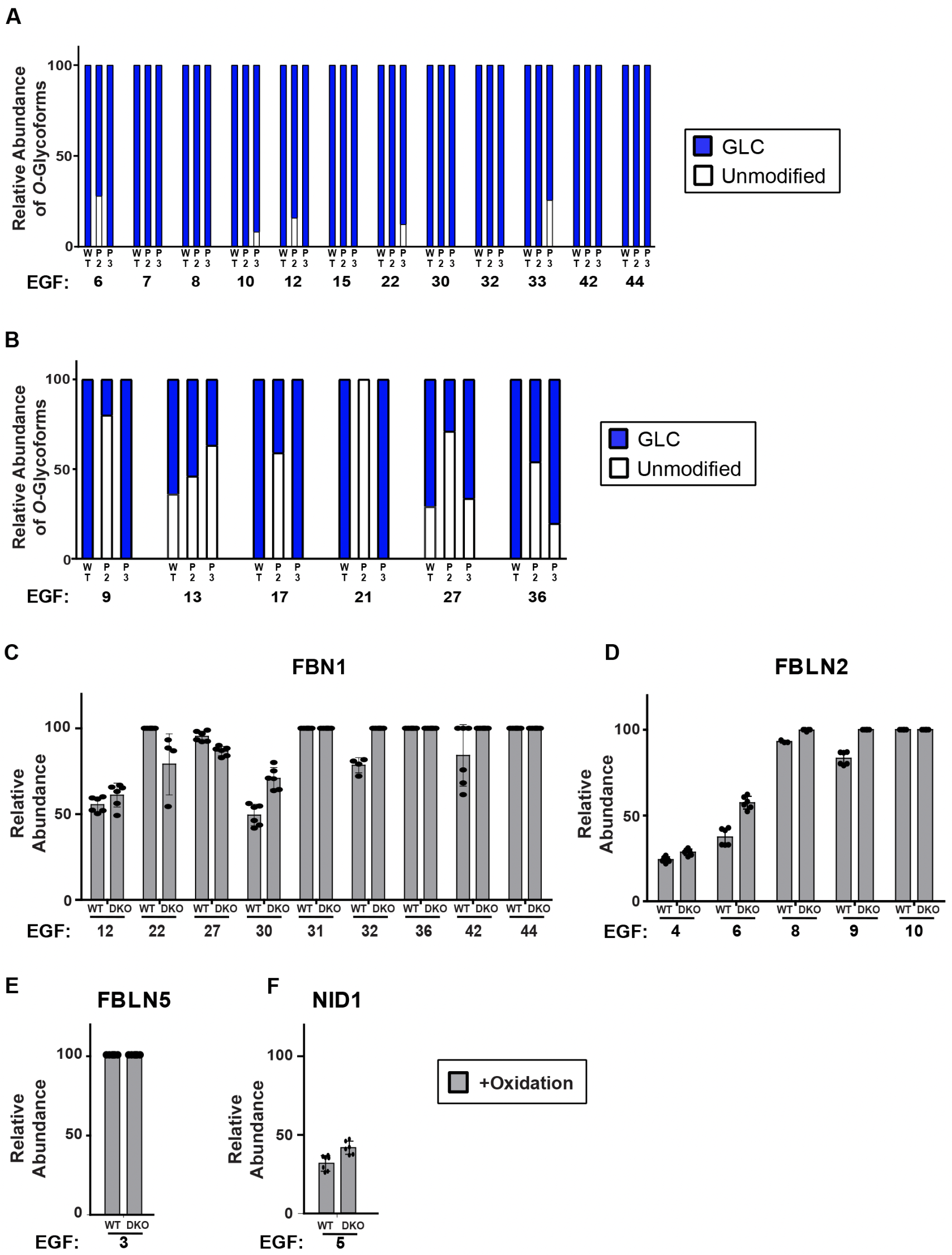


**Figure S2. POGLUT2 and 3 display some site-specificity on EGF repeats of endogenous FBN1 in *SKOs,* and β-hydroxylation of EGF repeats modified by POGLUT2/3 was not affected in *Poglut2/3 DKOs*.** **(A-B)** Relative abundance of *O*-glucosylation added by POGLUT2/3 (GLC, blue) on EGFs from endogenous, secreted FBN1 immunopurified from conditioned media of post-weaning mouse dermal fibroblasts. **(A)** EGF repeats that maintained similar levels of POGLUT2/3-mediated *O-*glucosylation in *Poglut2 KO* mice (P2) or *Poglut3 KO* mice (P3) at most sites compared to wild type (WT). **(B)** EGF repeats where deletion of either *Poglut2* (P2) or *Poglut3* (P3) severely decreased the abundance of POGLUT2/3-mediated *O-*glucosylation compared to wild type (WT). n=1 for each condition. **(C-F)** Relative amount of β-hydroxylation (oxidation) on peptides from *Poglut2/3 WT* or *DKO* EGF repeats of endogenous **(C)** FBN1, **(D)** FBLN2, **E)** FBLN5, and **(F)** NID1 secreted from lung fibroblasts. The relative abundance was calculated based on area under the curve from extracted ion chromatograms (Data set1, Excel file 1). These were plotted as a percentage of the total abundance for each peptide. Averages were taken from n=3 biological replicates. Error bars show ±SD. Abbreviations: WT, wild type; DKO, *Poglut2/3 DKO*.


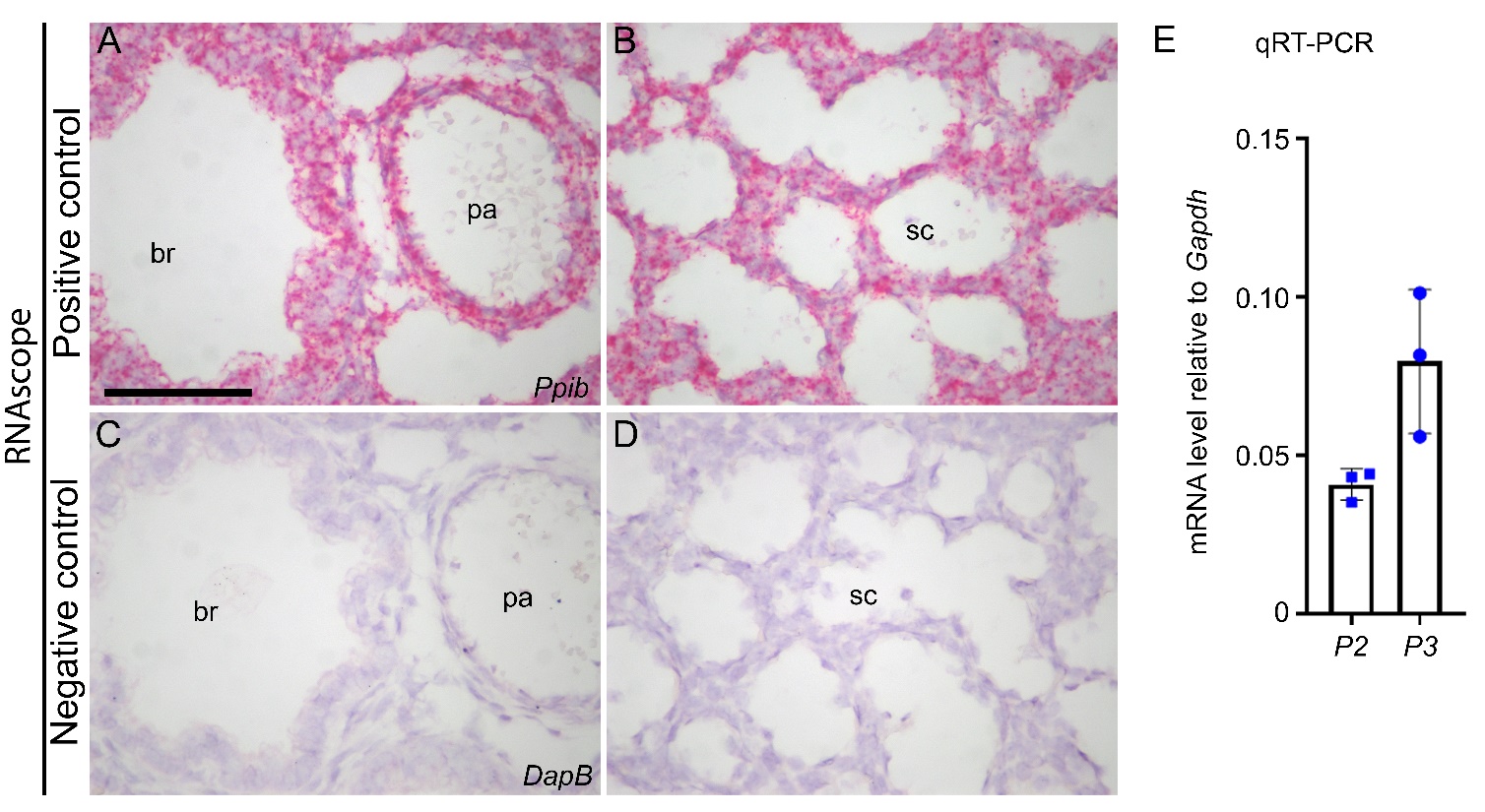


**Figure S3. RNAScope controls assay**. **(A-B)** Probes for the *Mus musculus* gene encoding peptidylprolyl isomerase B (*Ppib*) was used as a positive control and **(C-D)** the bacterial gene encoding dihydrodipicolinate reductase (*DapB*) was used as a negative control. Red dots and patches indicate positive mRNA signal. (**E**) Quantitative real time polymerase chain reaction (qRT-PCR) analysis (n=3, 3 replicates per sample) of *Poglut2* (*P2*) and *Poglut3* (*P3*) mRNA levels in E18.5 lung tissue normalized with *Gapdh*. Abbreviations: br, bronchiole; pa, pulmonary artery; sc, saccules. Scale bar for panels A-D 50 μm (represented in panel A).


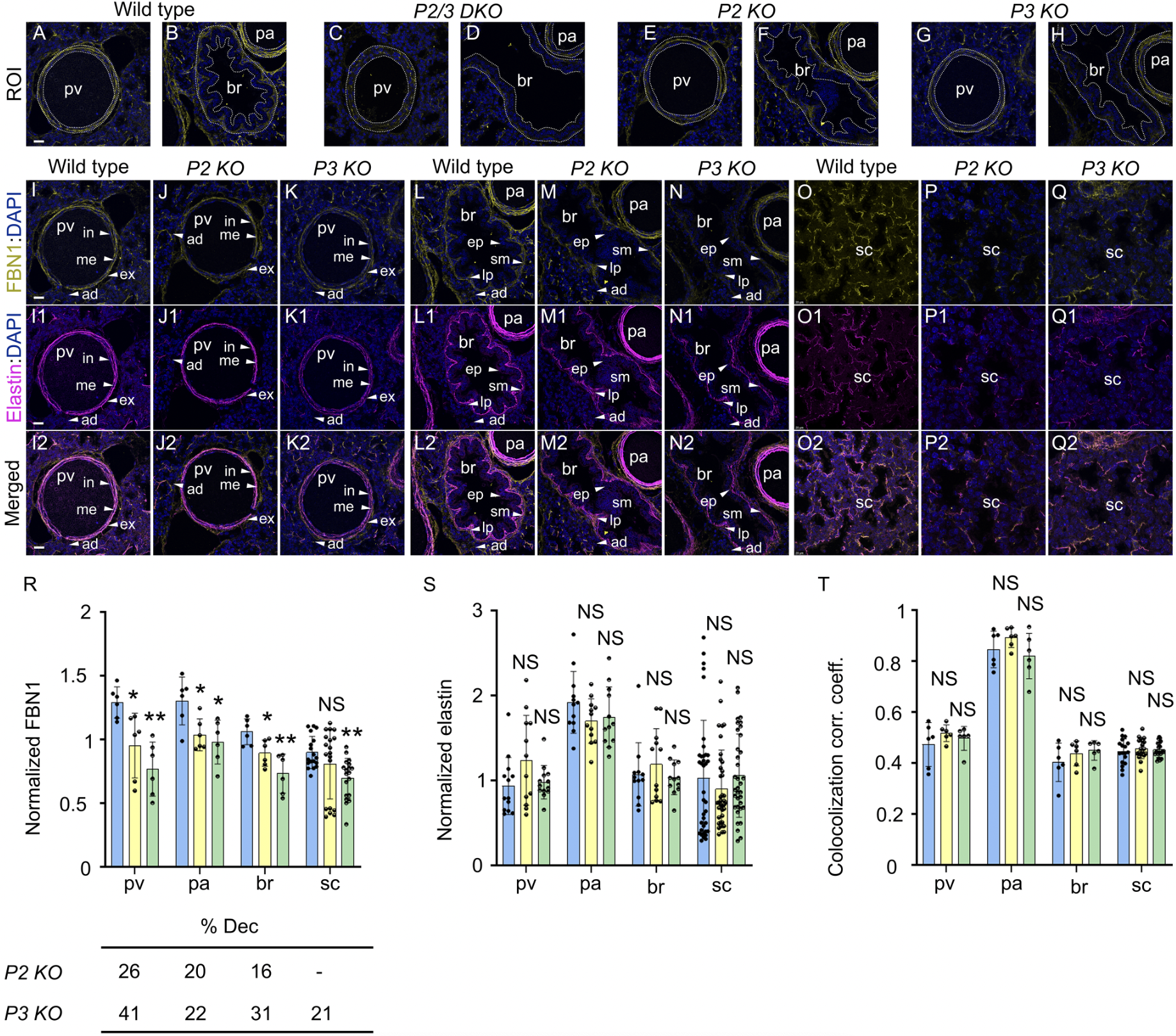


**Figure S4. Decreased FBN1 in E18.5 *Poglut2 KO (P2 KO)* and *Poglut3 KO (P3 KO)* lung.** **(A-H)** Region of interest (ROI) for immunofluorescence quantification in Figs. 4, 5, and Figs. S4, S5, and S6. For reference, the image in A is reused in I, image in B is reused in L, image in E is reused in J, image in F is reused in M, G is reused in K, and H is reused in N. Region of interest enclosed by the white dotted lines in E18.5 lung pulmonary vein (pv) and pulmonary artery (pa) and bronchiole (br) from wild type **(A-B)**, *Poglut2/3 DKO* **(C-D)**, *Poglut2 KO* (*P2 KO*) **(E-F)** and *Poglut3 KO* (*P3 KO*) **(G-H)**. **(I-Q2)** Representative maximum projection images comparing FBN1 (yellow) immunolocalization **(I-Q)**, elastin (magenta) detection using alexafluor 633 (AF633) **(I1-Q1)** with DAPI counterstaining (blue), and merged FBN1 and elastin channels showing colocalization **(I2-Q2)** in E18.5 lung sections **(I2-Q2)** merged channels of maximum projection images of FBN1 and elastin AF6333 in E18.5 lung pv, tb and saccule (sc) regions. **(R, S, T)** Quantification of FBN1 **(R)** and elastin (AF633) **(S)** signals normalized to DAPI, and estimation of colocalization correlation (corr.) coefficient (coeff.) **(T)** in the pv, pa, br, and sc regions (ROI defined in A-H). In panel (S) elastin values were pooled from all the FBN1/elastin and FBN2/elastin stained wild type, *Poglut2 KO* and *Poglut3 KO* sections, and the elastin graph in panel S includes the wild type data shown in Figs. 4 and 5. This combined graph is reused in Fig. S5K for reference. Statistically significant percent decrease (% dec) compared to wild type is indicated below graph. For comparison purposes, the wild type images in this figure (panels A, B, I, I1, I2, L, L1, L2) are reused from Fig. 4. Data from wild type (blue column with solid black circles), *Poglut2 KO* (yellow column with half-open black circles) and *Poglut3 KO* (green column with open half-black circles) were evaluated for statistical significance using unpaired, two-tailed *t*-test: *p 0.05,**p 0.01 and NS, not significant. Error bars show ± SD. Abbreviations: ad, adventitia; in, intima; me, media; ex, externa; ep, epithelium; sm, smooth muscle; lp, lamina propria. Scale bars: all panels 20 μm. Images were obtained from 3 embryos per genotype, 2-3 sections per embryo, and for pv, pa and br regions 1 field per section and for sc region 2-3 fields per sections.


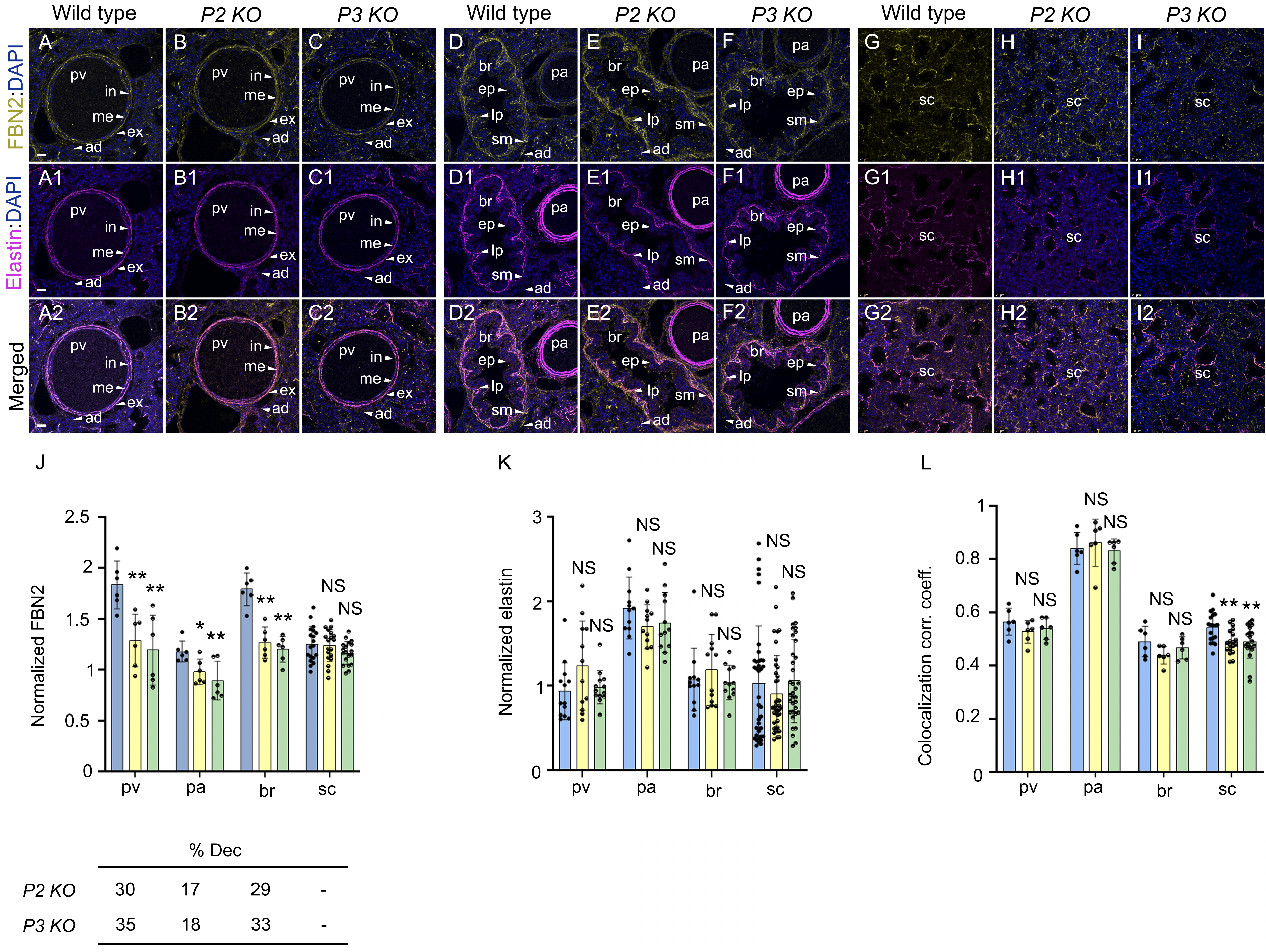


**Figure S5. Decreased FBN2 level in E18.5 *Poglut2 KO (P2 KO)* and *Poglut3 KO (P3 KO)* lung.** **(A-I2)** Representative maximum projection images comparing FBN2 (yellow) immunolocalization **(A-I)**, elastin (magenta) detection using alexafluor 633 (AF633) **(A1-I1)** with DAPI counterstaining (blue) and FBN2 and elastin colocalization **(A2-I2)** in E18.5 lung sections. **(J, K, L)** Quantification of FBN2 **(J)** and elastin (AF633) **(K)** signals normalized to DAPI and estimation of colocalization correlation (corr.) coefficient (coeff.) **(L)** in the pv, pa, br, and sc. Regions of interest (ROI) are defined in Fig. S4, A-H. The elastin graph is reused from Fig. S4. Percent decreased (% dec) from wild type indicated below graph. Wild type images from Fig. 5 were reused in this figure for comparison purposes. Data from wild type (blue column with solid black circles), *Poglut2 KO* (yellow column with half-open black circles) and *Poglut3 KO* (green column with open half-black circles) were evaluated for statistical significance using unpaired, two-tailed *t*-test: **p 0.01 and NS, not significant. Error bars show ± SD. Abbreviations: ad, adventitia; in, intima; me, media; ex, externa; ep, epithelium; sm, smooth muscle; lp, lamina propria. Scale bars: all panels 20 μm. Images were obtained from 3 embryos per genotype, 2-3 sections per embryo, and for pv, pa, and br regions 1 field per section and for sc region 2-3 fields per sections.


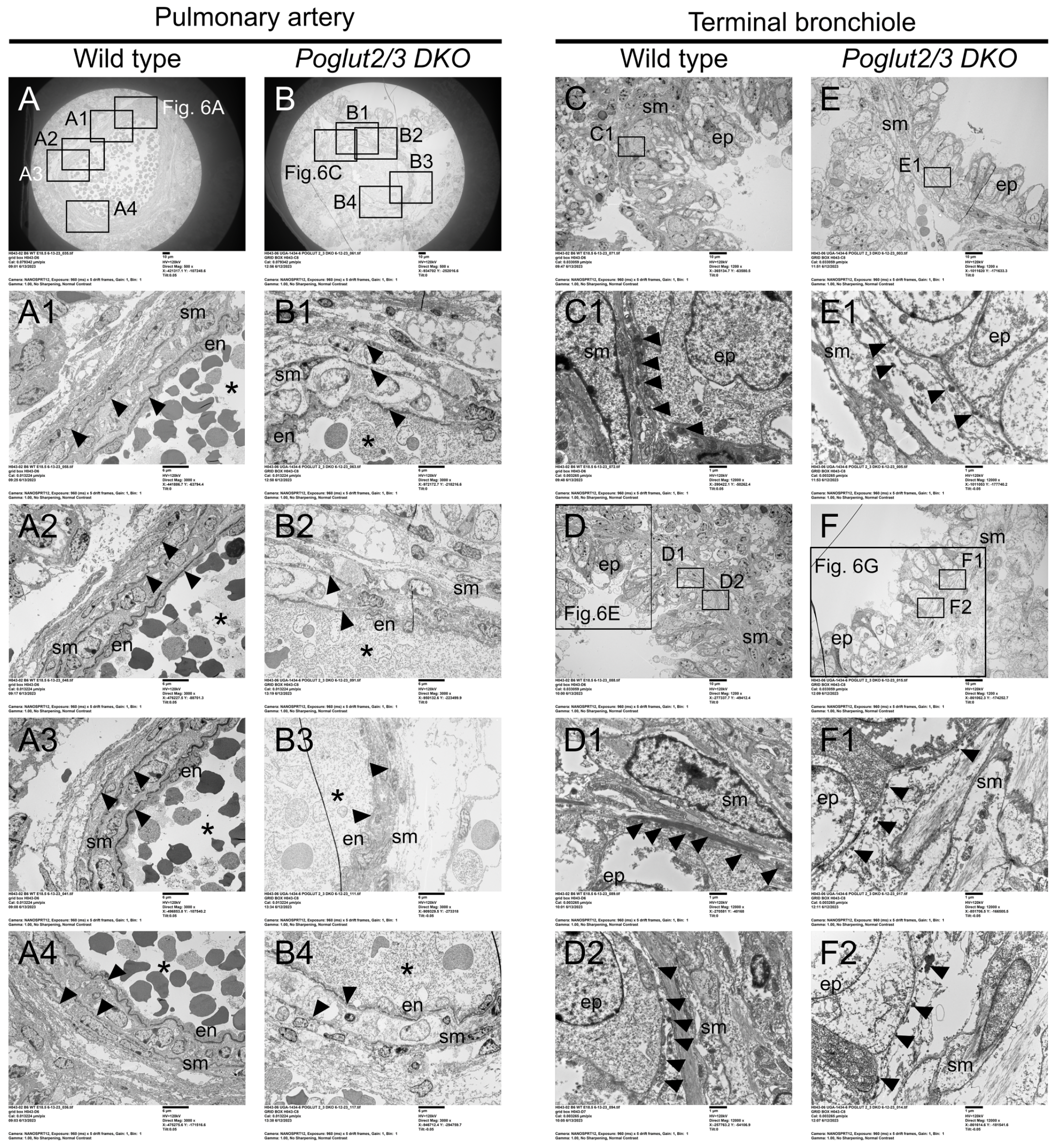


**Figure S6. Fragmented elastic lamella and reduced elastin in pulmonary artery and bronchiole of *Poglut2/3* *DKO* lung.** **(A-F)** Transmission electron micrograph of pulmonary artery **(A-B)** and bronchiole **(C-F)** from wild type (A,B) and *Poglut2/3 DKO* (C,D,E,F) lung. Asterisk (*) denotes artery lumen. **(A1-A4)** In wild type pulmonary artery, elastic lamella (el), indicated by arrowheads, were largely intact and distinctly visible. The internal elastic lamina separating the endothelium (en) and smooth muscle layer (sm) was largely intact. The middle elastic lamina separating smooth muscle layers and external elastic lamina separating smooth muscle and adventitia layers had occasional breaks. **(B1-B4)** In the *Poglut2/3 DKO*, all elastic lamellae were fragmented and with sparse elastic fibers in the outer layers. **(C-D2)** In wild type bronchiole elastin deposits were localized (indicated by arrowheads) in the region between the epithelium (ep) and smooth muscle (sm) layer. **(E-F2)** In *Poglut2/3* *DKO* elastin deposits were dispersed (indicated by arrowheads). Rectangles in A-F indicate the magnified regions in A1-A4, B1-B4, C1, D1-D2, E1, F1 and F2 and the locations of images used in Fig. 6 A, C, E, and H. Scale bars: Panels A to F 10 μm, panels A1-A4 and B1-B4 6 μm, C1, D1, D2, E1, F1 and F2 1 μm.

**
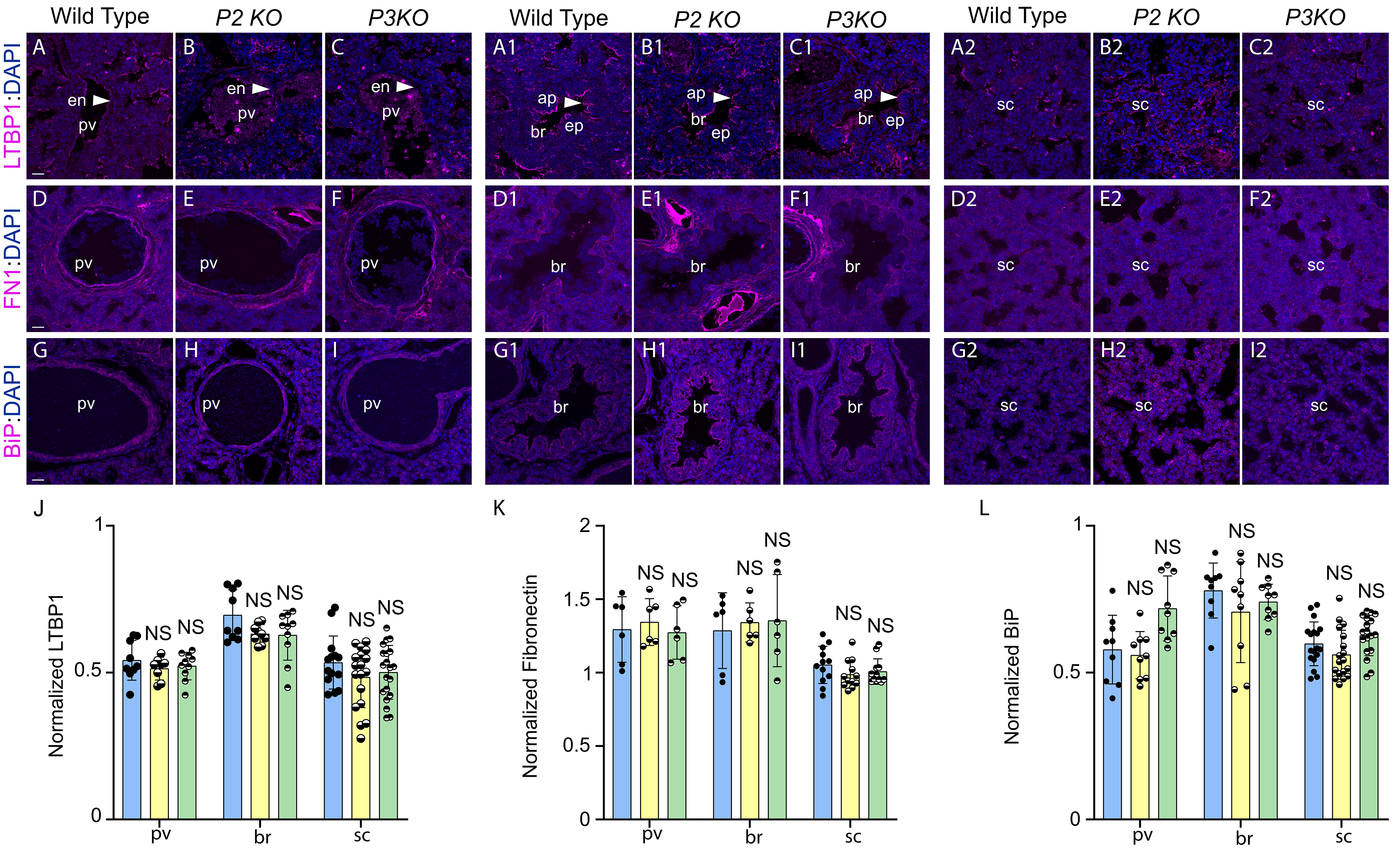
**

**Figure S7. Loss of *Poglut2 or Poglut3* had no effect on LTBP1 level at E18.5 lung**. **(A-C2)** Comparison of representative maximum projection images of LTBP1 (magenta) localization and counterstained with DAPI (blue) in E18.5 lung pulmonary vein (pv**,** A-C), bronchiole (br; A1-C1), and saccule (sc; A2-D2) regions from wild type, *P2 KO*, and *P3 KO*. **(A-A2)** In wild type lung, strong LTBP1 signal was observed in the pv endothelium (en), the br apical (ap) epithelial surface (ep), and in the interstitium of the sc region. **(B-B2, C-C2)** In *Poglut2 and Poglut3 KO* lung LTBP signal was similarly localized in pv, br, and sc and at comparable levels. **(D-F2)** Comparison of representative maximum projection images of fibronectin (magenta) localization and counterstained with DAPI (blue) showed similar staining in E18.5 lung blood vessel (pv; D-F), bronchiole (br; D1-F1) and saccules (sc; D2-F2) from wild type (**D-D2**), *P2 KO* (**E-E2**) and *P3 KO* (**F-F2**). **(G-I2)** Comparison of representative maximum projection images of BiP (magenta) localization and counterstained with DAPI (blue) showed similar staining and localization in E18.5 lung blood vessel (pv; G-I), bronchiole (br; G1-I1) and saccule (sc; G2-I2) from wild type (**G-G2**), *P2 KO* (**H-H2**) and *P3 KO* (**I-I2**). Wild type images from Fig. 7 were repeated in this figure for comparison purposes. **(J-L)** Quantification of LTBP1 (J), fibronectin (K) and BiP (L) immunofluorescence signals in the pv, br, and sc (ROIs defined in Fig. S4) from wild type, *P2 KO* and *P3 KO*. Immunofluorescence signals from individual image was normalized with DAPI signals from the same image. Data from wild type (blue column with solid black circles), *P2 KO* (yellow column with open-half black circle) and *P3 KO* (green column with half open-black circles) were evaluated for statistical significance using unpaired, two-tailed *t*-test: NS, not significant. Error bars show ± SD. Scale bars: all panels 20 μm. Images were obtained from 3 embryos per genotype, 2-3 sections per embryo, and for pv and br regions 1 field per section and for sc region 2-3 fields per sections.

**Supplementary Dataset legend**

**Dataset S1: MS/MS spectra of peptides from EGF repeats in mouse fibrillin-1 (11 EGFs), fibulin-2 (5 EGFs), fibulin-5 (1 EGF), and nidogen-1 (1 EGF) used for glycoproteomic analysis** (related to Figs. 2 and S2, Excel file 1). Endogenous proteins were analyzed from culture media of mouse lung fibroblasts established at E18.5 as described in Experimental Procedures. Slides are organized by protein and EGF number in sequential order. The amino acid sequence for each analyzed peptide is annotated with its corresponding b- and y-ions identified in the MS/MS spectrum (purple and blue, respectively). These MS/MS spectra are shown below the peptide sequence and are used to confirm the identity of each peptide. The serine (S) residue predicted to be modified by POGLUT2/3, based on the putative consensus sequence is underlined and colored in blue. A blue circle (symbol for *O-*glucose) is placed above the amino acid the analytical software indicated as modified. Due to the lability of *O-*glycans in the gas phase during collision, these modifications can fall off which can lead to incorrect annotation of the modified amino acid. However, we can predict the correct position based on the putative consensus sequence for modification by POGLUT2/3. Slides 2, 3, and 18 are examples where the software incorrectly identified the modified amino acid. A representative extracted ion chromatogram (EIC) is shown to the right of the MS/MS spectrum for each peptide. EICs provide quantification of the relative abundance of a specific peptide ion. The mass-to-charge ratio (m/z) of each analyzed peptide is shown in Excel file 1. POGLUT1-mediated *O-*glucosylation and POFUT1-mediated *O*-fucosylation were identified on fibrillin-1 (1 EGF and 2 EGFs, respectively) and were used as controls to indicate no changes in other forms of *O-*glycosylation in the *Poglut2/3 DKOs* (slide 19-21).

**Supplementary Excel file legends**

**Excel file 1: List of peptides used for glycoproteomic analysis to compare POGLUT2/3-mediated *O-*glycosylation of endogenous, secreted proteins from wild type and *Poglut2/3 DKO* lung fibroblasts at E18.5** (related to Figs. 2 and S2).

All samples were processed as described in Experimental Procedures. Modified peptides are annotated with the monoisotopic mass of each modification. Fixed modification: Carbamidomethylation [+57]. Variable modifications: *O-*glucose [+162.1], *O-*fucose [+146.1], and β-hydroxylation [+16]. *O-*glucosylated peptides are highlighted in dark blue, *O-*glucosylated plus β-hydroxylated peptides are highlighted in light blue, and *O-*fucosylated peptides are highlighted in red. Peptides without *O-*glycosylation are highlighted in dark grey. Peptides containing β-hydroxylation but no *O-*glycosylation are highlighted in light grey. Excel file Tab 1 contains wild type data, and Tab 2 contains *Poglut2/3 DKO* data.

**Excel file 2: Extracellular proteins identified by mass spectrometry from culture medium of wild type (WT) and *Poglut2/3 DKO* (*DKO*) dermal fibroblasts** (related to Fig. 8). Protein names in **bolded** font contain EGFs with a serine residue in the correct position to be modified by POGLUT2/3 (reference sequence: C^3^-x-x-x-x-x-S-x-x-C^4^). *Green shade*, proteins with statistically significant reduced abundance in *DKO*; *grey shade,* proteins with no statistically significant change in abundance; *red shade,* proteins with statistically significant increased abundance in *DKO*. Abbreviations: PSM, peptide-spectrum match.

**Excel file 3: Extracellular proteins identified by mass spectrometry from deposited extracellular matrix of wild type (WT) and *Poglut2/3 DKO* (*DKO*) dermal fibroblasts** (related to Fig. 8). Protein names in **bolded** font contain EGFs with a serine residue in the correct position to be modified by POGLUT2/3 (reference sequence: C^3^-x-x-x-x-x-S-x-x-C^4^). *Green shade*, proteins with statistically significant reduced abundance in *DKO*; *grey shade,* proteins with no statistically significant change in abundance; *red shade,* proteins with statistically significant increased abundance in *DKO*. Abbreviations: PSM, peptide-spectrum match.

**Excel file 4: summary of p-values from statistical analyses**

(related to Figs. 1, 2, 4, 5, 7, 8 and supplementary Figs 2, 4, 5, 7)
